# Supplementary material for: Successive cultivation under drought selects for specific microbiome members in the wheat rhizosphere
Source: FEMS Microbiol Ecol. 2026 Apr 14;102(5):fiag037. doi: 10.1093/femsec/fiag037 (PMC13114863; doi:10.1093/femsec/fiag037)
Supplement: fiag037_Supplemental_Files [file fiag037_supplemental_files.zip › Dataset S1.docx]

>A1_Stenotrophomonas_maltophilia

CGGTGGCAGCGCCCTCCCGAAGGTTAAGCTACCTGCTTCTGGTGCAACAAACTCCCATGG

TGTGACGGGCGGTGTGTACAAGGCCCGGGAACGTATTCACCGCAGCAATGCTGATCTGCG

ATTACTAGCGATTCCGACTTCATGGAGTCGAGTTGCAGACTCCAATCCGGACTGAGATAG

GGTTTCTGGGATTGGCTTACCGTCGCCGGCTTGCAGCCCTCTGTCCCTACCATTGTAGTA

CGTGTGTAGCCCTGGCCGTAAGGGCCATGATGACCTGACGTCATCCCCCACCTTCCTCCG

GTTTGTCACCGGCGGTCTCCTTAGAGTTCCCACCATTACGTGCTGGCAACTAAGGACAAG

GGTTGCGCTCGTTGCGAGACTTAACCCAACATCTCACGACACGAGCTGACGACAGCCATG

CAGCACCTGTGTTCGAGTTCCCGAAGGCACCAATCCATCTCTGGAAAGTTCTCGACATGT

CAAGGCCAGGTAAGGTTCTTCGCGTTGCATCGAATTAAACCACATACTCCACCGCTTGTG

CGGGCCCCCGTCAATTCCTTTGAGTTTCAGTCTTGCGACCGTACTCCCCAGGCGGCGAAC

TTAACGCGTTAGCTTCGATACTGCGTGCCAAATTGCACCCAACATCCAGTTCGCATCGTT

TAGGGCGTGGACTACCAGGGTATCTAATCCTGTTTGCTCCCCACGCTTTCGTGCCTCAGT

GTCAGTGTTGGTCCAGGTAGCTGCCTTCGCCATGGATGTTCCTCCCGATCTCTACGCATT

TCACTGCTACACCGGGAATTCCGCTACCCTCTACCACACTCTAGTTGTCCAGTTTCCACT

GCAGTTCCCAGGTTGAGCCCAGGGCTTTCACAACAGACTTAAACAACCACCTACGCACGC

TTTACGCCCAGTAATTCCGAGTAACGCTTGCACCCTTCGTATTACCGCGGCTGCTGGCAC

GAAGTTAGCCGGTGCTTATTCTTTGGGTACCGTCATCCCAACCAGGTATTAGCCGGCTGG

ATTTCTTTCCCAACAAAAGGGCTTTACAACCCGAAGGCCTTCTTCACCCACGCGGTATGG

CTGGATCAGGCTTGCGCCCATTGTCCAATATTCCCCACTGCTGCCTCCCGTAGGAGTCTG

GACCGTGTCTCAGTTCCAGTGTGGCTGATCATCCTCTCAGACCAGCTACGGATCGTCGCC

TTGGTGGGCCTTTACCCCGCCAACTAGCTAATCCGAAATCGGCTCATTCAATCGCGCAAG

GCCCGAAGGTCCCCTGCTTTCACCCGTAGGTCGTATGCGGTATTAGCGTAAGTTTCCCTA

CGTTATCCCCCACGAAAAAGTAGATTCCGATGTATTCCTCACCCGTCCGCCACTCGCCAC

CCAAGGAGCAAGCTCCTCTGTGCTGCCGTTCGACTTGCAT

>A2_Streptomyces_sp.

GTCCCCTTCGAACGCTCCCTCCACAAGGGGTTGGGCCACCGGCTTCGGGTGTTACCGACT

TTCGTGACGTGACGGGCGGTGTGTACAAGGCCCGGGAACGTATTCACCGCAGCAATGCTG

ATCTGCGATTACTAGCAACTCCGACTTCATGGGGTCGAGTTGCAGACCCCAATCCGAACT

GAGACCGGCTTTTTGAGATTCGCTCCGCCTCGCGGCATCGCAGCTCATTGTACCGGCCAT

TGTAGCACGTGTGCAGCCCAAGACATAAGGGGCATGATGACTTGACGTCGTCCCCACCTT

CCTCCGAGTTGACCCCGGCAGTCTCCTGTGAGTCCCCATCACCCCGAAGGGCATGCTGGC

AACACAGAACAAGGGTTGCGCTCGTTGCGGGACTTAACCCAACATCTCACGACACGAGCT

GACGACAGCCATGCACCACCTGTATACCGACCACAAGGGGGGCACCATCTCTGATGCTTT

CCGGTATATGTCAAGCCTTGGTAAGGTTCTTCGCGTTGCGTCGAATTAAGCCACATGCTC

CGCTGCTTGTGCGGGCCCCCGTCAATTCCTTTGAGTTTTAGCCTTGCGGCCGTACTCCCC

AGGCGGGGAACTTAATGCGTTAGCTGCGGCACCGACGACGTGGAATGTCGCCAACACCTA

GTTCCCAACGTTTACGGCGTGGACTACCAGGGTATCTAATCCTGTTCGCTCCCCACGCTT

TCGCTCCTCAGCGTCAGTAATGGCCCAGAGATCCGCCTTCGCCACCGGTGTTCCTCCTGA

TATCTGCGCATTTCACCGCTACACCAGGAATTCCGATCTCCCCTACCACACTCTAGCTAG

CCCGTATCGAATGCAGACTCGGGGTTAAGCCCCGAGCTTTCACATCCGACGTGACAAGCC

GCCTACGAGCTCTTTACGCCCAATAATTCCGGACAACGCTCGCACCCTACGTATTACCGC

GGCTGCTGGCACGTAGTTAGCCGGTGCTTCTTCTGCAGGTACCGTCACTTGCGCTTCTTC

CCTGCTGAAAGAGGTTTACAACCCGAAGGCCGTCATCCCTCACGCGGCGTCGCTGCATCA

GGCTTTCGCCCATTGTGCAATATTCCCCACTGCTGCCTCCCGTAGGAGTCTGGGCCGTGT

CTCAGTCCCAGTGTGGCCGGTCGCCCTCTCAGGCCGGCTACCCGTCGTCGCCGTGGTAGG

CCATTACCCCACCAACAAGCTGATAGGCCGCGGGCTCATCCTTCACCGCCGGAGCTTTTA

ACCCCGCCCCATGAGGGACAGAGTGTTATCCGGTATTAGACCCCGTTTCCAGGGCTTGTC

CCAGAGTGAAGGGCAGATTGCCCACGTGTTACTCACCCGTTCGCCACTAATCCACCCCGA

AGGGCTTCATCGTTCGACTTGCA

>A3_Variovorax_boronicumulans

CCAACCTGCCGTGGTATCGCCCTCCTTGCGGTTAAGCTAACTACTTCTGGCAGAACCCGC

TCCCATGGTGTGACGGGCGGTGTGTACAAGACCCGGGAACGTATTCACCGTGACATTCTG

ATCCACGATTACTAGCGATTCCGACTTCACGCAGTCGAGTTGCAGACTGCGATCCGGACT

ACGACTGGTTTTATGGGATTAGCTCCCCCTCGCGGGTTGGCAACCCTTTGTACCAGCCAT

TGTATGACGTGTGTAGCCCCACCTATAAGGGCCATGAGGACTTGACGTCATCCCCACCTT

CCTCCGGTTTGTCACCGGCAGTCTCATTAGAGTGCCCAACTGAATGTAGCAACTAATGAC

AAGGGTTGCGCTCGTTGCGGGACTTAACCCAAACATCTTCACGACACGAGCTGACGACAG

CCATGCAGCACCTGTGTTACGGTTCTCTTTCGAGCACTAAGCCATCTCTGGCGAATTCCG

TACATGTCAAAGGTGGGTAAGGTTTTTCGCGTTGCATCGAATTAAACCACATTCATCCAC

CGCTTGTGCGGGTCCCCGTCAATTCCTTTGAGTTTCAACCTTGCGGCCGTACTCCCCAGG

CGGTCAACTTCACGCGTTAGCTTCGTTACTGAGTCAGTGAAGACCCAACAACCAGTTGAC

ATCGTTTAGGGCGTGGACTACCAGGGTATCTAATCCTGTTTGCTCCCCACGCTTTCGTGC

ATGAGCGTCAGTACAGGTCCAGGGGATTGCCTTCGCCATCGGTGTTCCTCCGCATATCTA

CGCATTTCACTGCTACACGCGGAATTCCATCCCCCTCTACCGTACTCTAGCTATGCAGTC

ACAGATGCAGTTCCCAGGTTGAGCCCGGGGATTTCACAACTGTCTTACATAACCGCCTGC

GCACGCTTTACGCCCAGTAATTCCGATTAACGCTTGCACCCTACGTATTACCGCGGCTGC

TGGCACGTAGTTAGCCGGTGCTTATTCTTACGGTACCGTCATTAGCCTTCTGTATTAGAA

AAGACCGTTTCGTTCCGTACAAAAGCAGTTTACAACCCGAAGGCCTTCATCCTGCACGCG

GCATGGCTGGATCAGGCTTTCGCCCATTGTCCAAAATTCCCCACTGCTGCCTCCCGTAGG

AGTCTGGGCCGTGTCTCAGTCCCAGGGTGGCTGGTCGTCCTCTCAGACCAGCTACAGATC

GAAGGCTTGGTGAGCCTTTACCTCACCAACTACCTAATCTGCCATCGGCCGCTCCATTCG

CGCAAGGTCTTGCGATCCCCTGCTTTCATCCGTAGATCGTATGCGGTATTAGCACAGCTT

TCGCTGCGTTATCCCCCACGATTGGGCACGTTCCGATGTATTACTCACCCGTTCGCCACT

CGCCGCCAGGATTGCTCCCGCGCTGCCGTTCGACTTGCAT

>A4_Streptomyces_sp.

CACCTTCGACAGCTCCCTCCCACAAGGGGTTGGGCCACCGGCTTCGGGTGTTACCGACTT

TCGTGACGTGACGGGCGGTGTGTACAAGGCCCGGGAACGTATTCACCGCAGCAATGCTGA

TCTGCGATTACTAGCAACTCCGACTTCATGGGGTCGAGTTGCAGACCCCAATCCGAACTG

AGACCGGCTTTTTGAGATTCGCTCCGCCTCGCGGCATCGCAGCTCATTGTACCGGCCATT

GTAGCACGGGTGCAGCCCAAGACATAAGGGGCATGATGACTTGACGTCGTCCCCACCTTC

CTCCGAGTTGACCCCGGCAGTCTCCTGTGAGTCCCCATCACCCCGAAGGGCATGCTGGCA

ACACAGAACAAGGGTTGCGCTCGTTGCGGGACTTAACCCAACATCTCACGACACGAGCTG

ACGACAGCCATGCACCACCTGTCACCCGACCACAAGGGGGGCCGTATCTCTACGGCTTTC

CGGGCGATGTCAAGCCTTGGTAAGGTTCTTCGCGTTGCGTCGAATTAAGCCACATGCTCC

GCTGCTTGTGCGGGCCCCCGTCAATTCCTTTGAGTTTTAGCCTTGCGGCCGTACTCCCCA

GGCGGGGAACTTAATGCGTTAGCTGCGGCACCGACGACGTGGAATGTCGCCAACACCTAG

TTCCCAACGTTTACGGCGTGGACTACCAGGGTATCTAATCCTGTTCGCTCCCCACGCTTT

CGCTCCTCAGCGTCAGTAATGGCCCAGAGATCCGCCTTCGCCACCGGTGTTCCTCCTGAT

ATCTGCGCATTTCACCGCTACACCAGGAATTCCGATCTCCCCTACCACACTCTAGCCTGC

CCGTATCGACTGCAGACCCGGGGTTAAGCCCCGGGCTTTCACAACCGACGCAACAAGCCG

CCTACGAGCTCTTTACGCCCAATAATTCCGGACAACGCTTGCGCCCTACGTATTACCGCG

GCTGCTGGCACGTAGTTAGCCGGCGCTTCTTCTGCAGGTACCGTCACTTTCGCTTCTTCC

CTGCTGAAAGAGGTTTACAACCCGAAGGCCGTCATCCCTCACGCGGCGTCGCTGCATCAG

GCTTTCGCCCATTGTGCAATATTCCCCACTGCTGCCTCCCGTAGGAGTCTGGGCCGTGTC

TCAGTCCCAGTGTGGCCGGTCGCCCTCTCAGGCCGGCTACCCGTCGTCGCCTTGGTAGGC

CATTACCCCACCAACTAGCTGAAAGGCCGCGGGCTCATCCTTCACCGCCGGAGCTTTTAA

CCTTCCCCCATGAGAGAGAAAGTATTATCCGGTATTAGACCCCGTTTCCAGGGCTTGTCC

CAGAGTGAAGGGCAGATTGCCCACGTGTTACTCACCCGTTCGCCACTAATCCACCCCGAA

AGGCTTCATCGTTCGACTTGCATGTTTAAG

>A5_Streptomyces_sp.

CCTTCGAAGCTCCCTCCCACAAGGGGTTGGGCCACCGGCTTCGGGTGTTACCGACTTTCG

TGACGTGACGGGCGGTGTGTACAAGGCCCGGGAACGTATTCACCGCAGCAATGCTGATCT

GCGATTACTAGCAACTCCGACTTCATGGGGTCGAGTTGCAGACCCCAATCCGAACTGAGA

CCGGCTTTTTGAGATTCGCTCCGCCTCGCGGCATCGCAGCTCATTGTACCGGCCATTGTA

GCACGTGTGCAGCCCAAGACATAAGGGGCATGATGACTTGACGTCGTCCCCACCTTCCTC

CGAGTTGACCCCGGCAGTCTCCTGTGAGTCCCCATCACCCCGAAGGGCATGCTGGCAACA

CAGAACAAGGGTTGCGCTCGTTGCGGGACTTAACCCAACATCTCACGACACGAGCTGACG

ACAGCCATGCACCACCTGTATACCGACCACAAGGGGGGCACCATCTCTGATGCTTTCCGG

TATATGTCAAGCCTTGGTAAGGTTCTTCGCGTTGCGTCGAATTAAGCCACATGCTCCGCT

GCTTGTGCGGGCCCCCGTCAATTCCTCTGAGTTTTAGCCTTGCGGCCGTACTCCCCAGGC

GGGGAACTTAATGCGTTAGCTGCGGCACCGACGACGTGGAATGTCGCCAACACCTAGTTC

CCAACGTTTACGGCGTGGACTACCAGGGTATCTAATCCTGTTCGCTCCCCACGCTTTCGC

TCCTCAGCGTCAGTAATGGCCCAGAGATCCGCCTTCGCCACCGGTGTTCCTCCTGATATC

TGCGCATTTCACCGCTACACCAGGAATTCCGATCTCCCCTACCACACTCTAGCTAGCCCG

TATCGAATGCAGACCCGGGGTTAAGCCCCGGGCTTTCACATCCGACGTGACAAGCCGCCT

ACGAGCTCTTTACGCCCAATAATTCCGGACAACGCTTGCGCCCTACGTATTACCGCGGCT

GCTGGCACGTAGTTAGCCGGCGCTTCTTCTGCAGGTACCGTCACTTTCGCTTCTTCCCTG

CTGAAAGAGGTTTACAACCCGAAGGCCGTCATCCCTCACGCGGCGTCGCTGCATCAGGCT

TTCGCCCATTGTGCAATATTCCCCACTGCTGCCTCCCGTAGGAGTCTGGGCCGTGTCTCA

GTCCCAGTGTGGCCGGTCGCCCTCTCAGGCCGGCTACCCGTCGTCGCCTTGGTAGGCCAT

TACCCCACCAACAAGCTGATAGGCCGCGGGCTCATCCTTCACCGCCGGAGCTTTTAACCC

CGTCCCATGCGGGACAGAGTGTTATCCGGTATTAGACCCCGTTTCCAGGGCTTGTCCCAG

AGTGAAGGGCAGATTGCCCACGTGTTACTCACCCGTTCGCCACTAATCCACCCCGAAAGG

CTTCATCGTTCGACTTGCATGTGTTAAGCCCCCC

>A6_Agrobacterium_tumefaciens

TAGCTGCCTCCTTGCGGTTAGCGCACTACCTTCGGGTAAAACCAACTCCCATGGTGTGAC

GGGCGGTGTGTACAAGGCCCGGGAACGTATTCACCGCAGCATGCTGATCTGCGATTACTA

GCGATTCCAACTTCATGCACTCGAGTTGCAGAGTGCAATCCGAACTGAGAAGGCTTTTGG

AGATTAGCTCGACATCGCTGTTCTCGCTGCCCACTGTCACCACCATTGTAGCACGTGGGT

AGCCCAGCCCGTAAGGGCCCATGAGGACTTGACGTCATCCCCACCTTTCCTCTCGGGCTT

ATCACCGGCAGTCCCCTTAGAGTGCCCAACTAAATGCTGGCAACTAAGGGCGAGGGTTGC

GCTCGTTGCGGGACTTAACCCAAACATCTCACGACACGAGCTGACGACAGCCATGCAGCA

CCTGTTCTGGGGCCAGCCTAACTGAAGGACAATGTCTCCACTGCCCAAACCCCGAAAGTC

AAGAGCTGGTAAGGTTCTGCGCGTTGCTTCGAATTAAACCACATGCTCCACCGCTTGTGC

GGGCCCCCGTCAATTCCTTTGAGTTTTAATCTTGCGACCGTACTCCCCAGGCGGAATGTT

TAATGCGTTAGCTGCGCCACCGAACAGTATACTGCCCGACGGCTAACATTCATCGTTTAC

GGCGTGGACTACCAGGGTATCTAATCCTGTTTGCTCCCCACGCTTTCGCACCTCAGCGTC

AGTAATGGACCAGTAAGCCGCCTTCGCCACTGGTGTTCCTCCGAATATCTACGAATTTCA

CCTCTACACTCGGAATTCCACTTACCTCTTCCATACTCAAGATACCCAGTATCAAAGGCA

GTTCCAGAGTTGAGCTCTGGGATTTCACCCCTGACTTAAATATCCGCCTACGTGCGCTTT

ACGCCCAGTAATTCCGAACAACGCTAGCCCCCTTCGTATTACCGCGGCTGCTGGCACGAA

GTTAGCCGGGGCTTCTTCTCCGGATACCGTCATTATCTTCTCCGGTGAAAGAGCTTTACA

ACCCTAAGGCCTTCATCACTCACGCGGCATGGCTGGATCAGGCTTGCGCCCATTGTCCAA

TATTCCCCACTGCTGCCTCCCGTAGGAATTTGGGCCGTGTCTCAGTCCCAATGTGGCTGA

TCATCCTCTCAGAACAGCTATGGATCGTCGCCTTGGTAGGCCTTTACCCCACCAACTAGC

TAATCCAACGCGGGCTCATCAAACCCCGATAAATCTTTCCCCCGTAGGGCGTATGCGGTA

TTAATTCCAGTTTCCCGGAGCTATTCCGCAGGGCACGGTAGATTCCCACGCGTTACTCAC

CCGTCTGCCACTCCCCTTGCGGGGCGTTCGACTTGCATGTGTAAGCCCCCCGA

>A7_Stenotrophomonas_sp.

GGCAGCGCCCTCCCGAAGGTTAAGCTACCTGCTTCTGGTGCAACAAACTCCCATGGTGTG

ACGGGCGGTGTGTACAAGGCCCGGGAACGTATTCACCGCAGCAATGCTGATCTGCGATTA

CTAGCGATTCCGACTTCATGGAGTCGAGTTGCAGACTCCAATCCGGACTGAGATAGGGTT

TCTGGGATTGGCTTGCCCTCGCGGGTTTGCAGCCCTCTGTCCCTACCATTGTAGTACGTG

TGTAGCCCTGGTCGTAAGGGCCATGATGACTTGACGTCATCCCCACCTTCCTCCGGTTTG

TCACCGGCGGTCTCCTTAGAGTTCCCACCATTACGTGCTGGCAACTAAGGACAAGGGTTG

CGCTCGTTGCGGGACTTAACCCAACATCTCACGACACGAGCTGACGACAGCCATGCAGCA

CCTGTGTTCGAGTTCCCGAAGGCACCAATCCATCTCTGGAAAGTTCTCGACATGTCAAGA

CCAGGTAAGGTTCTTCGCGTTGCATCGAATTAAACCACATACTCCACCGCTTGTGCGGGC

CCCCGTCAATTCCTTTGAGTTTCAGTCTTGCGACCGTACTCCCCAGGCGGCGAACTTAAC

GCGTTAGCTTCGATACTGCGTGCCAAATTGCACCCAACATCCAGTTCGCATCGTTTAGGG

CGTGGACTACCAGGGTATCTAATCCTGTTTGCTCCCCACGCTTTCGTGCCTCAGTGTCAG

TGTTGGTCCAGGTAGCTGCCTTCGCCATGGATGTTCCTCCCGATCTCTACGCATTTCACT

GCTACACCGGGAATTCCACTACCCTCTACCACACTCTAGTCGCCCAGTATCCACTGCAAT

TCCCAGGTTGAGCCCAGGGCTTTCACAACAGACTTAAACAACCACCTACGCACGCTTTAC

GCCCAGTAATTCCGAGTAACGCTTGCACCCTTCGTATTACCGCGGCTGCTGGCACGAAGT

TAGCCGGTGCTTATTCTTTGGGTACCGTCAGAACAACCGGGTATTAGCCGACTGCTTTTC

TTTCCCAACAAAAGGGCTTTACAACCCGAAGGCCTTCTTCACCCACGCGGTATGGCTGGA

TCAGGCTTGCGCCCATTGTCCAATATTCCCCACTGCTGCCTCCCGTAGGAGTCTGGACCG

TGTCTCAGTTCCAGTGTGGCTGATCATCCTCTCAGACCAGCTACGGACCGTTCGCCTTGG

TGGGCCTTTACCCCGCCAACTAGCTAATCCGACATCGGCTCATCTATCCGCGCAAGGCCC

GAAGGTCCCCTGCTTTCACCCGAAGGTCGTATGCGGTATTAGCGTAAGTTTCCCTACGTT

ATCCCCCACGAAAAGGTAGATTCCGATGTATTCCTCACCCGTCCGCCACTCGCCACCCAT

AAGAGCAAGCTCTTACTGTGCTGCCGTTCGACTGCATGT

>A8_Ensifer_sp.

GGAGTGTACAAGGCCCGGGAACGTATTCACCGCAGCAGGCTGATCTGCGATTACAAGCGT

ATTCCAACTTCATGAACTCGAGTTGCAGAGTGCAATCCGAACTGAGATGGCTTTTGGAGA

ATAACTCGACCTCCCGGTCTCGCTGCCCACTGTCACCACCATTGGAGCACGAGGGGAGCC

CAACCCGTAAGGGCCATGAAGACTTGACGTCATCCCCACCTTCCTCTCGGCTTATCACCG

GCAGTCCCCTTAGAGTGCCCAACTAAATGCGGGCAACTAAAGGCGAAGGTTGCGCTCGTT

GCGGGACTTAACCCAACATCTCACGACACGAGCTGACGACAGCCATGCACCACCTGTCTC

CGATCCAGCCGAACTGAAGGAATACATCTCTGTAATCCGCAATCGGGATGACAAGGGCTG

GAAAGGTTCTGCGCGTTGCTTCAAATTAAACCACATGCTCCACCGCTTGTGCGGGCCCCC

GTCAATTCCTTTGAGTTATAATCTTGCGACCGTACTCCCCAGGCGGAATGTTTAATGCGT

TAGCTGCGCCACCGAACACTAAACTGCCCGACGGCTAACATTCATCGTTTACGGCGGGAA

CTACCAGGGTATCTAATCCTGTTTGCTCCCCACGCTTTCGAACCTCAGCGTCAGTAATGG

ACCAGTGAGCCGCCTTCGCCACTGGGTGTTCCTCCGAATATCTACGAATTTCACCTCTAC

ACTCGGAATTCCACTCACCTCTTCCATACTCTAGACACCCAGTATCAAAGGCAGTTCCAG

AGTTGAGCTCTGGGATTTCACCCCTGACTTAAATGTCCGCCTACGTGCGCTTTACGCCCA

GTAATTCCGAACAACGCTAGCCCCCTTCGTATTACCGCGGCTGCTGGCACGAAGTTAGCC

GGGGCTTCTTCTCCGGTTACCGTCATTATCTTCACCGGTGAAAGAGCTTTACAACCCTAG

GGCCTTCATCACTCACGCGGCATGGCTGGATCAGGCTTGCGCCCATTGTCCAATATTCCC

CACTGCTGCCTCCCGTAGGAGTTTGGGCCGTGTCTCAGTCCCAATGTGGCTGATCATCCT

CTCAGACCAGCTATGGATCGTCGCCTTGGTAGGCCTTTACCCCACCAACTAGCTAATCCA

ACGCGGGCTCATCCTTTCCCGATAAATCTTTCCCCCGAAGGGCTCATACGGTATTAGCAC

ACGTTTCCATGCGTTATTCCGTAGAAAAGGGTAGATTCCCACGCGTTACTCACCCGTCTG

CCGCTCCCCTTGCGGGGCGCTCGACTGCA

>A9_Agrobacterium_tumefaciens

ACTACCGGTGGTTAGCTGCCTCCTTGCGGTTAGCGCACTACCTTCGGGTAAACCAACTCC

CATGGTGTGACGGGCGGTGTGTACAAGGCCCGGGAACGTATTCACCGCAGCATGCTGATC

TGCGATTACTAGCGATTCCAACTTCATGCACTCGAGTTGCAGAGTGCAATCCGAACTGAG

AAGGCTTTTGGAGATTAGCTCGACATCGCTGTCTCGCTGCCCACTGTCACCACCAATGTT

AGCACGTGTGTAGCCCAGCCCGTAAGGGCCATGAGGACTTGACGTTCATTCCCCACCTTC

CTCCCGGCTTATCACCGGCAGTCCCCTTAGAGTGCCCAACTAAATGCTGGCAACTAAGGG

CGAGGGTTGCGCTCGTTGCGGGACTTAACCCAAACATCTCACGACACGAGCTGACGACAG

CCATGCAGCACCTGTTCTGGGGCCAGCCTAACTGAAGGACAATGTCTCCACTGCCCAAAC

CCCGAATGTCAAGAGCTGGTAAGGTTCTGCGCGTTGCTTCGAATTAAACCACATGCTCCA

CCGCTTGTGCGGGCCCCCGTCAATTCCTTTGAGTTTTAATCTTGCGACCGTACTCCCCAG

GCGGAATGTTTAATGCGTTAGCTGCGCCACCGAACAGTATACTGCCCGACGGCTAACATT

CATCGTTTACGGCGTGGACTACCAGGGTATCTAATCCTGTTTGCTCCCCACGCTTTCGCA

CCTCAGCGTCAGTAATGGACCAGTAAGCCGCCTTCGCCACTGGTGTTCCTCCGAATATCT

ACGAATTTCACCTCTACACTCGGAATTCCACTTACCTCTTCCATACTCAAGATACCCAGT

ATCAAAGGCAGTTCCAGAGTTGAGCTCTGGGATTTCACCCCTGACTTAAATATCCGCCTA

CGTGCGCTTTACGCCCAGTAATTCCGAACAACGCTAGCCCCCTTCGTATTACCGCGGCTG

CTGGCACGAAGTTAGCCGGGGCTTCTTCTCCGGATACCGTCATTATCTTCTCCGGTGAAA

GAGCTTTACAACCCTAAGGCCTTCATCACTCACGCGGCATGGCTGGATCAGGCTTGCGCC

CATTGTCCAATATTCCCCACTGCTGCCCCCCGTAGGAATTTGGGCCGTGTCTCAGTCCCA

ATGTGGCTGATCATCCTCTCAGACAAGCTATGGATCGTCGCCTTGGTAGGCCTTTACCCC

ACCAACTAGCTAATCCAACGCGGGCTCATCATACCCCGATAAATCTTTCCCCCGTAGGGC

GTATGCGGTATTAATTCCAGTTTCCCGGAGCTATTCCGCAGGGCACGGTAGATTCCCACG

CGTTACTCACCCGTCTGCCACTCCCCTTGCGGGGCGTTCGACTTGCATGTGTAAGCC

>A10_Bacillus_sp.

GTCACTTAGGCGGCTGGGCTCCATAAAGGTTTACCTCACCGACTTCGGGTGTTACAAACT

CTCGTGGTGTGACGGGCGGTGTGTACAAGGCCCGGGAACGTATTCACCGCGGCATGCTGA

TCCGCGATTACTAGCGATTCCGGCTTCATGTAGGCGAGTTGCAGCCTACAATCCGAACTG

AGAATGGCTTTATGGGATTCGCTTACCTTCGCAGGTTTGCAGCCCTTTGTACCATCCATT

GTAGCACGTGTGTAGCCCAGGTCATAAGGGGCATGATGATTTGACGTCATCCCCACCTTC

CTCCGGTTTGTCACCGGCAGTCACCTTAGAGTGCCCAACTGAATGCTGGCAACTAAGATC

AAGGGTTGCGCTCGTTGCGGGACTTAACCCAACATCTCACGACACGAGCTGACGACAACC

ATGCACCACCTGTCACTCTGTCCCCCGAAGGGGAAAGCCCTATCTCTAGGGTTGTCAGAG

GATGTCAAGACCTGGTAAGGTTCTTCGCGTTGCTTCGAATTAAACCACATGCTCCACCGC

TTGTGCGGGCCCCCGTCAATTCCTTTGAGTTTCAGCCTTGCGGCCGTACTCCCCAGGCGG

AGTGCTTAATGCGTTAGCTGCAGCACTAAAGGGCGGAAACCCTCTAACACTTAGCACTCA

TCGTTTACGGCGTGGACTACCAGGGTATCTAATCCTGTTTGCTCCCCACGCTTTCGCGCC

TCAGTGTCAGTTACAGACCAGAAAGTCGCCTTCGCCACTGGTGTTCCTCCAAATCTCTAC

GCATTTCACCGCTACACTTGGAATTCCACTTTCCTCTTCTGCACTCAAGTTCCCCAGTTT

CCAATGACCCTCCACGGTTGAGCCGTGGGCTTTCACATCAGACTTAAGGAACCACCTGCG

CGCGCTTTACGCCCAATAATTCCGGACAACGCTTGCCACCTACGTATTACCGCGGCTGCT

GGCACGTAGTTAGCCGTGGCTTTCTGGTTAGGTACCGTCAAGGTACCAGCAGTTACTCTG

GTACTTGTTCTTCCCTAACAACAGAACTTTACGACCCGAAGGCCTTCTTCGTTCACGCGG

CGTTGCTCCGTCAGACTTTCGTCCATTGCGGAAGATTCCCTACTGCTGCCTCCCGTAGGA

GTCTGGGCCGTGTCTCAGTCCCAGTGTGGCCGATCACCCTCTCAGGTCGGCTACGCATCG

TCGCCTTGGTGAGCCATTACCTCACCAACTAGCTAAGGCGCCGCGGGCCCATCTATAAGT

GAAAGCGTAAACCGTCTTTCCATCTTCTCTCATGCGAGAAAAGAACGTATCCGGTATTAG

CTCCGGTTTCCCGAAGTTATCCCAGTCTTATAGGCAGGTTGCCCACGTGTTACTCACCCG

TCCGCCGCTAATCTCAGGGAGCAAGCTCCCATCGATCGCTCGACTTGCA

>A11_Pseudomonas_brassicacearum

ATGCAAGTCGAGCGGTAGAGAGGTGCTTGCACCTCTTGAGAGCGGCGGACGGGTGAGTAA

TGCCTAGGAATCTGCCTGGTAGTGGGGGATAACGCTCGGAAACGGACGCTAATACCGCAT

ACGTCCTACGGGAGAAAGCAGGGGACCTTCGGGCCTTGCGCTATCAGATGAGCCTAGGTC

GGATTAGCTAGTTGGTGGGGTAATGGCTCACCAAGGCGACGATCCGTAACTGGTCTGAGA

GGATGATCAGTCACACTGGAACTGAGACACGGTCCAGACTCCTACGGGAGGCAGCAGTGG

GGAATATTGGACAATGGGCGAAAGCCTGATCCAGCCATGCCGCGTGTGTGAAGAAGGTCT

TCGGATTGTAAAGCACTTTAAGTTGGGAGGAAGGGCATTAACCTAATACGTTAGTGTTTT

GACGTTACCGACAGAATAAGCACCGGCTAACTCTGTGCCAGCAGCCGCGGTAATACAGAG

GGTGCAAGCGTTAATCGGAATTACTGGGCGTAAAGCGCGCGTAGGTGGTTCGTTAAGTTG

GATGTGAAATCCCCGGGCTCAACCTGGGAACTGCATTCAAAACTGTCGAGCTAGAGTATG

GTAGAGGGTGAGTGGAATTTCATGTGTAGCGGTGAAATGCGTAGATATAGGAAGGAACAC

CAGTGGCGAAGGCGACCACCTGGACTGATACTGACACTGAGGTGCGAAAGCGTGGGGAGC

AAACAGGATTAGATACCCTGGTAGTCCACGCCGTAAACGATGTCAACTAGCCGTTGGGAG

CCTTGAGCTCTTAGTGGCGCAGCTAACGCATTAAGTTGACCGCCTGGGGAGTACGGCCGC

AAGGTTAAAACTCAAATGAATTGACGGGGGCCCGCACAAGCGGTGGAGCATGTGGTTTAA

TTCGAAGCAACGCGAAGAACCTTACCAGGCCTTGACATCCAATGAACTTTCCAGAGATGG

ATTGGTGCCTTCGGGAACATTGAGACAGGTGCTGCATGGCTGTCGTCAGCTCGTGTCGTG

AGATGTTGGGTTAAGTCCCGTAACGAGCGCAACCCTTGTCCTTAGTTACCAGCACGTTAT

GGTGGGCACTCTAAGGAGACTGCCGGTGACAAACCGGAGGAAGGTGGGGATGACGTCAAG

TCATCATGGCCCTTACGGCCTGGGCTACACACGTGCTACAATGGTCGGTACAGAGGGTTG

CCAAGCCGCGAGGTGGAGCTAATCCCACAAAACCGATCGTAGTCCGGATCGCAGTCTGCA

ACTCGACTGCGTGAAGTCGGAATCGCTAGTAATCGCGAATCAGAATGTCGCGGTGAATAC

GTTCCCGGGCCTTGTACACACCGCCCGTCACACCATGGGAGTGGGTTGCACCAGAAGTAG

CTAGTCTAACCTTCGGGAGGACGGTA

>A12_Stenotrophomonas_sp.

GGGTGGCTAACACATGCAAGTCGAACGGCAGCACAGAGGAGCTTGCTCCTTGGGTGGCGA

GTGGCGGACGGGTGAGGAATACATCGGAATCTACTTTTTCGTGGGGGATAACGTAGGGAA

ACTTACGCTAATACCGCATACGACCTACGGGTGAAAGCAGGGGACCTTCGGGCCTTGCGC

GATTGAATGAGCCGATGTCGGATTAGCTAGTTGGCGGGGTAAAGGCCCACCAAGGCGACG

ATCCGTAGCTGGTCTGAGAGGATGATCAGCCACACTGGAACTGAGACACGGTCCAGACTC

CTACGGGAGGCAGCAGTGGGGAATATTGGACAATGGGCGCAAGCCTGATCCCAGCCATAC

CGCGTGGGTGAAGAAGGCCTTCGGGTTGTAAAGCCCTTTTGTTGGGAAAGAAATCCAGCC

GGGCTAATACCTGGTTGGGATGACGGTACCCAAAGAATAAGCACCGGCTAACTTCGTGCC

AGCAGCCGCGGTAATACGAAGGGTGCAAGCGTTACTCGGAATTACTGGGCGTAAAGCGTG

CGTAGGTGGTTGTTTAAGTCTGTTGTGAAAGCCCTGGGCTCAACCTGGGAACTGCAGTGG

AAACTGGACAACTAGAGTGTGGTAGAGGGTAGCGGAATTCCCGGTGTAGCAGTGAAATGC

GTAGAGATCGGGAGGAACATCCATGGCGAAGGCAGCTACCTGGACCAACACTGACACTGA

GGCACGAAAGCGTGGGGAGCAAACAGGATTAGATACCCTGGTAGTCCACGCCCTAAACGA

TGCGAACTGGATGTTGGGTGCAATTTGGCACGCAGTATCGAAGCTAACGCGTTAAGTTCG

CCGCCTGGGGAGTACGGTCGCAAGACTGAAACTCAAAGGAATTGACGGGGGCCCGCACAA

GCGGTGGAAGTATGTGGTTTAATTCGATGCAACGCGAAGAACCTTACCTGGCCTTGACAT

GTCGAGAACTTTCCAGAAGATGGATTGGTGCCTTCGGGAACTCGAACACAGGTGCTGCAT

GGCTGTCGTCAGCTCGTGTCGTGAGATGTTTGGGTTAAGTCCCGCAACGAGCGCAACCCT

TGTCCTTAGTTGCCAGCACGTAATGGTGGGAACTCTAAGGAGACCGCCGGTGACAAACCG

GAGAAAAGGGTGGGAATAACGTCAAGTCATCATGGCCCTTACGGCCAGGGCTACACACGT

ACTACAATGGTAGGGACAAAGGGCTGCAAGCCGGCGACGGTAAGCCAATCCCAGAAACCC

TATCTCAGTCCGGATTGGAGTCTGCAACTCGACTCCATGAAGTCGGAATCGCTAGTAATC

GCAGATCAGCATTGCTGCGGTGAATACGTTCCCGGGCCTTGTACACACCGCCCGTCACAC

CATGGGAGTTTGTTGCACCAGAAGCAGGTAGCTTAACCTTCGGGAGGGCGCTG

>B1_Variovorax_sp._forward

MMNNNTGCANGTCGAACGGCAGCGCGGGAGCAATCCTGGCGGCGNGTGGCGAACGGGTGAGTAATACATCGGAACGTGCC

CAATCGTGGGGGATAACGCAGCGNNRGCTGTGCTAATACCGCNNNCGATCTACGGATGAAAGCAGGGGATCGCAAGACCT

TGCGCGAATGGAGCGGCCGATGGCAGATTAGGTAGTTGGTGAGGTAAAGGCTCACCAAGCCTTCGATCTGTAGCTGGTCT

GAGAGGACGACCAGCCACACTGGGACTGAGACACGGCCCAGACTCCTACGGGAGGCAGCAGTGGGGAATTTTGGACAATG

GGCGAAAGCCTGATCCAGCCATGCCGCGTGCAGGAWGAAGGCCTTCGGGTTGTAAACTGCTTTTGTACGGAACGAAACGG

CCTTTTCTAATAAAGAAGGCTAATGACGGTACCGTAAGAATAAGCACCGGCTAACTACGTGCCAGCAGCCGCGGTAATAC

GTAGGGTGCAAGCGTTAATCGGAATTACTGGGCGTAAAGCGTGCGCAGGCGGTTATGTAAGACAGTTGTGAAATCCCCGG

GCTCAACCTGGGAACTGCATCTGTGACTGCATAGCNNNAGTACGGTAGAGGGGGATGGAATTCCGCGTGTAGCNNNGAAA

TGCGTAGANNNGCGGAGGAACACCGANNNCGANNNCAATCCCCTGGACCTGTACTGACGCTCATGCACGAAAGCGTGGGG

AGCAAACAGGATTAGATACCCTGGTAGTCCACGCCCTAAACGATGTCAACTGGTTGTTGGGTCTTCACTGACTCAGTAAC

GAAGNNNACGCGTGAAGTTGACCGCCTGGGGAGTACGGCCGCAAGGTTGAAACTCAAAGGAATTGACGGGGACCCGCACA

AGCGGTGGANGATGTGGTTTAATTCGATGCAACGCGAAAAACCTTACCCACCTTTGACATGTACGGAATTCGCCANAGAT

GGCTTANTGCTCGAAAGAGAACCGTAACACAGGTGCTGCAGGGCTGTCGTCAGCTCGTGTCGTGAGATGTTGGGTTAAGT

CCCGCNNCGAGCGCANNCTTGTNNTAGTTGCTANNNTCAGTTGGGCACTCTNNGANACTGCCNNNGN

>B2_Pseudomonas_sp.

TTGCTTCGAATTAAACCACATGCTCCACCGCTTGTGCGGGCCCCCGTCAATTCATTTGAGTTTTAACCTT

GCGGCCGTACTCCCCAGGCGGTCAACTTAATGCGTTAGCTGCGCCACTAAAAGCTCAAGGCTTCCAACGG

CTAGTTGACATCGTTTACGGCGTGGACTACCAGGGTATCTAATCCTGTTTGCTCCCCACGCTTTCGCACC

TCAGTGTCAGTATTAGTCCAGGTGGTCGCCTTCGCCACTGGTGTTCCTTCCTATATCTACGCATTTCACC

GCTACACAGGAAATTCCACCACCCTCTACCATACTCTAGTCAGTCAGTTTTGAATGCAGTTCCCAGGTTG

AGCCCGGGGATTTCACATCCAACTTAACAAACCACCTACGCGCGCTTTACGCCCAGTAATTCCGATTAAC

GCTTGCACCCTCTGTATTACCGCGGCTGCTGGCACAGAGTTAGCCGGTGCTTATTCTGTCGGTAACGTCA

AAACACTAACGTATTAGGTTAATGCCCTTCCTCCCAACTTAAAGTGCTTTACAATCCGAAGACCTTCTTC

ACACACGCGGCATGGCTGGATCAGGCTTTCGCCCATTGTCCAATATTCCCCACTGCTGCCTCCCGTAGGA

GTCTGGACCGTGTCTCAGTTC

>B3_Pseudomonas_sp.

TGCAAGTCGAGCGGTAGAGAGAAGCTTGCTTCTCTTGAGAGCGGCGGACGGGTGAGTAAT

GCCTAGGAATCTGCCTGGTAGTGGGGGATAACGCTCGGAAACGGACGCTAATACCGCATA

CGTCCTACGGGAGAAAGCAGGGGACCTTCGGGCCTTGCGCTATCAGATGAGCCTAGGTCG

GATTAGCTAGTTGGTGAGGTAATGGCTCACCAAGGCGACGATCCGTAACTGGTCTGAGAG

GATGATCAGTCACACTGGAACTGAGACACGGTCCAGACTCCTACGGGAGGCAGCAGTGGG

GAATATTGGACAATGGGCGAAAGCCTGATCCAGCCATGCCGCGTGTGTGAAGAAGGTCTT

CGGATTGTAAAGCACTTTAAGTTGGGAGGAAGGGCATTAACCTAATACGTTGGTGTCTTG

ACGTTACCGACAGAATAAGCACCGGCTAACTCTGTGCCAGCAGCCGCGGTAATACAGAGG

GTGCAAGCGTTAATCGGAATTACTGGGCGTAAAGCGCGCGTAGGTGGTTCGTTAAGTTGG

ATGTGAAATCCCCGGGCTCAACCTGGGAACTGCATTCAAAACTGACGAGCTAGAGTATGG

TAGAGGGTGGGTGGAATTTCCTGTGTAGCGGTGAAATGCGTAGATATAGGAAGGAACACC

AGTGGCGAAGGCGACCACCTGGACTGATACTGACACTGAGGTGCGAAAGCGTGGGGAGCA

AACAGGATTAGATACCCTGGTAGTCCACGCCGTAAACGATGTCAACTAGCCGTTGGGAGC

CTTGAGCTCTTAGTGGCGCAGCTAACGCATTAAGTTGACCGCCTGGGGAGTACGGCCGCA

AGGTTAAAACTCAAATGAATTGACGGGGGCCCGCACAAGCGGTGGAGCATGTGGTTTAAT

TCGAAGCAACGCGAAGAACCTTACCAGGCCTTGACATCCAATGAACTTTCCAGAGAATGG

ATTGGTGCCTTCGGGAACATTGAGACAGGTGCTGCATGGCTGTCGTCAGCTCGTGTCGTG

AGATGTTTGGGTTAAGTCCCGTAACGAGCGCAACCCTTGTCCTTAGTTACCAGCACGTAA

TGGTGGGCACTCTAAGGAGACTGCCGGTGACAAACCGGAGGAAGGTGGGGATGACGTCAA

GTCATCATGGCCCTTACGGCCTGGGCTACACACGTGCTACAATGGTCGGTACAGAGGGTT

GCCAAGCCGCGAGGTGGAGCTAATCCCAGAAAACCGATCGTAGTCCGGATCGCAGTCTGC

AACTCGACTGCGTGAAGTCGGAATCGCTAGTAATCGCGAATCAGAATGTCGCGGTGAATA

CGTTCCCGGGCCTTGTACACACCGCCCGTCACACCATGGGAGTGGGTTGCACCAGAAGTA

GCTAGTCTAACCTTCGGGAGGACGGTA

>B4_Brevundimonas_sp.

TGCAAGTCGAACGAACTCTTCGGAGTTAGTGGCGGAGGGTGAGTAACACGTGGGAACGTG

CCTTTTGGTTCGGAATAACTCAGGGAAACTTGTGCTAATACCGAATGTGCCCTTCGGGGG

AAAGATTTATCGCCATTAGAGCGGCCCGCGTCTGATTAGCTAGTTGGTGAGGTAAAGGCT

CACCAAGGCGACGATCAGTAGCTGGTCTGAGAGGATGATCAGCCACATTGGGACTGAGAC

ACGGCCCAAACTCCTACGGGAGGCAGCAGTGGGGAATCCTGCGCAATGGGCGAAAGCCTG

ACGCAGCCATGCCGCGTGAATGATGAAGGTCTTAGGATTGTAAAATTCTTTCACCGGGGA

CGATAATGACGGTACCCGGAGAAGAAGCCCCGGCCAAATTCGTGCCAGCAGCCGCGGTAA

TACGAAGGGGGCCAGCGTTGCTCGGAATTACTGGGCGTAAAGGGAGCGTAGGCGGACATT

TAAGTCAGGGGTGAAATCCCGGGGCTCAACCTCGGAATTGCCTTTGATACTGGGTGTCTT

GAGTATGAGAGAGGTATGTGGAACTCCGAGTGTAGAGGTGAAATTCGTAGATATTCGGAA

GAACACCAGTGGCGAAGGCGACATACTGGCTCATTACTGACGCTGAGGCTCGAAAGCGTG

GGGAGCAAACAGGATTAGATACCCTGGTAGTCCACGCCGTAAACGATGATTGCTAGTTGT

CGGGATGCATGCATTTCGGTGACGCAGCTAACGCATTAAGCAATCCGCCTGGGGAGTACG

GTCGCAAGATTAAAACTCAAAGGAATTGACGGGGGCCCGCACAAGCGGTGGAGCATGTGG

TTTAATTCGAAGCAACGCGCAGAACCTTACCACCTTTTGACATGCCTGGACCGCCAGAGA

GATCTGGCTTTCTCTTCGGAGACTAGGACACAGGTGCTGCATGGCTGTCGTCAGCTCGTG

TCGTGAGATGTTGGGTTAAGTCCCGCAACGAGCGCAACCCTCGCCATTAGTTGCCATCAT

TTAGTTGGGAACTCTAATGGGACTGCCGGTGCTAACCCGGAGGAAGGGTGGGGATGACGT

CAAGTCCTCAGGGCCCTTACAGGGTGGGCTACACACGTGCTACAATGGCGACTACAGAGG

GTTAATCCTTAAAAGTCGTCTCAGTTCGGATTGTCCTCTGCAACTCGAGGGCATGAAGTT

GGAATCGCTAGTAATCGCGGATCAGCATGCCGCGGTGAATACGTTCCCGGGCCTGTACAC

ACCGCCCGTCACACCATGGGAGTTGTTCTACCCGAAGGCGCTGCGCTGACCGCAAGGGGC

AGGGCAAACCCCCCG

>B5_Streptomyces_sp.

CTTTACCATGCAAGTCGAACGATGAAGCCCTTCGGGGTGGATTAGTGGCGAACGGGTGAG

TAACACGTGGGCAATCTGCCCTTCACTCTGGGACAAGCCCTGGAAACGGGGTCTAATACC

GGATAACACTCTGTCCCGCATGGGACGGGGTTAAAAGCTCCGGCGGTGAAGGATGAGCCC

GCGGCCTATCAGCTTGTTGGTGGGGTAATGGCCTACCAAGGCGACGACGGGTAGCCGGCC

TGAGAGGGCGACCGGCCACACTGGGACTGAGACACGGCCCAGACTCCTACGGGAGGCAGC

AGTGGGGAATATTGCACAATGGGCGAAAGCCTGATGCAGCGACGCCGCCTGAGGGATGAC

GGCCTTCGGGTTGTAAACCTCTTTCAGCAGGGAAGAAGCGAAAGTGACGGTACCTGCAGA

AGAAGCGCCGGCTAACTACGTGCCAGCAGCCGCGGTAATACGTAGGGCGCAAGCGTTGTC

CGGAATTATTGGGCGTAAAGAGCTCGTAGGCGGCTTGTCACGTCGGATGTGAAAGCCCGG

GGCTTAACCCCGGGTCTGCATTCGATACGGGCTAGCTAGAGTGTGGTAGGGGAGATCGGA

ATTCCTGGTGTAGCGGTGAAATGCGCAGATATCAGGAGGAACACCGGTGGCGAAGGCGGA

TCTCTGGGCCATTACTGACGCTGAGGAGCGAAAGCGTGGGGAGCGAACAGGATTAGATAC

CCTGGTAGTCCACGCCGTAAACGTTGGGAACTAGGTGTTGGCGACATTCCACGTCGTCGG

TGCCGCAGCTAACGCATTAAGTTCCCCGCCTGGGGAGTACGGCCGCAAGGCTAAAACTCA

AAGGAATTGACGGGGGCCCGCACAAGCAGCGGAGCATGTGGCTTAATTCGACGCAACGCG

AAGAACCTTACCAAGGCTTGACATATACCGGAAAGCATCAGAGATGGTGCCCCCCTTGTG

GTCGGTATACAGGTGGTGCATGGCTGTCGTCAGCTCGTGTCGTGAGATGTTGGGTTAAGT

CCCGCAACGAGCGCAACCCTTGTTCTGTGTTGCCAGCATGCCCTTCGGGGTGATGGGGAC

TCACAGGAGACTGCCGGGGTCAACTCGGAGGAAGGTGGGGACGACGTCAAGTCATCATGC

CCCTTATGTCTTGGGCTGCACACGTGCTACAATGGCCGGTACAATGAGCTGCAATGCCCC

GAGGCGGAACGAATCTCAAAAAGCCGGTCTCAGTTCGGATTGGGGTCTGCAACTCGACCC

CATGAAGTCGGAGTTGCTAGTAATCGCAGATCAGCATTGCTGCGGTGAATACGTTCCCGG

GCCTTGTACACACCGCCCGTCACGTCACGAAAGTCGGTAACACCCGAAGCCGGTGGCCCA

ACCCCTTGTGGAGGGAGCGTCGAAG

>B6_Agrobacterium_tumefaciens

GGTTAGCTGCCTCCTTGCGGTTAGCGCACTACCTTCGGGTAAACCAACTCCCATGGTGTG

ACGGGCGGTGTGTACAAGGCCCGGGAACGTATTCACCGCAGCATGCTGATCTGCGATTAC

TAGCGATTCCAACTTCATGCACTCGAGTTGCAGAGTGCAATCCGAACTGAGATGGCTTTT

GGAGATTAGCTCGACATCGCTGTCTCGCTGCCCACTGTCACCACCATTGTAGCACGTGTG

TAGCCCAGCCCGTAAGGGCCATGAGGACTTGACGTCATCCCCACCTTCCTCTCGGCTTAT

CACCGGCAGTCCCCTTAGAGTGCCCAACTAAATGCTGGCAACTAAGGGCGAGGGTTGCGC

TCGTTGCGGGACTTAACCCAACATCTCACGACACGAGCTGACGACAGCCATGCAGCACCT

GTTCTGGGGCCAGCCTAACTGAAGGACAATGTCTCCACTGCCCAAACCCCGAATGTCAAG

AGCTGGTAAGGTTCTGCGCGTTGCTTCGAATTAAACCACATGCTCCACCGCTTGTGCGGG

CCCCCGTCAATTCCTTTGAGTTTTAATCTTGCGACCGTACTCCCCAGGCGGAATGTTTAA

TGCGTTAGCTGCGCCACCGAACAGTATACTGCCCGACGGCTAACATTCATCGTTTACGGC

GTGGACTACCAGGGTATCTAATCCTGTTTGCTCCCCACGCTTTCGCACCTCAGCGTCAGT

AATGGACCAGTAAGCCGCCTTCGCCACTGGTGTTCCTCCGAATATCTACAAATTTCACCT

CTACACTCGGAATTCCACTTACCTCTTCCATACTCAAGATACCCAGTATCAAAGGCAGTT

CCAGAGTTGAGCTCTGGGATTTCACCCCTGACTTAAATATCCGCCTACGTGCGCTTTACG

CCCAGTAATTCCGAACAACGCTAGCCCCCTTCGTATTACCGCGGCTGCTGGCACGAAGTT

AGCCGGGGCTTCTTCTCCGGATACCGTCATTATCTTCTCCGGTGAAAGAGCTTTACAACC

CTAAGGCCTTCATCACTCACGCGGCATGGCTGGATCAGGCTTGCGCCCATTGTCCAATAT

TCCCCACTGCTGCCTCCCGTAGGAGTTTGGGCCGTGTCTCAGTCCCAATGTGGCTGATCA

TCCTCTCAGACCAGCTATGGATCGTCGCCTTGGTAGGCCTTTACCCCACCAACTAGCTAA

TCCAACGCGGGCTCATCATACCCCGATAAATCTTTCCCCCGTAGGGCGTATGCGGTATTA

ATTCCAGTTTCCCGGAGCTATTCCGCAGGGCACGGTAGATTCCCACGCGTTACTCACCCG

TCTGCCACTCCCCATGCGGGCGTTCGA

>B7_Streptomyces_sp.

TGCAAGTCGAACGATGAAGCCCTTCGGGGTGGATTAGTGCGAACGGGTGAGTAACACGTG

GGCAATCTGCCCTTCACTCTGGGACAAGCCCTGGAAACGGGGTCTAATACCGGATAACAC

TCTGTCCCGCATGGGACGGGGTTAAAAGCTCCGGCGGTGAAGGATGAGCCCGCGGCCTAT

CAGCTTGTTGGTGGGGTAATGGCCTACCAAGGCGACGACGGGTAGCCGGCCTGAGAGGGC

GACCGGCCACACTGGGACTGAGACACGGCCCAGACTCCTACGGGAGGCAGCAGTGGGGAA

TATTGCACAATGGGCGAAAGCCTGATGCAGCGACGCCGCCTGAGGGATGACGGCCTTCGG

GTTGTAAACCTCTTTCAGCAGGGAAGAAGCGAAAGTGACGGTACCTGCAGAAGAAGCGCC

GGCTAACTACGTGCCAGCAGCCGCGGTAATACGTAGGGCGCAAGCGTTGTCCGGAATTAT

TGGGCGTAAAGAGCTCGTAGGCGGCTTGTCACGTCGGATGTGAAAGCCCGGGGCTTAACC

CCGGGTCTGCATTCGATACGGGCTAGCTAGAGTGTGGTAGGGGAGATCGGAATTCCTGGT

GTAGCGGTGAAATGCGCAGATATCAGGAGGAACACCGGTGGCGAAGGCGGATCTCTGGGC

CATTACTGACGCTGAGGAGCGAAAGCGTGGGGAGCGAACAGGATTAGATACCCTGGTAGT

CCACGCCGTAAACGTTGGGAACTAGGTGTTGGCGACATTCCACGTCGTCGGTGCCGCAGC

TAACGCATTAAGTTCCCCGCCTGGGGAGTACGGCCGCAAGGCTAAAACTCAAAGGAATTG

ACGGGGGCCCGCACAAGCAGCGGAGCATGTGGCTTAATTCGACGCAACGCGAAGAACCTT

ACCAAGGCTTGACATATACCGGAAAGCATCAGAGATGGTGCCCCCCTTGTGGTCGGTATA

CAGGTGGTGCATGGCTGTCGTCAGCTCGTGTCGTGAGATGTTGGGTTAAGTCCCGCAACG

AGCGCAACCCTTGTTCTGTGTTGCCAGCATGCCCTTCGGGGTGATGGGGACTCACAGGAG

ACTGCCGGGGTCAACTCGGAGGAAGGTGGGGACGACGTCAAGTCATCATGCCCCTTATGT

CTTGGGCTGCACACGTGCTACAATGGCCGGTACAATGAGCTGCGATGCCGCGAGGCGGAG

CGAATCTCAAAAAGCCGGTCTCAGTTCGGATTGGGGTCTGCAACTCGACCCCATGAAGTC

GGAGTTGCTAGTAATCGCAGATCAGCATTGCTGCGGTGAATACGTTCCCGGGCCTTGTAC

ACACCGCCCGTCACGTCACGAAAGTCGGTAACACCCGAAGCCGGTGGCCCAACCCCTTGT

GGGAGGGAGC

>B8_Variovorax_boronicumulans

ATCGCCCTCCTTGCGGTTAAGCTAACTACTTCTGGCAGAACCCGCTCCCATGGTGTGACG

GGCGGTGTGTACAAGACCCGGGAACGTATTCACCGTGACATTCTGATCCACGATTACTAG

CGATTCCGACTTCACGCAGTCGAGTTGCAGACTGCGATCCGGACTACGACTGGTTTTATG

GGATTAGCTCCCCCTCGCGGGTTGGCAACCCTTTGTACCAGCCATTGTATGACGTGTGTA

GCCCCACCTATAAGGGCCATGAGGACTTGACGTCATCCCCACCTTCCTCCGGTTTGTCAC

CGGCAGTCTCATTAGAGTGCCCAACTGAATGTAGCAACTAATGACAAGGGTTGCGCTCGT

TGCGGGACTTAACCCAACATCTCACGACACGAGCTGACGACAGCCATGCAGCACCTGTGT

TACGGTTCTCTTTCGAGCACTAAGCCATCTCTGGCGAATTCCGTACATGTCAAAGGTGGG

TAAGGTTTTTCGCGTTGCATCGAATTAAACCACATCATCCACCGCTTGTGCGAGTCCCCG

TCAATTCCTTTGAGTTTCAACCTTGCGGCCGTACTCCCCAGGCGGTCAACTTCACGCGTT

AGCTTCGTTACTGAGTCAGTGAAGACCCAACAACCAGTTGACATCGTTTAGGGCGTGGAC

TACCAGGGTATCTAATCCTGTTTGCTCCCCACGCTTTCGTGCATGAGCGTCAGTACAGGT

CCAGGGGATTGCCTTCGCCATCGGTGTTCCTCCGCATATCTACGCATTTCACTGCTACAC

GCGGAATTCCATCCCCCTCTACCGTACTCTAGCTATGCAGTCACAGATGCAGTTCCCAGG

TTGAGCCCGGGGATTTCACAACTGTCTTACATAACCGCCTGCGCACGCTTTACGCCCAGT

AATTCCGATTAACGCTTGCACCCTACGTATTACCGCGGCTGCTGGCACGTAGTTAGCCGG

TGCTTATTCTTACGGTACCGTCATTAGCCTTCTGTATTAGAAAAGACCGTTTCGTTCCGT

ACAAAAGCAGTTTACAACCCGAAGGCCTTCATCCTGCACGCGGCATGGCTGGATCAGGCT

TTCGCCCATTGTCCAAAATTCCCCACTGCTGCCTCCCGTAGGAGTCTGGGCCGTGTCTCA

GTCCCAGTGTGGCTGGTCGTCCTCTCAGACCAGCTACAGATCGAAGGCTTGGTGAGCCTT

TACCTCACCAACTACCTAATCTGCCATCGGCCGCTCCATTCGCGCAAGGTCTTGCGATCC

CCTGCTTTCATCCGTAGATCGTATGCGGTATTAGCACAGCTTTCGCTGCGTTATCCCCCA

CGATTGGGCACGTT

>B9_Microbacterium_sp._forward

KCTTNNNMNTGCAAGTCGAACGGTGAAGCCCAGCTTGCTGGGTGGATCAGTGGCGAACGGGTGAGTAACACGTGAGCAAC

CTGCCCCTGACTCTGGGATAAGCGCTGGAAACGGCGTCTAATACTGGATACGAGTAGCGACCGCATGGTCAGTTACTGGA

AAGATTTATTGGTTGGGGATGGGCTCGCGGCCTATCAGCTTGTTGGTGAGGTAATGGCTCACCAAGGCGTCGACGGGTAG

CCGGCCTGAGAGGGTGACCGGCCACACTGGGACTGAGACACGGCCCAGACTCCTACGGGAGGCAGCAGTGGGGAATATTG

CACAATGGGCGCAAGCCTGATGCAGCAACGCCGCGTGAGGGATGACGGCCTTCGGGTTGTAAACCTCTTTTAGCAGGGAA

GAAGCGAAAGTGACGGTACCTGCAGAAAAAGCGCCGGCTAACTACGTGCCAGCAGCCGCGGTAATACGTAGGGCGCAAGC

GTTATCCGGAATTATTGGGCGTAAAGAGCTCGTAGGCGGTTTGTCGCGTCTGCTGTGAAATCCGGAGGCTCAACCTCCGG

CCTGCAGTGGGTACGGGCAGACTAGAGTGCGGTAGGGGAGATTGGAATTCCTGGTGTAGCGGTGGAATGCGCAGATATCA

GGAGGAACACCGATGGCGAAGGCAGATCTCTGGGCCGTAACTGACGCTGAGGAGCGAAAGGGTGGGGAGCAAACAGGCTT

AGATACCCTGGTAGTCCACCCCGTAAACGTTGGGAACTAGTTGTGGGGTCCATTCCACGGATTCCGTGACGCAGCTAACG

CATTAAGTTCCCCGCCTGGGGAGTACGGCCGCAAGGCTAAAACTCAAAGGAATTGACGGGGACCCGCACAAGCGGCGGAG

CATGCGGATTAATTCGATGCAACGCGAAGAACCTTACCAAGGCTTGACATATACGAGAACGGGCCAGAAATGGTCAACTC

TTTGGACACTCGTAAACAGGTGGTGCATGGTTGTCGTCAGCTCGTGTCGTGAGATGTTGGGTTAAGTCCCGCAACGAGCG

CAACCCTCGTTCTATGTTGCCAGCACGTAATGGTGGGAACTCATGGGANACTGNCGGGGTCNNTCNG

>B10_Pseudomonas_fluorescens

GGGCGGCCTAACACATGCAAGTCGAGCGGATGAGAGGAGCTTGCTCCTGGATTCAGCGGC

GGACGGGTGAGTAATGCCTAGGAATCTGCCTGGTAGTGGGGGACAACGTTTCGAAAGGAA

CGCTAATACCGCATACGTCCTACGGGAGAAAGCAGGGGACCTTCGGGCCTTGCGCTATCA

GATGAGCCTAGGTCGGATTAGCTAGTTGGTGAGGTAATGGCTCACCAAGGCGACGATCCG

TAACTGGTCTGAGAGGATGATCAGTCACACTGGAACTGAGACACGGTCCAGACTCCTACG

GGAGGCAGCAGTGGGGAATATTGGACAATGGGCGAAAGCCTGATCCAGCCATGCCGCGTG

TGTGAAGAAGGTCTTCGGATTGTAAAGCACTTTAAGTTGGGAGGAAGGGCAGTAACTTAA

TACGTTGCTGTTTTGACGTTACCGACAGAATAAGCACCGGCTAACTCTGTGCCAGCAGCC

GCGGTAATACAGAGGGTGCAAGCGTTAATCGGAATTACTGGGCGTAAAGCGCGCGTAGGT

GGTTCGTTAAGTTGGATGTGAAATCCCCGGGCTCAACCTGGGAACTGCATTCAAAACTGT

CGAGCTAGAGTATGGTAGAGGGTGGTGGAATTTCCTGTGTAGCGGTGAAATGCGTAGATA

TAGGAAGGAACACCAGTGGCGAAGGCGACCACCTGGACTGATACTGACACTGAGGTGCGA

AAGCGTGGGGAGCAAACAGGATTAGATACCCTGGTAGTCCACGCCGTAAACGATGTCAAC

TAGCCGTTGGGAGCCTTGAGCTCTTAGTGGCGCAGCTAACGCATTAAGTTGACCGCCTGG

GGAGTACGGCCGCAAGGTTAAAACTCAAATGAATTGACGGGGGCCCGCACAAGCGGTGGA

GCATGTGGTTTAATTCGAAGCAACGCGAAGAACCTTACCAGGCCTTGACATCCAATGAAC

TTTCCAGAGATGGATTGGTGCCTTCGGGAACATTGAGACAGGTGCTGCATGGCTGTCGTC

AGCTCGTGTCGTGAGATGTTTGGGTTAAGTCCCGTAACGAGCGCAACCCTTGTCCTTAGT

TACCAGCACGTTATGGTGGGCACTCTAAGGAGACTGCCGGTGACAAACCGAGAAGAAGGT

GGGGGATGAACTCAAGTCATCATGGCCCTTACGGCCTGGGCTACACACGTGCTACAATGG

TCGGTACAAAGGGTTGCCAAGCCGCGAGGTGGAACTAATCCCATAAAACCGATCGTAGTC

CGGATCGCAGTCTGCAACTCGACTGCGTGAAGTCGGAATCGCTAGTAATCGTGAATCAGA

ATGTCACGGTGAATACGTTCCCGGGCCTTGTACACACCGCCCGTCACACCATGGGAGTGG

GTTGCACCAGAAGTAGCTAGTCTAACCTTCGGGAGGACGGTACC

>B11_Variovorax_boronicumulans

CCCGCTCCCATGGTGTGACGGGCGGTGTGTACAAGACCCGGGAACGTATTCACCGTGACA

TTCTGATCCACGATTACTAGCGATTCCGACTTCACGCAGTCGAGTTGCAGACTGCGATCC

GGACTACGACTGGTTTTATGGGATTAGCTCCCCCTCGCGGGTTGGCAACCCTTTGTACCA

GCCATTGTATGACGTGTGTAGCCCCACCTATAAGGGCCATGAGGACTTGACGTCATCCCC

ACCTTCCTCCGGTTTGTCACCGGCAGTCTCATTAGAGTGCCCAACTGAATGTAGCAACTA

ATGACAAGGGTTGCGCTCGTTGCGGGACTTAACCCAACATTCTCACGACACGAGCTGACG

ACAGCCATGCAGCACCTGTGTTACGGTTCTCTTTCGAGCACTAAGCCATCTCTGGCGAAT

TCCGTACATGTCAAAGGTGGGTAAGGTTTTTCGCGTTGCATCGAATTAAACCAACATTCA

TCCACCGCTTGTGCGGGTCCCCGTCAATTCCTTTGAGTTTCAACCTTGCGGCCGTACTCC

CCAGGCGGTCAACTTCACGCGTTAGCTTCGTTACTGAGTCAGTGAAGACCCAACAACCAG

TTGACATCGTTTAGGGCGTGGACTACCAGGGTATCTAATCCTGTTTGCTCCCCACGCTTT

CGTGCATGAGCGTCAGTACAGGTCCAGGGGATTGCCTTCGCCATCGGTGTTCCTCCGCAT

ATCTACGCATTTCACTGCTACACGCGGAATTCCATCCCCCTCTACCGTACTCTAGCTATG

CAGTCACAGATGCAGTTCCCAGGTTGAGCCCCGGGGATTTCACAACTGTCTTACATAACC

GCCTGCGCACGCTTTACGCCCAGTAATTCCGATTAACGCTTGCACCCTACGTATTACCGC

GGCTGCTGGCACGTAGTTAGCCGGTGCTTATTCTTACGGTACCGTCATTAGCCTTCTGTA

TTAGAAAAGACCGTTTCGTTCCGTACAAAAGCAGTTTACAACCCGAAGGCCTTCATCCTG

CACGCGGCAGGGCTGGATCAGGCTTTCGCCCATTGTCCAAAATTCCCCACTGCTGCCTCC

CGTAGGAGTCTGGGCCGTGTCTCAGTCCCAGTGTGGCTGGTCGTCCTCTCAGACCAGCTA

CAGATCGAAGGCTTGGTGAGCCTTTACCTCACCAACTACCTAATCTGCCATCGGCCGCTC

CATTCGCGCAAGGTCTTGCGATCCCCTGCTTTCATCCGTAGATCGTATGCGGTATTAGCA

CAGCTTTCGCTGCGTTATCCCCCACGATTGGGCACGTTCCGATGTATTACTCACCCGTTC

GCCACTCGCCGCCAGGATTGCTCCCGCGCTGCCGTTCGACTTGCA

>B12_Bacillus_Peribacillus_sp.

CTGCAAGTCGAGCGAATCGATGGGAGCTTGCTCCCTGAGATTAGCGGCGGACGGGTGAGT

AACACGTGGGCAACCTGCCTATAAGACTGGGATAACTTCGGGAAACCGGAGCTAATACCG

GATATGTTCTTTTCTCGCATGAGAGAAGATGGAAAGACGGTTTCGGCTGTCACTTATAGA

TGGGCCCGCGGCGCATTAGCTAGTTGGTGAGGTAATGGCTCACCAAGGCGACGATGCGTA

GCCGACCTGAGAGGGTGATCGGCCACACTGGGACTGAGACACGGCCCAGACTCCTACGGG

AGGCAGCAGTAGGGAATCTTCCGCAATGGACGAAAGTCTGACGGAGCAACGCCGCGTGAA

CGAAGAAGGCCTTCGGGTCGTAAAGTTCTGTTGTTAGGGAAGAACAAGTACCAGAGTAAC

TGCTGGTACCTTGACGGTACCTAACCAGAAAGCCACGGCTAACTACGTGCCAGCAGCCGC

GGTAATACGTAGGTGGCAAGCGTTGTCCGGAATTATTGGGCGTAAAGCGCGCGCAGGTGG

TTCTTTAAGTCTGATGTGAAAGCCCACGGCTCAACCGTGGAGGGTCATTGGAAACTGGGG

AACTTGAGTGCAGAAGAGGAAAGTGGAATTCCAAGTGTAGCGGTGAAATGCGTAGAGATT

TGGAGGAACACCAGTGGCGAAGGCGACTTTCTGGTCTGTAACTGACACTGAGGCGCGAAA

GCGTGGGGAGCAAACAGGATTAGATACCCTGGTAGTCCACGCCGTAAACGATGAGTGCTA

AGTGTTAGAGGGTTTCCGCCCTTTAGTGCTGCAGCTAACGCATTAAGCACTCCGCCTGGG

GAGTACGACCGCAAGGTTGAAACTCAAAGGAATTGACGGGGGCCCGCACAAGCGGTGGAG

CATGTGGTTTAATTCGAAGCAACGCGAAGAACCTTACCAGGTCTTGACATCCTCTGACAC

TCCTAGAGATAGGACGTTCCCCTTCGGGGGACAGAGTGACAGGTGGGGCATGGTTGTCGT

CAGCTCGTGTCGTGAGATGTTGGGTTAAGTCCCGCAACGAGCGCAACCCTTGATCTTAGT

TGCCAGCATTCAGTTGGGCACTCTAAGGTGACTGCCGGTGACAAACCGGAGGAAGGTGGG

GATGACGTCAAATCATCATGCCCCTTATGACCTGGGCTACACACGTGCTACAATGGATGG

TACAAAGGGCTGCAAACCTGCGAAGGTAAGCGAATCCCATAAAGCCATTCTCAGTTCGGA

TTGTAGGCTGCAACTCGCCTGCATGAAGCCGGAATCGCTAGTAATCGCGGATCAGCATGC

CGCGGTGAATACGTTCCCGGGCCTTGTACACACCGCCCGTCACACCACGAGAGTTTGTAA

CACCCGAAGTCGGTGAGGTAACCTTTATGGAGCCAGCCCCTA

>C1_Pseudomonas_sp.

ATGCAAGTCGAGCGGTAGAGAGAAGCTTGCTTCTCTTGAGAGCGGCGGACGGGTGAGTAA

TGCCTAGGAATCTGCCTGGTAGTGGGGGATAACGTTCGGAAACGGACGCTAATACCGCAT

ACGTCCTACGGGAGAAAGCAGGGGACCTTCGGGCCTTGCGCTATCAGATGAGCCTAGGTC

GGATTAGCTAGTTGGTGAGGTAATGGCTCACCAAGGCGACGATCCGTAACTGGTCTGAGA

GGATGATCAGTCACACTGGAACTGAGACACGGTCCAGACTCCTACGGGAGGCAGCAGTGG

GGAATATTGGACAATGGGCGAAAGCCTGATCCAGCCATGCCGCGTGTGTGAAGAAGGTCT

TCGGATTGTAAAGCACTTTAAGTTGGGAGGAAGGGCAGTAAATTAATACTTTGCTGTTTT

GACGTTACCGACAGAATAAGCACCGGCTAACTCTGTGCCAGCAGCCGCGGTAATACAGAG

GGTGCAAGCGTTAATCGGAATTACTGGGCGTAAAGCGCGCGTAGGTGGTTTGTTAAGTTG

GATGTGAAATCCCCGGGCTCAACCTGGGAACTGCATTCAAAACTGACTGACTAGAGTATG

GTAGAGGGTGGGTGGAATTTCCTGTGTAGCGGTGAAATGCGTAGATATAGGAAGGAACAC

CAGTGGCGAAGGCGACCACCTGGACTAATACTGACACTGAGGTGCGAAAGCGTGGGGAGC

AAACAGGATTAGATACCCTGGTAGTCCACGCCGTAAACGATGTCAACTAGCCGTTGGAAG

CCTTGAGCTTTTAGTGGCGCAGCTAACGCATTAAGTTGACCGCCTGGGGAGTACGGCCGC

AAGGTTAAAACTCAAATGAATTGACGGGGGCCCGCACAAGCGGTGGAGCATGTGGTTTAA

TTCGAAGCAACGCGAAGAACCTTACCAGGCCTTGACATCCAATGAACTTTCTAGAGATAG

ATTGGTGCCTTCGGGAACATTGAGACAGGTGCTGCATGGCTGTCGTCAGCTCGTGTCGTG

AGATGTTGGGTTAAGTCCCGTAACGAGCGCAACCCTTGTCCTTAGTTACCAGCACGTCAT

GGTGGGCACTCTAAGGAGACTGCCGGTGACAAACCGGAGAAAGGGGGGGATGACGTCAAG

TCATCATGGCCCTTACGGCCTGGGCTACACACGTGCTACAATGGTCGGTACAGAGGGTTG

CCAAGCCGCGAGGTGGAGCTAATCCCACAAAACCGATCGTAGTCCGGATCGCAGTCTGCA

ACTCGACTGCGTGAAGTCGGAATCGCTAGTAATCGCGAATCAGAATGTCGCGGTGAATAC

GTTCCCGGGCCTTGTACACACCGCCCGTCACACCATGGGAGTGGGTTGCACCAGAAGTAG

CTAGTCTAACCTTCGGGAGGACGGTACC

>C2_Pseudomonas_brassicacearum

GCCTAACACATGCAAGTCGAGCGGTAGAGAGGTGCTTGCACCTCTTGAGAGCGGCGGACG

GGTGAGTAATGCCTAGGAATCTGCCTGGTAGTGGGGGATAACGCTCGGAAACGGACGCTA

ATACCGCATACGTCCTACGGGAGAAAGCAGGGGACCTTCGGGCCTTGCGCTATCAGATGA

GCCTAGGTCGGATTAGCTAGTTGGTGGGGTAATGGCTCACCAAGGCGGACGATCCGTAAC

TGGTCTGAGAGGATGATCAGTCACACCGGAACTGAGACACGGTCCAGACTCCTACGGGAG

GCAGCAGTGGGGAATATTGGACAATGGGCGAAAGCCTGATCCAGCCATGCCGCGTGTGTG

AAGAAGGTCTTCGGATTGTAAAGCACTTTAAGTTGGGAGGAAGGGCATTAACCTAATACG

TTAGTGTTTTGACGTTACCGACAGAATAAGCACCGGCTAACTCTGTGCCAGCAGCCGCGG

TAATACAGAGGGTGCAAGCGTTAATCGGAATTACTGGGCGTAAAGCGCGCGTAGGTGGTT

CGTTAAGTTGGATGTGAAATCCCCGGGCTCAACCTGGGAACTGCATTCAAAACTGTCGAG

CTAGAGTATGGTAGAGGGTGGTGGAATTTCCTGTGTAGCGGTGAAATGCGTAGATATAGG

AAGGAACACCAGTGGCGAAGGCGACCACCTGGACTGATACTGACACTGAGGTGCGAAAGC

GTGGGGAGCAAACAGGATTAGATACCCTGGTAGTCCACGCCGTAAACGATGTCAACTAGC

CGTTGGGAGCCTTGAGCTCTTAGTGGCGCAGCTAACGCATTAAGTTGACCGCCTGGGGAG

TACGGCCGCAAGGTTAAAACTCAAATGAATTGACGGGGGCCCGCACAAGCGGTGGAGCAT

GTGGTTTAATTCGAAGCAACGCGAAGAACCTTACCAGGCCTTGACATCCAATGAACTTTC

CAGAGATGGATTGGTGCCTTCGGGAACATTGAGACAGGTGCTGCATGGCTGTCGTCAGCT

CGTGTCGTGAGATGTTGGGTTAAGTCCCGTAACGAGCGCAACCCTTGTCCTTAGTTACCA

GCACGTTATGGTGGGCACTCTAAGGAGACTGCCGGTGACAAACCGGAGAAAGGGTGGGGA

TGACGTCAAGTCATCATGGCCCTTACGGCCTGGGCTACACACGTGCTACAATGGTCGGGT

ACAGAAGGTTGCCAAGCCGCGAGGTGGAGCTAATCCCACAAAACCGATCGTAGTCCGGAT

CGCAGTCTGCAACTCGACTGCGTGAAGTCGGAATCGCTAGTAATCGCGAATCAGAATGTC

GCGGTGAATACGTTCCCGGGCCTTGTACACACCGCCCGTCACACCATGGGAGTGGGTTGC

ACCAGAAGTAGCTAGTCTAACCTTCGGGAGGACGGTACC

>C3_Stenotrophomonas_sp.

GGTGGGGTGGCTTACACATGCAAGTCGAACGGCAGCACAGTAAGAGCTTGCTCTTATGGG

TGGCGAGTGGCGGACGGGTGAGGAATACATCGGAATCTACCTTTTCGTGGGGGATAACGT

AGGGAAACTTACGCTAATACCGCATACGACCTTCGGGTGAAAGCAGGGGACCTTCGGGCC

TTGCGCGGATAGATGAACCGATGTCGGATTAGCTAGTTGGCGGGGTAAAGGCCCACCAAG

GCGACGATCCGTAGCTGGTCTGAGAGGATGATCAGCCACACTGGAACTGAGACACGGTCC

AGACTCCTACGGGAGGCAGCAGTGGGGAATATTGGACAATGGGCGCAAGCCTGATCCAGC

CATACCGCGTGGGTGAAGAAGGCCTTCGGGTTGTAAAGCCCTTTTGTTGGGAAAGAAAAG

CAGTCGATTAATACTCGGTTGTTCTGACGGTACCCAAAGAATAAGCACCGGCTAACTTCG

TGCCAGCAGCCGCGGTAATACGAAGGGTGCAAGCGTTACTCGGAATTACTGGGCGTAAAG

CGTGCGTAGGTGGTTGTTTAAGTCTGTTGTGAAAGCCCTGGGCTCAACCTGGGAATTGCA

GTGGAAACTGGACGACTAGAGTGTGGTAGAGGGTAGTGGAATTCCCGGTGTAGCAGTGAA

ATGCGTAGAGATCGGGAGGAACATCCATGGCGAAGGCAGCTACCTGGACCAACACTGACA

CTGAGGCACGAAAGCGTGGGGAGCAAACAGGATTAGATACCCTGGTAGTCCACGCCCTAA

ACGATGCGAACTGGATGTTGGGTGCAATTTGGCACGCAGTATCGAAGCTAACGCGTTAAG

TTCGCCGCCTGGGGAGTACGGTCGCAAGACTGAAACTCAAAGGAATTGACGGGGGCCCGC

ACAAGCGGTGGAGTATGTGGTTTAATTCGATGCAACGCGAAGAACCTTACCTGGTCTTGA

CATGTCGAGAACTTTCCAGAGATGGATTGGTGCCTTCGGGAACTCGAACACAGGTGCTGC

ATGGCTGTCGTCAGCTCGTGTCGTGAGATGTTGGGTTAAGTCCCGCAACGAGCGCAACCC

TTGTCCTTAGTTGCCAGCACGTAATGGTGGGAACTCTAAGGAGACCGCCGGTGACAAACC

GGAGAAAGGTGGGGATGACGTCAAGTCATCATGGCCCTTACGACCAGGGCTACACACGTA

CTACAATGGTAGGGACAGAGGGCTGCAAACCCGCGAGGGCAAGCCAATCCCAGAAACCCT

ATCTCAGTCCGGATTGGAGTCTGCAACTCGACTCCATGAAGTCGGAATCGCTAGTAATCG

CAGATCAGCATTGCTGCGGTGAATACGTTCCCGGGCCTTGTACACACCGCCCGTCACACC

ATGGGAGTTTGTTGCACCAGAAGCAGGTAGCTTAACCTTCGGGAGGGCGCTGCCACCCGG

TGCCC

>C4_Variovorax_paradoxus

CCGTGGGTATCGCCCTCCTTGCGGTTAAGCTAACTACTTCTGGCAGAACCCGCTCCCATG

GTGTGACGGGCGGTGTGTACAAGACCCGGGAACGTATTCACCGTGACATTCTGATCCACG

ATTACTAGCGATTCCGACTTCACGCAGTCGAGTTGCAGACTGCGATCCGGACTACGACTG

GTTTTATGGGATTAGCTCCCCCTCGCGGGTTGGCAACCCTTTGTACCAGCCATGGTATGA

CGTGTGTAGCCCCACCTATAAGGGCCATGAGGACTTGACGTCATCCCCACCTTCCTCCGG

TTTGTCACCGGCAGTCTCATTAGAGTGCCCAACTGAATGTAGCAACTAATGACAAGGGTT

GCGCTCGTTGCGGGACTTAACCCAACATCTCACGACACGAGCTGACGACAGCCATGCAGC

ACCTGTGTTACGGTTCTCTTTCGAGCACTAAGCCATCTCTGGCGAATTCCGTACATGTCA

AAGGTGGGTAAGGTTTTTCGCGTTGCATCGAATTAAACCACATCATCCACCGCTTGTGCG

GGTCCCCGTCAATTCCTTTGAGTTTCAACCTTGCGGCCGTACTCCCCAGGCGGTCAACTT

CACGCGTTAGCTTCGTTACTGAGTCAGTGAAGACCCAACAACCAGTTGACATCGTTTAGG

GCGTGGACTACCAGGGTATCTAATCCTGTTTGCTCCCCACGCTTTCGTGCATGAGCGTCA

GTACAGGTCCAGGGGATTGCCTTCGCCATCGGTGTTCCTCCGCATATCTACGCATTTCAC

TGCTACACGCGGAATTCCATCCCCCTCTACCGTACTCTAGCTATGCAGTCACAGATGCAG

TTCCCAGGTTGAGCCCGGGGATTTCACAACTGTCTTACATAACCGCCTGCGCACGCTTTA

CGCCCAGTAATTCCGATTAACGCTTGCACCCTACGTATTACCGCGGCTGCTGGCACGTAG

TTAGCCGGTGCTTATTCTTACGGTACCGTCATTAGCCCTCTTTATTAGAAAAGGCCGTTT

CGTTCCGTACAAAAGCAGTTTACAACCCGAAGGCCTTCATCCTGCACGCGGCATGGCTGG

ATCAGGCTTTCGCCCATTGTCCAAAATTCCCCACTGCTGCCTCCCGTAGGAGTCTGGGCC

GTGTCTCAGTCCCAGTGTGGCTGGTCGTCCTCTCAGACCAGCTAAAGATCGAAGGCTTGG

TGAGCCTTTACCTCACCAACTACCTAATCTGCCATCGGCCGCTCCATTCGCGCAAGGTCT

TGCGATCCCCTGCTTTCATCCGTAGATCGTATGCGGTATTAGCACAGCTTTCGCTGCGTT

ATCCCCCACGATTGGGCACGTTCCGATGTATTACTCACCCGTTCGCCACTCGCCGCCAGG

ATTGCTCCCGCGCTGCCGTTCGACTTGCATGTTAAGG

>C5_Variovorax_sp.

CCGTGGTATCGCCCTCCTTGCGGTTAAGCTAACTACTTCTGGCAGAACCCGCTCCCATGG

TGTGACGGGCGGTGTGTACAAGACCCGGGAACGTATTCACCGTGACATTCTGATCCACGA

TTACTAGCGATTCCGACTTCACGCAGTCGAGTTGCAGACTGCGATCCGGACTACGACTGG

TTTTATGGGATTAGCTCCCCCTCGCGGGTTGGCAACCCTTTGTACCAGCCATTGTATGAC

GTGTGTAGCCCCACCTATAAGGGCCATGAGGACTTGACGTCATCCCCACCTTCCTCCGGT

TTGTCACCGGCAGTCTCATTAGAGTGCCCAACTGAATGTAGCAACTAATGACAAGGGTTG

CGCTCGTTGCGGGACTTAACCCAAACATCTCACGACACGAGCTGACGACAGCCATGCAGC

ACCTGTGTTACGGTTCTCTTTCGAGCACTAAGCCATCTCTGGCGAATTCCGTACATGTCA

AAGGTGGGTAAGGTTTTTCGCGTTGCATCGAATTAAACCACATCATCCACCGCTTGTGCG

GGTCCCCGTCAATTCCTTTGAGTTTCAACCTTGCGGCCGTACTCCCCAGGCGGTCAACTT

CACGCGTTAGCTTCGTTACTGAGTCAGTGAAGACCCAACAACCAGTTGACATCGTTTAGG

GCGTGGACTACCAGGGTATCTAATCCTGTTTGCTCCCCACGCTTTCGTGCATGAGCGTCA

GTACAGGTCCAGGGGATTGCCTTCGCCATCGGTGTTCCTCCGCATATCTACGCATTTCAC

TGCTACACGCGGAATTCCATCCCCCTCTACCGTACTCTAGCTATGCAGTCACAGATGCAG

TTCCCAGGTTGAGCCCGGGGATTTCACAACTGTCTTACATAACCGCCTGCGCACGCTTTA

CGCCCAGTAATTCCGATTAACGCTTGCACCCTACGTATTACCGCGGCTGCTGGCACGTAG

TTAGCCGGTGCTTATTCTTACGGTACCGTCATTAGCCCTCTTTATTAGAAAAGGCCGTTT

CGTTCCGTACAAAAGCAGTTTACAACCCGAAGGCCTTCATCCTGCACGCGGCATGGCTGG

ATCAGGCTTTCGCCCATTGTCCAAAATTCCCCACTGCTGCCTCCCGTAGGAGTCTGGGCC

GTGTCTCAGTCCCAGTGTGGCTGGTCGTCCTCTCAGACCAGCTACAGATCGAAGGCTTGG

TGAGCCTTTACCTCACCAACTACCTAATCTGCCATCGGCCGCTCCATTCGCGCAAGGTCT

TGCGATCCCCTGCTTTCATCCGTAGATCGTATGCGGTATTAGCACAGCTTTCGCTGCGTT

ATCCCCCACGATTGGGCACGTTCCGATGTATTACTCACCCGTTCGCCACTCGCCGCCAGG

ATTGCTCCCGCGCTGCCGTTCGACTTGCAT

>C6_Microbacterium_sp.

GGGGGGCTTAACCATGCAGTCGAACGGTGAAGCCCAGCTTGCTGGGTGGATCAGTGGCGA

ACGGGTGAGTAACACGTGAGCAACCTGCCCCTGACTCTGGGATAAGCGCTGGAAACGGCG

TCTAATACTGGATACGAGCCACGACCGCATGGTCAGTGGTTGGAAAGATTTTTCGGTTGG

GGATGGGCTCGCGGCCTATCAGCTTGTTGGGGAGGTAATGGCTCACCAAGGCGTCGACGG

GTAGCCGGCCTGAGAGGGTGACCGGCCACACTGGGACTGAGACACGGCCCAGACTCCTAC

GGGAGGCAGCAGTGGGGAATATTGCACAATGGGCGGAAGCCTGATGCAGCAACGCCGCGT

GAGGGATGACGGCCTTCGGGTTGTAAACCTCTTTTAGCAGGGAAGAAGCGAAAGTGACGG

TACCTGCAGAAAAAGCGCCGGCTAACTACGTGCCAGCAGCCGCGGTAATACGTAGGGCGC

AAGCGTTATCCGGAATTATTGGGCGTAAAGAGCTCGTAGGCGGTTTGTCGCGTCTGCTGT

GAAATCTGGGGGCTCAACCCCCAGCCTGCAGTGGGTACGGGCAGACTAGAGTGCGGTAGG

GGAGATTGGAATTCCTGGTGTAGCGGTGGAATGCGCAGATATCAGGAGGAACACCGATGG

CGAAGGCAGATCTCTGGGCCGTAACTGACGCTGAGGAGCGAAAGGGTGGGGAGCAAACAG

GCTTAGATACCCTGGTAGTCCACCCCGTAAACGTTGGGAACTAGTTGTGGGGTCCATTCC

ACGGATTCCGTGACGCAGCTAACGCATTAAGTTCCCCGCCTGGGGAGTACGGCCGCAAGG

CTAAAACTCAAAGGAATTGACGGGGACCCGCACAAGCGGCGGAGCATGCGGATTAATTCG

ATGCAACGCGAAGAACCTTACCAAGGCTTGACATATACGAGAACGGGCCAGAAATGGTCA

ACTCTTTGGACACTCGTAAACAGGTGGTGCATGGTTGTCGTCAGCTCGTGTCGTGAGATG

TTGGGTTAAGTCCCGCAACGAGCGCAACCCTCGTTCTATGTTGCCAGCACGTAATGGTGG

GAACTCATGGGATACTGCCGGGGTCAACTCGGAGAAAGGTGGGGATGACGTCAAATCATC

ATGCCCCTTATGTCTTGGGCTTCACGCATGCTACAATGGCCGGTACAAAGGGCTGCAATA

CCGCGAGGTGGAACGAATCCCAAAAAGCCGGTCCCAGTTCGGATTGAGGTCTGCAACTCG

ACCTCATGAAGTCGGAGTCGCTAGTAATCGCAGATCAGCAACGCTGCGGTGAATACGTTC

CCGGGTCTTGTACACACCGCCCGTCAAGTCATGAAAGTCGGTAACACCTGAAGCCGGTGG

CCTAACCCTTGTGGAGGGAGCCGTCGAAGG

>C7_Pseudomonas_putida

GGTACCGTCCTCCCGAGGGTTAGACTAGCTACTTCTGGTGCAACCCACTCCCATGGTGTG

ACGGGCGGTGTGTACAAGGCCCGGGAACGTATTCACCGCGACATTCTGATTCGCGATTAC

TAGCGATTCCGACTTCACGCAGTCGAGTTGCAGACTGCGATCCGGACTACGATCGGTTTT

GTGAGATTAGCTCCACCTCGCGGCTTGGCAACCCTCTGTACCGACCATTGTAGCACGTGT

GTAGCCCAGGCCGTAAGGGCCATGATGACTTGACGTCATCCCCACCTTCCTCCGGTTTGT

CACCGGCAGTCTCCTTAGAGTGCCCACCATAACGTGCTGGTAACTAAGGACAAGGGTTGC

GCTCGTTACGGGACTTAACCCAACATCTCACGACACGAGCTGACGACAGCCATGCAGCAC

CTGTGTCAGAGTTCCCGAAGGCACCAATCCATCTCTGGAAAGTTCTCTGCATGTCAAGGC

CTGGTAAGGTTCTTCGCGTTGCTTCGAATTAAACCACATGCTCCACCGCTTGTGCGGGCC

CCCGTCAATTCATTTGAGTTTTAACCTTGCGGCCGTACTCCCCAGGCGGTCAACTTAATG

CGTTAGCTGCGCCACTAAAATCTCAAGGATTCCAACGGCTAGTTGACATCGTTTACGGCG

TGGACTACCAGGGTATCTAATCCTGTTTGCTCCCCACGCTTTCGCACCTCAGTGTCAGTA

TCAGTCCAGGTGGTCGCCTTCGCCACTGGTGTTCCTTCCTATATCTACGCATTTCACCGC

TACACAGGAAATTCCACCACCCTCTACCGTACTCTAGCTTGCCAGTTTTGGATGCAGTTC

CCAGGTTGAGCCCGGGGCTTTCACATTCAACTTAACAAACCACCTACGCGCGCTTTACGC

CCAGTAATTCCGATTAACGCTTGCACCCTCTGTATTACCGCGGCTGCTGGCACAGAGTTA

GCCGGTGCTTATTCTGTCGGTAACGTCAAAACAGCAAGGTATTCGCTTACTGCCCTTCCT

CCCAACTTAAAGTGCTTTACAATCCGAAGACCTTCTTCACACACGCGGCATGGCTGGATC

AGGCTTTCGCCCATTGTCCAATATTCCCCACTGCTGCCTCCCGTAGGAATCTGGACCGTG

TCTCAGTTCCAGTGTGACTGATCATCCTCTCAGACCAGTTACGGATCGTCGCCTTGGTGA

GCCATTACCCCACCAACTAGCTAATCCGACCTAGGCTCATCTGATAGCGCAAGGCCCGAA

GGTCCCCTGCTTTCTCCCGTAGGACGTATGCGGTATTAGCGTTCCTTTCGAAACGTTGTC

CCCCACTATCAGGCAGATTCCTAGGCATTACTCACCCGTCCGCCGCTGAATCGAAGAGCA

AGCTCTTCTCATCCGCTCGACTTGCATGTGT

>C8_Stenotrophomonas_sp.

CAGCGCCCTCCCGAAGGTTAAGCTACCTGCTTCTGGTGCAACAAACTCCCATGGTGTGAC

GGGCGGTGTGTACAAGGCCCGGGAACGTATTCACCGCAGCAATGCTGATCTGCGATTACT

AGCGATTCCGACTTCATGGAGTCGAGTTGCAGACTCCAATCCGGACTGAGATAGGGTTTC

TGGGATTGGCTTACCGTCGCCGGCTTGCAGCCCTCTGTCCCTACCATTGTAGTACGTGTG

TAGCCCTGGCCGTAAGGGCCATGATGACTTGACGTCATCCCCACCTTCCTCCGGTTTGTC

ACCGGCGGTCTCCTTAGAGTTCCCACCATTACGTGCTGGCAACTAAGGACAAGGGTTGCG

CTCGTTGCGGGACTTAACCCAACATCTCACGACACGAGCTGACGACAGCCATGCAGCACC

TGTGTTCGAGTTCCCGAAGGCACCAATCCATCTCTGGAAAGTTCTCGACATGTCAAGGCC

AGGTAAGGTTCTTCGCGTTGCATCGAATTAAACCACATACTCCACCGCTTGTGCGGGCCC

CCGTCAATTCCTTTGAGTTTCAGTCTTGCGACCGTACTCCCCCAGGCGGCGAACTTAACG

CGTTAGCTTCGATACTGCGTGCCAAATTGCACCCAACATCCAGTTCGCATCGTTTAGGGC

GTGGACTACCAGGGTATCTAATCCTGTTTGCTCCCCACGCTTTCGTGCCTCAGTGTCAGT

GTTGGTCCAGGTAGCTGCCTTCGCCATGGATGTTCCTCCCGATCTCTACGCATTTCACTG

CTACACCGGGAATTCCGCTACCCTCTACCACACTCTAGTTGTCCAGTTTCCACTGCAGTT

CCCAGGTTGAGCCCAGGGCTTTCACAACAGACTTAAACAACCACCTACGCACGCTTTACG

CCCAGTAATTCCGAGTAACGCTTGCACCCTTCGTATTACCGCGGCTGCTGGCACGAAGTT

AGCCGGTGCTTATTCTTTGGGTACCGTCATCCCAACCAGGTATTAACCGGCTGGATTTCT

TTCCCAACAAAAGGGCTTTACAACCCGAAGGCCTTCTTCACCCACGCGGTATGGCTGGAT

CAGGCTTGCGCCCATTGTCCAATATTCCCCACTGCTGCCTCCCGTAGGAGTCTGGACCGT

GTCTCAGTTCCAGTGTGGCTGATCATCCTCTCAGACCAGCTACGGATCGTCGCCTTGGTG

GGCCTTTACCCCGCCAACTAGCTAATCCGACATCGGCTCATTCAATCGCGCAAGGCCCGA

AGGTCCCCTGCTTTCACCCGTAGGTCGTATGCGGTATTAGCGTAAGTTTCCCTACGTTAT

CCCCCACGAAAAAGTAGATTCCGATGTATTCCTCACCCGTCCGCCACTCGCCACCCATAA

GAGCAAGCTCTTACTGTGCTGCCGTTCGACTTGCAT

>C9_Stenotrophomonas_rhizophila

ATGCAAGTCGAACGGCAGCACAGTAAGAGCTTGCTCTTATGGGTGGCGAGTGGCGGACGG

GTGAGGAATACATCGGAATCTACCTTTTCGTGGGGGATAACGTAGGGAAACTTACGCTAA

TACCGCATACGACCTTCGGGTGAAAGCAGGGGACCTTCGGGCCTTGCGCGGATAGATGAG

CCGATGTCGGATTAGCTAGTTGGCGGGGTAAAGGCCCACCAAGGCGACGATCCGTAGCTG

GTCTGAGAGGATGATCAGCCACACTGGAACTGAGACACGGTCCAGACTCCTACGGGAGGC

AGCAGTGGGGAATATTGGACAATGGGCGCAAGCCTGATCCAGCCATACCGCGTGGGTGAA

GAAGGCCTTCGGGTTGTAAAGCCCTTTTGTTGGGAAAGAAAAGCAGTCGATTAATACTCG

GTTGTTCTGACGGTACCCAAAGAATAAGCACCGGCTAAATTCGTGCCAGCAGCCGCGGTA

ATACGAAGGGTGCAAGCGTTACTCGGAATTACTGGGCGTAAAGCGTGCGTAGGTGGTTGT

TTAAGTCTGTTGTGAAAGCCCTGGGCTCAACCTGGGAATTGCAGTGGATACTGGACGACT

AGAGTGTGGTAGAGGGTAGTGGAATTCCCGGTGTAGCAGTGAAATGCGTAGAGATCGGGA

GGAACATCCATGGCGAAGGCAGCTACCTGGACCAACACTGACACTGAGGCACGAAAGCGT

GGGGAGCAAACAGGATTAGATACCCTGGTAGTCCACGCCCTAAACGATGCGAACTGGATG

TTGGGTGCAATTTGGCACGCAGTATCGAAGCTAACGCGTTAAGTTCGCCGCCTGGGGAGT

ACGGTCGCAAGACTGAAACTCAAAGGAATTGACGGGGGCCCGCACAAGCGGTGGAGTATG

TGGTTTAATTCGATGCAACGCGAAGAACCTTACCTGGTCTTGACATGTCGAGAACTTTCC

AGAGATGGATTGGTGCCTTCGGGAACTCGAACACAGGTGCTGCATGGCTGTCGTCACCTC

GTGTCGTGAGATGTTGGGTTAAGTCCCGCAACGAGCGCAACCCTTGTCCTTAGTTGCCAG

CACGTAATGGTGGGAACTCTAAGGAGACCGCCGGTGACAAACCGGAGGAAGGTGGGGATG

ACGTCAAGTCATCATGGCCCTTACGACCAGGGCTACACACGTACTACAATGGTAGGGACA

GAGGGCTGCAAACCCGCGAGGGCAAGCCAATCCCAGAAACCCTATCTCAGTCCGGATTGG

AGTCTGCAACTCGACTCCATGAAGTCGGAATCGCTAGTAATCGCAGATCAGCATTGCTGC

GGTGAATACGTTCCCGGGCCTTGTACACACCGCCCGTCACACCATGGGAGTTTGTTGCAC

CAGAAGCAGGTAGCTTAAACCTTCGGGAGGGCGCTTGCCACGGTGCC

>C10_Pseudomonas_sp.

CATGCAAGTCGAGCGGTAGAGAGGAGCTTGCTTCTCTTGAGAGCGGCGGACGGGTGAGTA

ATGCCTAGGAATCTGCCTGGTAGTGGGGGATAACGTTCGGAAACGGACGCTAATACCGCA

TACGTCCTACGGGAGAAAGCAGGGGACCTTCGGGCCTTGCGCTATCAGATGAGCCTAGGT

CGGATTAGCTAGTTGGTGAGGTAATGGCTCACCAAGGCGACGATCCGTAACTGGTCTGAG

AGGATGATCAGTCACACTGGAACTGAGACACGGTCCAGACTCCTACGGGAGGCAGCAGTG

GGGAATATTGGACAATGGGCGAAAGCCTGATCCAGCCATGCCGCGTGTGTGAAGAAGGTC

TTCGGATTGTAAAGCACTTTAAGTGGGGAGGAAGGGCAGTAAATTTAATACTTTGCTGTT

TTGACGTTACCGACAGAATTAAGCACCGGCTAAACTCTGTGCCAGCAGCCGCGGTAATAC

AGAGGGTGCAAGCGTTAATCGGAATTACTGGGCGTAAAGCGCGCGTAGGTGGTTTGTTAA

GGTGGGATGTGAAATCCCCGGGGCTCAACCTGGGAACTGCATTCAAAACTGACTGACTAG

AGTATGGTAGAGGGTGGGTGGAAATTTCCTGTGGAGCGGTGAAATGCGTAGATATAGGAA

GGAAACACCAGTGGCGAAAGGCGACCACCTGGACTAATACTGACACTGAGGTGCGAAAGC

GTGGGGAGCAAACAGGATTAGATACCCTGGTAGTCCACGCCGTAAACGATGTCAACTAGC

CGTTGGAAGCCTTGAGCTTTTAGTGGCGCAGCTAACGCATTTAAGTTGACCGCCTGGGGA

GTACGGCCGCAAGGTTAAAACTCAAATGAATTGACGGGGGCCCGCACAAGCGGTGGAGCA

TGTGGTTTAATTCGAAGCAACGCGAAGAACCTTACCAGGCCTTGACATCCAATGAACTTT

CTAGAGATAGATTGGTGCCTTCGGGAACATTGAGACAGGTGCTGCATGGCTGTCGTCAGC

TCGTGTCGTGAGATGTTGGGTTAAGTCCCGTAACGAGCGCAACCCTTGTCCTTAGTTACC

AGCACGTCATGGTGGGCACTCTAAGGAGACTGCCGGTGACAAACCGGAGGAAGGTGGGGA

TGACGTCAAGTCATCATGGCCCTTACGGCCTGGGCTACACACGTGCTACAATGGTCGGTA

CAGAGGGTTGCCAAGCCGCGAGGTGGAGCTAATCCCACAAAACCGATCGTAGTCCGGATC

GCAGTCTGCAACTCGACTGCGTGAAGTCGGAATCGCTAGTAATCGCGAATCAGAATGTCG

CGGTGAATACGTTCCCGGGCCTGTACACACCGCCCGTCACACCATGGGAGTGGGTGCACC

AGAAGTAGCTAGTCTAACTTCGGGAGGGACGG

>C11_Plantibacter_sp.

GAAGCCCAGCTTGCTGGGTGGATTAGTGGCGAACGGGTGAGTAACACGTGAGTAACCTGC

CCTTGACTCTGGGATAAGCGTTGGAAACGACGTCTAATACCGGATACGAGCTTCCACCGC

ATGGTGAGTTGCTGGAAAGAATTTTGGTCAAGGATGGACTCGCGGCCTATCAGCTTGTTG

GTGAGGTAACGGCTCACCAAGGCGACGACGGGTAGCCGGCCTGAGAGGGTGACCGGCCAC

ACTGGGACTGAGACACGGCCCAGACTCCTACGGGAGGCAGCAGTGGGGAATATTGCACAA

TGGGCGAAAGCCTGATGCAGAAACGCCGCCTGAGGGACGACGGCCTTCGGGTTGTAAACC

TCTTTTAGCAGGGAAGAAGCGAAAGTGACGGTACCTGCAGAAAAAGCACCGGCAAACTAC

GTGCCAGCAGCCGCGGTAATACGTAGGGTGCAAGCGTTGTCCGGAATTATTGGGCGTAAA

GAGCTCGTAGGCGGTTTGTCGCGTCTGCTGTGAAATCCCGAGGCTCAACCTCGGGTCTGC

AGTGGGTACGGGCAGACTAGAATGCGGTAGGGGAGATTGGAATTCCTGGTGTAGCGGTGG

AATGCGCAGATATCAGGAGGAACACCGATGGCGAAGGCAGATCTCTGGGCCGCTACTGAC

GCTGAGGAGCGAAAGGGTGGGGAGCAAACAGGCTTAGATACCCTGGTAGTCCACCCCGTA

AACGTTGGGCGCTAGATGTGGGGACCATTCCACGGTTTCCGTGTCGTAGCTAACGCATTA

AGCGCCCCGCCGGGGGAGTACGGCCGCAAGGCTAAAACTCAAAGGAATTGACGGGGGCCC

GCACAAGCGGCGGAGCAGGCGGATTAATTCGATGCAACGCGAAGAACCTTACCAAGGCTT

GACATATACAAGAACGGGCCAGAAATGGTCAACTCTTTGGACACTCGTAAACAGGTGGTG

CATGGTTGTCGTCAGCTCGTGTCGTGAGATGTTGGGTTAAGTCCCGCAACGAGCGCAACC

CTCGTTCTATGTTGCCAGCACGTAATGGTGGGAACTCATGGGATACTGCCGGGGTCAACT

CGGAGGAAGGTGGGGACGACGTCAAATCATCATGCCCCTTATGTCTTGGGCTTCACGCAT

GCTACAATGGCCAGTACAAAGGGCTGCAATACCGTAAGGTGGAGCGAATCCCAAAAAGCT

GGTCCCAGTTCGGATTGAGGTCTGCAACTCGACCTCATGAAGTCGGAGTCGCTAGTAATC

GCAGATCAGCAACGCTGCGGTGAATACGTTCCCGGGCCTTGTACACACCGCCCGTCAAGT

CATGAAAGTCGGTAACACCCGAAGCCAGTGGCCTAACCGCAAGGAGGAGCGTCTAA

>C12_Stenotrophomonas_rhizophila

GCAAGTCGAACGGCAGCACAGTAAGAGCTTGCTCTTATGGGTGGCGAGTGGCGGACGGGT

GAGGAATACATCGGAATCTACCTTTTCGTGGGGGATAACGTAGGGAAACTTACGCTAATA

CCGCATACGACCTTCGGGTGAAAGCAGGGGACCTTCGGGCCTTGCGCGGATAGATGAGCC

GATGTCGGATTAGCTAGTTGGCGGGGTAAAGGCCCACCAAGGCGACGATCCGTAGCTGGT

CTGAGAGGATGATCAGCCACACTGGAACTGAGACACGGTCCAGACTCCTACGGGAGGCAG

CAGTGGGGAATATTGGACAATGGGCGCAAGCCTGATCCAGCCATACCGCGTGGGTGAAGA

AGGCCTTCGGGTTGTAAAGCCCTTTTGTTGGGAAAGAAAAGCAGTCGATTAATACTCGGT

TGTTCTGACGGTACCCAAAGAATAAGCACCGGCTAACTTCGTGCCAGCAGCCGCGGTAAT

ACGAAGGGTGCAAGCGTTACTCGGAATTACTGGGCGTAAAGCGTGCGTAGGTGGTTGTTT

AAGTCTGTTGTGAAAGCCCTGGGCTCAACCTGGGAATTGCAGTGGATACTGGGCGACTAG

AGTGTGGTAGAGGGTAGTGGAATTCCCGGTGTAGCAGTGAAATGCGTAGAGATCGGGAGG

AACATCCATGGCGAAGGCAGCTACCTGGACCAACACTGACACTGAGGCACGAAAGCGTGG

GGAGCAAACAGGATTAGATACCCTGGTAGTCCACGCCCTAAACGATGCGAACTGGATGTT

GGGTGCAATTTGGCACGCAGTATCGAAGCTAACGCGTTAAGTTCGCCGCCTGGGGAGTAC

GGTCGCAAGACTGAAACTCAAAGGAATTGACGGGGGCCCGCACAAGCGGTGGAGTATGTG

GTTTAATTCGATGCAACGCGAAGAACCTTACCTGGTCTTGACATGTCGAGAACTTTCCAG

AGATGGATTGGTGCCTTCGGGAACTCGAACACAGGTGCTGCATGGCTGTCGTCAGCTCGT

GTCGTGAGATGTTGGGTTAAGTCCCGCAACGAGCGCAACCCTTGTCCTTAGTTGCCAGCA

CGTAATGGTGGGAACTCTAAGGAGACCGCCGGTGACAAACCGGAGGAAGGTGGGGATGAC

GTCAAGTCATCATGGCCCTTACGACCAGGGCTACACACGTACTACAATGGTAGGGACAGA

GGGCTGCAAACCCGCGAGGGCAAGCCAATCCCAGAAACCCTATCTCAGTCCGGATTGGAG

TCTGCAACTCGACTCCATGAAGTCGGAATCGCTAGTAATCGCAGATCAGCATTGCTGCGG

TGAATACGTTCCCGGGCCTTGTACACACCGCCCGTCACACCATGGGAGTTTGTTGCACCA

GAAGCAGGTAGCTTAACCTTCGGGAGGGCGCTTGCCC

>D1_Pseudomonas_sp.

GGTAACCGTCCTCCCGAAGGTTAGACTAGCTACTTCTGGTGCAACCCACTCCCATGGTGT

GACGGGCGGTGTGTACAGGCCCGGGAACGTATTCACCGCGACATTCTGATTCGCGATTAC

TAGCGATTCCGACTTCACGCAGTCGAGTTGCAGACTGCGATCCGGACTACGATCGGTTTT

GTGGGATTAGCTCCACCTCGCGGCTTGGCAACCCTCTGTACCGACCATTGTAGCACGTGT

GTAGCCCAGGCCGTAAGGGCCATGATGACTTGACGTCATCCCCACCTTCCTCCGGTTTGT

CACCGGCAGTCTCCTTAGAGTGCCCACCATGACGTGCTGGTAACTAAGGACAAGGGTTGC

GCTCGTTACGGGACTTAACCCAACATCTCACGACACGAGCTGACGACAGCCATGCAGCAC

CTGTCTCATTGTTCCCGAAGGCACCAATCTATCTCTAGAAGGTTCATTGGATGTCAAGGC

CTGGTAAGGTTCTTCGCGTTGCTTCGAATTAAACCACATGCTCCACCGCTGGTGCGGGCC

CCCGTCAATTCATTTGAGTTTTAACCTTGCGGCCGTACTCCCCAGGCGGTCAACTTAATG

CGTTAGCTGCGCCACTAAGAGCTCAAGGCTTCCAACGGCTAGTTGACATCGTTTACGGCG

TGGACTACCAGGGTATCTAATCCTGTTTGCTCCCCACGCTTTCGCACCTCATTGTCAGTA

TTAGTCCAGGTGGTCGCCTTCGCCACTGGTGTTCCTTCCTATATCTACGCATTTCACCGC

TCCACAGGAAATTCCACCACCCTCTACCATACTCTAGTCAGTCAGTTTTGAATGCAGTTC

CCAGGTTGAGCCCGGGGATTTCACATCCAACTTAACAAACCACCTACGCGCGCTTTACGC

CCAGTAATTCCAATAAACGCTGGCACCCTCTGTATTACCGCGGCTGCGGGCACAGAGTTA

GCCGGTGCTTATTCTGTCGGTAACGTCAAAACAGCAAAGTATTAATTTACTGCCCTTCCT

CCCAACTTAAAGTGCTTTACAATCCGAAGACCTTCTTCACACACGCGGCATGGCTGGATC

AGGCTTTCGCCCATTGTCCAATATTCCCCACTGCTGCCTCCCGTAGGAGTCTGGACCGTG

TCTCAGTTCCAGTGTGACTGATCATCCTCTCAGACCAGTTACGGATCGTCGCCTTGGTGA

GCCATTACCTCACCAACTAGCTAATCCGACCTAGGCTCATCTGATAGCGCAAGGCCCGAA

GGTCCCCTGCTTTCTCCCGTAGGACGTATGCGGTATTAGCGTCCGTTTCCGAACGTTATC

CCCCACTACCAGGCAGATTCCTAGGCATTACTCACCCGTCCGCCGCTCTCAAGAGAAGCA

AGCTCCTCTCTACCGCTCGACCTTGCA

>D2_Stenotrophomonas_rhizophila

AGCTACCTGCTTCTGGTGCAACAAACTCCCATGGTGTGACGGGCGGTGTGTACAAGGCCC

GGGAACGTATTCACCGCAGCAATGCTGATCTGCGATTACTAGCGATTCCGACTTCATGGA

GTCGAGTTGCAGACTCCAATCCGGACTGAGATAGGGTTTCTGGGATTGGCTTGCCCTCGC

GGGTTTGCAGCCCTCTGTCCCTACCATTGTAGTACGTGTGTAGCCCTGGTCGTAAGGGCC

ATGATGACTTGACGTCATCCCCACCTTCCTCCGGTTTGTCACCGGCGGTCTCCTTAGAGT

TCCCACCATTACGTGCTGGCAACTAAGGACAAGGGTTGCGCTCGTTGCGGGACTTAACCC

AACATTCTCACGACACGAGCTGACGACAGCCATGCAGCACCTGTGTTCGAGTTCCCGAAG

GCACCAATCCATCTTCTGGAAAGTTCTCGACATGTCAAGACCAGGTAAGGTTCTTCGCGT

TGCATCGAATTAAACCACCATACTCCACCGCTTGTGCGGGCCCCCGTCAATTCCTTTGAG

TTTCAGTCTTGCGACCGTACTCCCCAGGCGGCGAACTTAACGCGTTAGCTTCGATACTGC

GTGCCAAATTGCACCCAACATCCAGTTCGCATCGTTTAGGGCGTGGACTACCAGGGTATC

TAATCCTGTTTGCTCCCCACGCTTTCGTGCCTCAGTGTCAGTGTTGGTCCAGGTAGCTGC

CTTCGCCATGGATGTTCCTCCCGATCTCTACGCATTTCACTGCTACACCGGGAATTCCAC

TACCCTCTACCACACTCTAGTCGCCCAGTATCCACTGCAATTCCCAGGTTGAGCCCAGGG

CTTTCACAACAGACTTAAACAACCACCTACGCACGCTTTACGCCCAGTAATTCCGAGTAA

CGCTTGCACCCTTCGTATTACCGCGGCTGCTGGCACGAAGTTAGCCGGTGCTTATTCTTT

GGGTACCGTCAGAACAACCGAGTATTAATCGACTGCTTTTCTTTCCCAACAAAAGGGCTT

TACAACCCGAAGGCCTTCTTCACCCACGCGGTATGGCTGGATCAGGCTTGCGCCCATTGT

CCAATATTCCCCACTGCTGCCTCCCGTAGGAGTCTGGACCGTGTCTCAGTTCCAGTGTGG

CTGATCATCCTCTCAGACCAGCTACGGATCGTCGCCTTGGTGGGCCTTTACCCCGCCAAC

TAGCTAATCCGACATCGGCTCATCTATCCGCGCAAGGCCCGAAGGTCCCCTGCTTTCACC

CGAAGGTCGTATGCGGTATTAGCGTAAGTTTCCCTACGTTATCCCCCACGAAAAGGTAGA

TTCCGATGTATTCCTCACCCGTCCGCCACTCGCCACCCATAAGAGCAAGCTCTTACTGTG

CTGCCGTTCGACTTGCATGT

>D3_Stenotrophomonas_sp.

ATGCAAGTCGAACGGCAGCACAGTAAGAGCTTGCTCTTATGGGTGGCGAGTGGCGGACGG

GTGAGGAATACATCGGAATCTACCTTTTCGTGGGGGATAACGTAGGGAAACTTACGCTAA

TACCGCATACGACCTTCGGGTGAAAGCAGGGGACCTTCGGGCCTTGCGCGGATAGATGAG

CCGATGTCGGATTAGCTAGTTGGCGGGGTAAAGGCCCACCAAGGCGACGATCCGTAGCTG

GTCTGAGAGGATGATCAGCCACACTGGAACTGAGACACGGTCCAGACTCCTACGGGAGGC

AGCAGTGGGGAATATTGGACAATGGGCGCAAGCCTGATCCAGCCATACCGCGTGGGTGAA

GAAGGCCTTCGGGTTGTAAAGCCCTTTTGTTGGGAAAGAAAAGCAGTCGATTAATACTCG

GTTGTTCTGACGGTACCCAAAGAATAAGCACCGGCTAACTTCGTGCCAGCAGCCGCGGTA

ATACGAAGGGTGCAAGCGTTACTCGGAATTACTGGGCGTAAAGCGTGCGTAGGTGGTTGT

TTAAGTCTGTTGTGAAAGCCCTGGGCTCAACCTGGGAATTGCAGTGGAAACTGGACGACT

AGAGTGTGGTAGAGGGTAGTGGAATTCCCGGTGTAGCAGTGAAATGCGTAGAGATCGGGA

GGAACATCCATGGCGAAGGCAGCTACCTGGACCAACACTGACACTGAGGCACGAAAGCGT

GGGGAGCAAACAGGATTAGATACCCTGGTAGTCCACGCCCTAAACGATGCGAACTGGATG

TTGGGTGCAATTTGGCACGCAGTATCGAAGCTAACGCGTTAAGTTCGCCGCCTGGGGGAG

TACGGTCGCAAGACTGAAACTCAAAGGAATTGACGGGGGCCCGCACAAGCGGTGGAATTA

TGTGGGTTTAATTCGATGCAACGCGAAGAACCTTACCTGGTCTTGACATGTCGAGAACTT

TCAAAAGATGGATTGGTGCCTTCGGGAACTCGAACACAGGTGCTGCATGGCTGTCGTCAG

CTCGTGTCCTGAAAAGTGTGGGTTAAGTCCCGCAACGAGCGCAACCCTTGTCCTTAGTTG

CCAGCACGTAATGGTGGGAACTCTAAGGAGACCGCCGGTGACAAACCGGAGGAAGGTGGG

GATGACGTCAAGTCATCATGGCCCTTACGACCAGGGCTACACACGTACTACAATGGTAGG

GACAGAGGGCTGCAAACCCGCGAGGGCAAGCCAATCCCAGAAACCCTATCTCAGTCCGGA

TTGGAGTCTGCAACTCGACTCCATGAAGTCGGAATCGCTAGTAATCGCAGATCAGCATTG

CTGCGGTGAATACGTTCCCGGGCCTTGTACACACCGCCCGTCACACCATGGGAGTTTGTT

GCACCAGAAGCAGGTAGCTTAACCTTCGGGAGGGCGCTGCC

>D4_Microbacterium_sp._reverse

TTCGACGGCTCCCTCCACAAGGGTTAGGCCACCGGCTTCAGGTGTTACCGACTTTCATGA

CTTGACGGGCGGTGTGTACAAGACCCGGGAACGTATTCACCGCAGCGTTGCTGATCTGCG

ATTACTAGCGACTCCAACTTCATGAGGTCGAGTTGCAGACCTCAATCCGAACTGGGACCG

GCTTTTTGGGATTCGCTCCACCTCGCGGTATTGCAGCCCTTTGTACCGGCCATTGTAGCA

TGCGTGAAGCCCAAGACATAAGGGGCATGATGATTTGACGTCATCCCCACCTTCCTCCGA

GTTGACCCCGGCAGTATCCCATGAGTTCCCACCATTACGTGCTGGCAACATAGAACGAGG

GTTGCGCTCGTTGCGGGACTTAACCCAACATCTCACGACACGAGCTGACGACAACCATGC

ACCACCTGTTTACGAGTGTCCAAAGAGTTGACCATTTCTGGCCCGTTCTCGTATATGTCA

AGCCTTGGTAAGGTTCTTCGCGTTGCATCGAATTAATCCGCATGCTCCGCCGCTTGTGCG

GGTCCCCGTCAATTCCTTTGAGTTTTAGCCTTGCGGCCGTACTCCCCAGGCGGGGAACTT

AATGCGTTAGCTGCGTCACGGAATCCGTGGAATGGACCCCACAACTAGTTCCCAACGTTT

ACGGGGTGGACTACCAGGGTATCTAAGCCTGTTTGCTCCCCACCCTTTCGCTCCTCAGCG

TCAGTTACGGCCCAGAGATCTGCCTTCGCCATCGGTGTTCCTCCTGATATCTGCGCATTC

CACCGCTACACCAGGAATTCCAATCTCTCCTACCGCACTCTAGTCTGCCCGTACCCACTG

CAGGCCGGAGGTTGAGCCTCCGGATTTCACAGCAGACGCGACAAACCGCCTACGAGCTCT

TTACGCCCAATAATTCCGGATAACGCTTGCGCCCTACGTATTACCGCGGCTGCTGGCACG

TAGTTAGCCGGCGCTTTTTCTGCAGGTACCGTCACTTTCGCTTCTTCCCTGCTAAAAGAG

GTTTACAACCCGAAGGCCGTCATCCCTCACGCGGCGTTGCTGCATCAGGCTTGCGCCATT

GTGCAGTATTCCCACTGCTGCATCCGTAGGAGTCTGGGCCGTGTCTCAATCCAGTGTGGC

GGTCACCCTCTC

>D5_Streptomyces_sp._forward

NTTANNMNTGCAAGTCGAACGATGAAGCCCTTCKGGGTGGATTASTGGCGAACGGRTGAGTNACACRTGGKCAAKCTGCC

STTCAYTCTGGGASRAGCCCTGKAAACGGGGTCTAATACCRGATAACACTCTGTCCCGCATGGGRCGGGGTWAMAAGCTC

CSGCGGTGAAGGATGAGCCCGCGGCCTATCAKCTTGTTGGTGGGGTAATGGCSTACCAAGGCGACRACRGGTAGMCGGCC

TGAGAGGGCRACMGGCCACACTGGGACTGAGACACGGCCCASACWCCTACRGGAGGCRGCAGTGGGGAATATTGCRCAAT

GGGCGAAAGCCTGAKGCAGCGACGCCGCGTGAGGGATGACRGCCTTCGGGTTGTAAACCTCTTTCAGCASGGAAGAARCG

AAAGTGACRGTACCTGCMGAAGAAGCGCCGGCTAACTACGTGCCAGCAGCCGCGGTAATACGTAKGGCGCAAGCGTTGTC

CGGAATTATTGGGCGTAAAGAGCTCGTASGCGGCTTGTCACGTCGGAKGTGAAAGCCCGGGGCTTAACCCCGGGTCTGCA

TTCGATACGGGCTAGCTAGAGTGTGGYAGGGGAGATCGGAATTCCTGGTGTAGMGGWGAAATGCGCAGATAKCAKGAGGA

ACACCGGTGGCGAAGGCGGATCTCWGGGCCATTACTGACGCTGASGAGCGAAAGCGTGGGGAGCGAACAGGATTAGATAC

CSNGGTAGTCCACGCCGTAAACGTTGGGAACTAGGTGTTGGCGACATTCCACGTCGTCGGTGCCGCAGCTAACGCATTAA

GTTCCCCGCCTGGGGAGTACGGCCGCAAGGCTAAAACTCAAAGGAATTGACGGGGGCCCGCACAAGCAGCGGAGCATGTG

GCTTAATTCGACGCAACGCGAAGAACCTTACCAAGGCTTGACATATACCGGAAAGCATCAGAGATGGTGCCCCCCTTGTG

GTCGGTATACAGGTGGTGCATGGCTGTCGTCAGCTCGTGTCGTGAGATGTTGGGTTAAGTCCCGCAACGAGCGCAACCCT

TGTTCTGTGTTGCCAGCATGCCCTTCGGGNTGATGGGNACTCACNGGNNNCTGCCGGGGTCNNNTCGGAGGAAGNNNGGG

ACGACGTCAAGTCATCATGCCCNTTNNNTN

>D6_Bacillus_sp.

AAATCTGTCACCTTTAGGCGGCTGGCTCCTTACGGTTACCCCACCGACTTCGGGTGTTACTAACTCTCGT

GGTGTGACGGGCGGTGTGTACAAGGCCCGGGAACGTATTCACCGCGGCATGCTGATCCGCGATTACAAGC

GATTCCCGCTTCATGTAGGCGAGTTGCAGCCTACAATCCGAACTGAGAGTGGTTTTATGGGATTGGCTCT

ACCTCGCGGTTTTGCTGCCCTTTGTACCACCCATTGTAGCACGTGTGTAGCCCACGTCATAAGGGGGATG

ATGATTTGACGTCATCCCCCCCTTCCTCCGGTTTGTCACCGGCGGTCACCTTATAATGCCCAACTAAATG

CTGGCAACTAAAATCAAGGGTTGCGCTCGTTGCGGGACTTAACCCAACATCTCACGACACGAGCTGACGA

CAACCATGCACCACCTGTCACTCTTTCCCCCCAAAGGGAACGACCTATCTCTTTGGTTGTCAGAAGATGT

CAAGACCTGGTAAGGTTCTTCTCGTTGCTTCGAATTAAACCACATGCTCCACCCCTTGTGCGGGCCCCCC

TCAATTCCTTTGAGTTTCATCCTTGCGGCCCTACTCCCCACGCGGAGTGCTTAATGCGTTTGCTGCGCCA

CTAAAGGGCGGAAACCCTCTCACACTTAGCACTCATCTCTTACGGCGTGGACTACCAAGGTATCTAATCC

TGTTTGCTCCCCACGCTATCGCGCCTCACTGTCAGTTACAGGCCAGAGAGTCGCCTTCGCCACTGGTGTT

CCTCCACATCTCTACGCAATTCACCGCTACACGTGGAATTCCACTCTTCTCTCCTGCACTCAAGTCTCCC

AGTTTCCAATGACCCTCCCCGGTTGAGCCGAGGGCTTTCACATCACACTTAAGAGACCACCTGCGCGCGC

TTTACGCTCAATAAATTCCGGACAACACTTGCCACCTACCTATTACCGCGGCTGCTGGGACGTAATTAAC

CCTGGCTTTCTGGTTAGGTACCGTCCAGGTACCGGCCGTTACTCCCATACTTGTTCTTTCCTAACAAAAG

AGTTTTACGATCCGAAAACCTTCTTTCCTCAAGCGGGGTTGCTTCGTCCGACTTTCTTCCATGCGGAGAA

TACCTACTGCTGCCTCCGTAAGAATCTGGGGCGGGGCTCAATCCCAGTGTGGGCGACACCCTCTCCAGTC

GGCTACCACCCTCCCCTGGGGAGCCGTACCTCACCCATAAATTTAAGGGCCCCGGGCCCCCCCCTTAATG

AAAGCCAAACCACCT

>D7_Rhodococcus_sp.

CCTTCGACGGCTCCCTCCCACAAGGGGTTAGGCCACCGGCTTCGGGTGTTACCGACTTTC

ATGACGTGACGGGCGGTGTGTACAAGGCCCGGGAACGTATTCACCGCAGCGTTGCTGATC

TGCGATTACTAGCGACTCCGACTTCACGGGGTCGAGTTGCAGACCCCGATCCGAACTGAG

ACCAGCTTTAAGGGATTCGCTCCACCTCACGGTCTCGCAGCCCTCTGTACTGGCCATTGT

AGCATGTGTGAAGCCCTGGACATAAGGGGCATGATGACTTGACGTCGTCCCCACCTTCCT

TCCGAGTTGACCCCGGCAGTCTCTTACGAGTCCCCACCATTACGTGCTGGCAACATAAGA

CAAGGGTTGCGCTCGTTGCGGGACTTAACCCAACATCTCACGACACGAGCTGACGACAGC

CATGCACCACCTGTATACCGACCACAAGGGGGGCCGTATCTCTACGGCTTTCCGGTATAT

GTCAAACCCAGGTAAGGTTCTTCGCGTTGCATCGAATTAATCCACATGCTCCGCCGCTTG

TGCGGGCCCCCGTCAATTCCTTTGAGTTTTAGCCTTGCGGCCGTACTCCCCAGGCGGGGC

GCTTAATGCGTTAGCTACGGCACAGATCCCGTGGAAGGAACCCACACCTAGCGCCCACCG

TTTACGGCGTGGACTACCAGGGTATCTAATCCTGTTTGCTACCCACGCTTTCGTTCCTCA

GCGTCAGTTACTGCCCAGAGACCCGCCTTCGCCACCGGTGTTCCTCCTGATATCTGCGCA

TTTCACCGCTACACCAGGAATTCCAGTCTCCCCTGCAGTACTCAAGTCTGCCCGTATCGC

CTGCAGGCTCACAGTTGAGCTGTGAGTTTTCACAAACGACGCGACAAACCGCCTACGAAC

TCTTTACGCCCAGTAATTCCGGACAACGCTTGCACCCTACGTATTACCGCGGCTGCTGGC

ACGTAGTTAGCCGGTGCTTCTTCTGCAGGTACCGTCACTTTCGCTTCGTCCCTGCTGAAA

GAGGTTTACAACCCGAAGGCCGTCATCCCTCACGCGGCGTCGCTGCATCAGGCTTTCGCC

CATTGTGCAATATTCCCCACTGCTGCCTCCCGTAGGAGTCTGGGCCGTGTCTCAGTCCCA

GTGTGGCCGGTCACCCTCTCAGGTCGGCTACCCGTCGTCGCCTTGGTAGGCCATTACCCC

ACCAACAAGCTGATAGGCCGCGGGCCCATCCTGCACCAGTAAACCTTTCCACCCTCAGCC

ATGCAGCCGAAGGTCATATCCGGTATTAGACCCAGTTTCCCAGGCTTATCCCGAAGTGCA

GGGCAGATCACCCACGTGTTACTCACCCGTTCGCCGCTCGTGTACCCCGAAGGGCCTTAC

CGCTCGACTTGCATGTTTAA

>D8_Stenotrophomonas_rhizophila

TGCAAGTCGAACGGCAGCACAGTAAGAGCTTGCTCTTATGGGTGGCGAGTGGCGGACGGG

TGAGGAATACATCGGAATCTACCTTTTCGTGGGGGATAACGTAGGGAAACTTACGCTAAT

ACCGCATACGACCTTCGGGTGAAAGCAGGGGACCTTCGGGCCTTGCGCGGATAGATGAGC

CGATGTCGGATTAGCTAGTTGGCGGGGTAAAGGCCCACCAAGGCGACGATCCGTAGCTGG

TCTGAGAGGATGATCAGCCACACTGGAACTGAGACACGGTCCAGACTCCTACGGGAGGCA

GCAGTGGGGAATATTGGACAATGGGCGCAAGCCTGATCCAGCCATACCGCGTGGGTGAAG

AAGGCCTTCGGGTTGTAAAGCCCTTTTGTTGGGAAAGAAAAGCAGTCGATTAATACTCGG

TTGTTCTGACGGTACCCAAAGAATAAGCACCGGCTAACTTCGTGCCAGCAGCCGCGGTAA

TACGAAGGGTGCAAGCGTTACTCGGAATTACTGGGCGTAAAGCGTGCGTAGGTGGTTGTT

TAAGTCTGTTGTGAAAGCCCTGGGCTCAACCTGGGAATTGCAGTGGATACTGGGCGACTA

GAGTGTGGTAGAGGGTAGTGGAATTCCCGGTGTAGCAGTGAAATGCGTAGAGATCGGGAG

GAACATCCATGGCGAAGGCAGCTACCTGGACCAACACTGACACTGAGGCACGAAAGCGTG

GGGAGCAAACAGGATTAGATACCCTGGTAGTCCACGCCCTAAACGATGCGAACTGGATGT

TGGGTGCAATTTGGCACGCAGTATCGAAGCTAACGCGTTAAGTTCGCCGCCTGGGGAGTA

CGGTCGCAAGACTGAAACTCAAAGGAATTGACGGGGGCCCGCACAAGCGGTGGAGTAAGT

GGGTTTAATTCGATGCAACGCGAAGAACCTTACCTGGTCTTGACATGTCGAGAACTTTCC

AGAGAATGGATTGGTGCCTTCGGGAACTCGAACACAGGTGCTGCATGGCTGTCGTCAGCT

CGTGTCGTGAAATGTTGGGTTAAGTCCCGCAACGAGCGCAACCCTTGTCCTTAGTTGCCA

GCACGTAATGGTGGGAACTCTAAGGAGACCGCCGGTGACAAACCGGAGGAAGGTGGGGAT

GACGTCAAGTCATCATGGCCCTTACGACCAGGGCTACACACGTACTACAATGGTAGGGAC

AGAGGGCTGCAAACCCGCGAGGGCAAGCCAATCCCAGAAACCCTATCTCAGTCCGGATTG

GAGTCTGCAACTCGACTCCATGAAGTCGGAATCGCTAGTAATCGCAGATCAGCATTGCTG

CGGTGAATACGTTCCCGGGCCTTGTACACACCGCCCGTCACACCATGGGAGTTTGTTGCA

CCAGAAGCAGGTAGCTTAACCTTCGGGAGGGCGCTGCCA

>D9_Rahnella_aquatilis

ATGCAGTCGAGCGGCAGCGGGAAAGTAGCTTGCTACTTTGCCGGCGAGCGGCGGACGGGT

GAGTAATGTCTGGGAAACTGCCTGATGGAGGGGGATAACTACTGGAAACGGTAGCTAATA

CCGCATGACCTCGAAAGAGCAAAGTGGGGGATCTTCGGACCTCACGCCATCGGATGTGCC

CAGATGGGATTAGCTAGTAGGTGAGGTAATGGCTCACCTAGGCGACGATCCCTAGCTGGT

CTGAGAGGATGACCAGCCACACTGGAACTGAGACACGGTCCAGACTCCTACGGGAGGCAG

CAGTGGGGAATATTGCACAATGGGCGCAAGCCTGATGCAGCCATGCCGCGTGTGTGAAGA

AGGCCTTAGGGTTGTAAAGCACTTTCAGCGAGGAGGAAGGCATCATACTTAATACGTGTG

GTGATTGACGTTACTCGCAGAAGAAGCACCGGCTAACTCCGTGCCAGCAGCCGCGGTAAT

ACGGAGGGTGCAAGCGTTAATCGGAATTACTGGGCGTAAAGCGCACGCAGGCGGTTTGTT

AAGTCAGATGTGAAATCCCCGCGCTTAACGTGGGAACTGCATTTGAAACTGGCAAGCTAG

AGTCTTGTAGAGGGGGGTAGAATTCCAGGTGTAGCGGTGAAATGCGTAGAGATCTGGAGG

AATACCGGTGGCGAAGGCGGCCCCCTGGACAAAGACTGACGCTCAGGTGCGAAAGCGTGG

GGAGCAAACAGGATTAGATACCCTGGTAGTCCACGCTGTAAACGATGTCGACTTGGGAGG

TTGTGCCCTTGAGGCGTGGCTTCCGGAGCTAACGCGTTAAGTCGACCGCCTGGGGGAGTA

CGGCCGCAAGGTTAAAACTCAAATGAAATTGACGGGGGCCCGCACAAGCGGTGGAACATG

GTGGTTTAATTCGATGCAACGCGAAGAACCTTACCTACTCTTGACATCCACGGAATTCGC

CAGAGATGGCTTAGTGCCTTCGGGAACCGTGAGACAGGTGCTGGCATGGCTGTCGTCAGC

TCGTGTTGTGAAAAGGTTTGGGTTAAGTCCCGCAACGAGCGCAACCCTTATCCTTTGTTG

CCAGCACGTAAAGGGTGGGAACTCAAAGGAGACTGCCGGTGATAAACCGGAGGAAGGTGG

GGATGACGTCAAGTCATCATGGCCCTTACGAGTAGGGCTACACACGTGCTACAATGGCAT

ATACAAAGAGAAGCAAACTCGCGAGAGCAAGCGGACCTCATAAAGTATGTCGTAGTCCGG

ATTGGAGTCTGCAACTCGACTCCATGAAGTCGGAATCGCTAGTAATCGTAGATCAGAATG

CTACGGTGAATACGTTCCCGGGCCTTGTACACACCGCCCGTCACACCATGGGAGTGGGTT

GCAAAAGAAGTAGGTAGCTTAACCTTCGGGAGGGCGCT

>D10_Stenotrophomonas_rhizophila

ATGCAAGTCGAACGGCAGCACAGTAAGAGCTTGCTCTTATGGGTGGCGAGTGGCGGACGG

GTGAGGAATACATCGGAATCTACCTTTTCGTGGGGGATAACGTAGGGAAACTTACGCTAA

TACCGCATACGACCTTCGGGTGAAAGCAGGGGACCTTCGGGCCTTGCGCGGATAGATGAG

CCGATGTCGGATTAGCTAGTTGGCGGGGTAAAGGCCCACCAAGGCGACGATCCGTAGCTG

GTCTGAGAGGATGATCAGCCACACTGGAACTGAGACACGGTCCAGACTCCTACGGGAGGC

AGCAGTGGGGAATATTGGACAATGGGCGCAAGCCTGATCCAGCCATACCGCGTGGGTGAA

GAAGGCCTTCGGGTTGTAAAGCCCTTTTGTTGGGAAAGAAAAGCAGTCGATTAATACTCG

GTTGTTCTGACGGTACCCAAAGAATAAGCACCGGCTAACTTCGTGCCAGCAGCCGCGGTA

ATACGAAGGGTGCAAGCGTTACTCGGAATTACTGGGCGTAAAGCGTGCGTAGGTGGTTGT

TTAAGTCTGTTGTGAAAGCCCTGGGCTCAACCTGGGAATTGCAGTGGATACTGGGCGACT

AGAGTGTGGTAGAGGGTAGTGGAATTCCCGGTGTAGCAGTGAAATGCGTAGAGATCGGGA

GGAACATCCATGGCGAAGGCAGCTACCTGGACCAACACTGACACTGAGGCACGAAAGCGT

GGGGAGCAAACAGGATTAGATACCCTGGTAGTCCACGCCCTAAACGATGCGAACTGGATG

TTGGGTGCAATTTGGCACGCAGTATCGAAGCTAACGCGTTAAGTTCGCCGCCTGGGGAGT

ACGGTCGCAAGACTGAAACTCAAAGGAATTGACGGGGGCCCGCACAAGCGGTGGAGTATG

TGGTTTAATTCGATGCAACGCGAAGAACCTTACCTGGTCTTGACATGTCGAGAACTTTCC

AGAGATGGATTGGTGCCTTCGGGAACTCGAACACAGGTGCTGCATGGCTGTCGTCAGCTC

GTGTCGTGAGATGGTTGGGTTAAGTCCCGCAACGAGCGCAACCCTTGTCCTTAGTTGCCA

GCACGTAATGGTGGGAACTCTAAGGAGACCGCCGGTGACAAACCGAGAAAAAGGGGGGGA

TGACGTCAAGTCATCATGGCCCTTACGACCAGGGCTACACACGTACTACAATGGTAGGGA

CAGAGGGCTGCAAACCCGCGAGGGCAAGCCAATCCCAGAAACCCTATCTCAGTCCGGATT

GGAGTCTGCAACTCGACTCCATGAAGTCGGAATCGCTAGTAATCGCAGATCAGCATTGCT

GCGGTGAATACGTTCCCGGGCCTTGTACACACCGCCCGTCACACCATGGGAGTTTGTTGC

ACCAGAAGCAGGTAGCTTAACCTTCGGGAGGGCGCTGCC

>D11_Pseudomonas_sp.

TGCAAGTCGAGCGGTAGAGAGAAGCTTGCTTCTCTTGAGAGCGGCGGACGGGTGAGTAAT

GCCTAGGAATCTGCCTGGTAGTGGGGGATAACGTTCGGAAACGGACGCTAATACCGCATA

CGTCCTACGGGAGAAAGCAGGGGACCTTCGGGCCTTGCGCTATCAGATGAGCCTAGGTCG

GATTAGCTAGTTGGTGAGGTAATGGCTCACCAAGGCGACGATCCGTAACTGGTCTGAGAG

GATGATCAGTCACACTGGAACTGAGACACGGTCCAGACTCCTACGGGAGGCAGCAGTGGG

GAATATTGGACAATGGGCGAAAGCCTGATCCAGCCATGCCGCGTGTGTGAAGAAGGTCTT

CGGATTGTAAAGCACTTTAAGTTGGGAGGAAGGGCAGTAAATTAATACTTTGCTGTTTTG

ACGTTACCGACAGAATAAGCACCGGCTAACTCTGTGCCAGCAGCCGCGGTAATACAGAGG

GTGCAAGCGTTAATCGGAATTACTGGGCGTAAAGCGCGCGTAGGTGGTTTGTTAAGTTGG

ATGTGAAATCCCCGGGCTCAACCTGGGAACTGCATTCAAAACTGACTGACTAGAGTATGG

TAGAGGGGTGGGTGGAAATTTCCTGTGGTAGCGGTGAAATGCGTAGATATAGGAAGGAAC

ACCAGTGGCGAAGGCGACCACCTGGACTAATACTGACACTGAGGTGCGAAAGCGTGGGGA

GCAAACAGGATTAGATACCCTGGTAGTCCACGCCGTAAACGATGTCAACTAGCCGTTGGA

AGCCTTGAGCTTTTAGTGGCGCAGCTAACGCATTAAGTTGACCGCCTGGGGGAGTACGGC

CGCAAGGTTAAAACTCAAATGAAATTGACGGGGGGCCCGCACAAGCGGTGGAGCATGTGG

GTTTAATTTCGAAAGCAACGCGAAAGAACCTTACCAGGCCTTGACATCCAATGAACTTTC

TAGAGATAGATTGGTGCCTTCGGGAACATTGAGACAGGTGCTGCATGGCTGTCGTCAGCT

CGTGTCGTGAGATGTTGGGTTAAGTCCCGTAACGAGCGCAACCCTTGTCCTTAGTTACCA

GCACGTCATGGTGGGCACTCTAAGGAGACTGCCGGTGACAAACCGGAGGAAGGTGGGGAT

GACGTCAAGTCATCATGGCCCTTACGGCCTGGGCTACACACGTGCTACAATGGTCGGTAC

AGAGGGTTGCCAAGCCGCGAGGTGGAGCTAATCCCACAAAACCGATCGTAGTCCGGATCG

CAGTCTGCAACTCGACTGCGTGAAGTCGGAATCGCTAGTAATCGCGAATCAGAATGTCGC

GGTGAATACGTTCCCGGGCCTTGTACACACCGCCCGTCACACCATGGGAGTGGGTTGCAC

CAGAAGTAGCTAGTCTAACCTTCGGGAGGACGGTAC

>D12_Pseudomonas_sp.

ATGCAAGTCGAGCGGTAGAGAGAAGCTTGCTTCTCTTGAGAGCGGCGGACGGGTGAGTAA

TGCCTAGGAATCTGCCTGGTAGTGGGGGATAACGTTCGGAAACGGACGCTAATACCGCAT

ACGTCCTACGGGAGAAAGCAGGGGACCTTCGGGCCTTGCGCTATCAGATGAGCCTAGGTC

GGATTAGCTAGTTGGTGAGGTAATGGCTCACCAAGGCGACGATCCGTAACTGGTCTGAGA

GGATGATCAGTCACACTGGAACTGAGACACGGTCCAGACTCCTACGGGAGGCAGCAGTGG

GGAATATTGGACAATGGGCGAAAGCCTGATCCAGCCATGCCGCGTGTGTGAAGAAGGTCT

TCGGATTGTAAAGCACTTTAAGTTGGGAGGAAGGGCAGTAAATTAATACTTTGCTGTTTT

GACGTTACCGACAGAATAAGCACCGGCTAACTCTGTGCCAGCAGCCGCGGTAATACAGAG

GGTGCAAGCGTTAATCGGAATTACTGGGCGTAAAGCGCGCGTAGGTGGTTTGTTAAGTTG

GATGTGAAATCCCCGGGCTCAACCTGGGAACTGCATTCAAAACTGACTGACTAGAGTATG

GTAGAGGGTGGGTGGAATTTCCTGTGTAGCGGTGAAATGCGTAGATATAGGAAGGAACAC

CAGTGGCGAAGGCGACCACCTGGACTAATACTGACACTGAGGTGCGAAAGCGTGGGGAGC

AAACAGGATTAGATACCCTGGTAGTCCACGCCGTAAACGATGTCAACTAGCCGTTGGAAG

CCTTGAGCTTTTAGTGGCGCAGCTAACGCATTAAGTTGACCGCCTGGGGAGTACGGCCGC

AAGGTTAAAACTCAAATGAATTGACGGGGGCCCGCACAAGCGGTGGAGCATGTGGGTTTA

ATTCGAAGCAACGCGAAGAACCTTACCAGGCCTTGACATCCAATGAACTTTCTAGAGAAT

AGATTGGTGCCTTCGGGAACATTGAGACAGGTGCTGCATGGCTGTCGTCAGCTCGTGTCG

TGAGAAGGTTGGGTTAAGTCCCGTAACGAGCGCAACCCTTGTCCTTAGTTACCAGCACGT

CATGGTGGGCACTCTAAGGAGACTGCCGGTGACAAACCGGAGGAAGGTGGGGATGACGTC

AAGTCATCATGGCCCTTACGGCCTGGGCTACACACGTGCTACAATGGTCGGTACAGAGGG

TTGCCAAGCCGCGAGGTGGAGCTAATCCCACAAAACCGATCGTAGTCCGGATCGCAGTCT

GCAACTCGACTGCGTGAAGTCGGAATCGCTAGTAATCGCGAATCAGAATGTCGCGGTGAA

TACGTTCCCGGGCCTTGTACACACCGCCCGTCACACCATGGGAGTGGGTTGCACCAGAAG

TAGCTAGTCTAACCTTCGGGAGGACGGTACC

>E1_Pseudomonas_fluorescens

ATGCAGTCGAGCGGTAGAGAGAAGCTTGCTTCTCTTGAGAGCGGCGGACGGGTGAGTAAT

GCCTAGGAATCTGCCTGGTAGTGGGGGATAACGTTCGGAAACGAACGCTAATACCGCATA

CGTCCTACGGGAGAAAGCAGGGGACCTTCGGGCCTTGCGCTATCAGATGAGCCTAGGTCG

GATTAGCTAGTTGGTGAGGTAATGGCTCACCAAGGCGACGATCCGTAACTGGTCTGAGAG

GATGATCAGTCACACTGGAACTGAGACACGGTCCAGACTCCTACGGGAGGCAGCAGTGGG

GAATATTGGACAATGGGCGAAAGCCTGATCCAGCCATGCCGCGTGTGTGAAGAAGGTCTT

CGGATTGTAAAGCACTTTAAGTTGGGAGGAAGGGCATTAACCTAATACGTTAGTGTTTTG

ACGTTACCGACAGAATAAGCACCGGCTAACTCTGTGCCAGCAGCCGCGGTAATACAGAGG

GTGCAAGCGTTAATCGGAATTACTGGGCGTAAAGCGCGCGTAGGTGGTTTGTTAAGTTGG

ATGTGAAATCCCCGGGCTCAACCTGGGAACTGCATTCAAAACTGACTGACTAGAGTATGG

TAGAGGGTGGGTGGAATTTCCTGTGTAGCGGTGAAATGCGTAGATATAGGAAGGAACACC

AGTGGCGAAGGCGACCACCTGGACTAATACTGACACTGAGGTGCGAAAGCGTGGGGAGCA

AACAGGATTAGATACCCTGGTAGTCCACGCCGTAAACGATGTCAACTAGCCGTTGGAAGC

CTTGAGCTTTTAGTGGCGCAGCTAACGCATTAAGTTGACCGCCTGGGGAGTACGGCCGCA

AGGTTAAAACTCAAATGAATTGACGGGGGCCCGCACAAGCGGTGGAGCATGTGGTTTAAT

TCGAAGCAACGCGAAGAACCTTACCAGGCCTTGACATCCAATGAACTTTCTAGAGATAGA

TTGGTGCCTTCGGCAACATTGAGACAGGTGCTGCATGGCTGTCGTCAGCTCGTGTCGTGA

GATGTTGGGTTAAGTCACGTAACGAACGCAACCCTTGTCCTTAATTACCAGCACGTAATG

GTGGGCACTCTAAGGAGACTGCCGGTGACAAACCGGAGGAAGGTGGGGATGACGTCAAGT

CATCATGGCCCTTACGGCCTGGGCTACACACGTGCTACAATGGTCGGTACAGAGGGTTGC

CAAGCCGCGAGGTGGAGCTAATCCCATAAAACCGATCGTAGTCCGGATCGCAGTCTGCAA

CTCGACTGCGTGAAGTCGGAATCGCTAGTAATCGCGAATCAGAATGTCGCGGTGAATACG

TTCCCGGGCCTTGTACACACCGCCCGTCACACCATGGGAGTGGGTTGCACCAGAAGTAGC

TAGTCTAACCTTCGGGAGGACGGT

>E2_Stenotrophomonas_rhizophila

ACACATGCAAGTCGAACGGCAGCACAGTAAGAGCTTGCTCTTATGGGTGGCGAGTGGCGG

ACGGGTGAGGAATACATCGGAATCTACCTTTTCGTGGGGGATAACGTAGGGAAACTTACG

CTAATACCGCATACGACCTTCGGGTGAAAGCAGGGGACCTTCGGGCCTTGCGCGGATAGA

TGAGCCGATGTCGGATTAGCTAGTTGGCGGGGTAAAGGCCCACCAAGGCGACGATCCGTA

GCTGGTCTGAGAGGATGATCAGCCACACTGGAACTGAGACACGGTCCAGACTCCTACGGG

AGGCAGCAGTGGGGAATATTGGACAATGGGCGCAAGCCTGATCCAGCCATACCGCGTGGG

TGAAGAAGGCCTTCGGGTTGTAAAGCCCTTTTGTTGGGAAAGAAAAGCAGTCGATTAATA

CTCGGTTGTTCTGACGGTACCCAAAGAATAAGCACCGGCTAACTTCGTGCCAGCAGCCGC

GGTAATACGAAGGGTGCAAGCGTTACTCGGAATTACTGGGCGTAAAGCGTGCGTAGGTGG

TTGTTTAAGTCTGTTGTGAAAGCCCTGGGCTCAACCTGGGAATTGCAGTGGATACTGGGC

GACTAGAGTGTGGTAGAGGGTAGTGGAATTCCCGGTGTAGCAGTGAAATGCGTAGAGATC

GGGAGGAACATCCATGGCGAAGGCAGCTACCTGGACCAACACTGACACTGAGGCACGAAA

GCGTGGGGAGCAAACAGGATTAGATACCCTGGTAGTCCACGCCCTAAACGATGCGAACTG

GATGTTGGGTGCAATTTGGCACGCAGTATCGAAGCTAACGCGTTAAGTTCGCCGCCTGGG

GAGTACGGTCGCAAGACTGAAACTCAAAGGAATTGACGGGGGCCCGCACAAGCGGTGGAA

TATGTTGGTTTAATTCGATGCAACGCGAAGAACCTTACCTGGTCTTGACATGTCGAGAAC

TTTCAAAAGATGGATTGGTGCCTTCGGGAACTCGAACACAGGTGCTGCATGGCTGTCGTC

AGCTCGTGTCGTGAGAAGTTTGGGTTAAGTCCCGCAACGAGCGCAACCCTTGTCCTTAGT

TGCCAGCACGTAATGGTGGGAACTCTAAGGAGACCGCCGGTGACAAACCGGAGGAAGGTG

GGGATGACGTCAAGTCATCATGGCCCTTACGACCAGGGCTACACACGTACTACAATGGTA

GGGACAGAGGGCTGCAAACCCGCGAGGGCAAGCCAATCCCAGAAACCCTATCTCAGTCCG

GATTGGAGTCTGCAACTCGACTCCATGAAGTCGGAATCGCTAGTAATCGCAGATCAGCAT

TGCTGCGGTGAATACGTTCCCGGGCCTTGTACACACCGCCCGTCACACCATGGGAGTTTG

TTGCACCAGAAGCAGGTAGCTTAACCTTCGGGAGGGCGCTGCC

>E3_Niallia_circulans

TGCAAGTCGAGCGGACTTTAAAGCTTGCTTTTAAGTTAGCGGCGGACGGGTGAGTAACAC

GTGGGCAACCTGCCTGTAAGACTGGGATAACTTCGGGAAACCGGAGCTAATACCGGATAA

TCCTTTTCTACACATGTAGAAAAGCTGAAAGACGGTTTACGCTGTCACTTACAGATGGGC

CCGCGGCGCATTAGCTAGTTGGTGAGGTAACGGCTCACCAAGGCAACGATGCGTAGCCGA

CCTGAGAGGGTGATCGGCCACACTGGGACTGAGACACGGCCCAGACTCCTACGGGAGGCA

GCAGTAGGGAATTCTTCCGCAATGGACGAAAGTCTGACGGAGCAACGCCGCCTGAGTGAT

GAAGGTTTTCGGATCGTAAAACTCTGTTGTTAGGGAAGAACAAGTACAAGAGTAACTGCT

TGTACCTTGACGGTACCTAACCAGAAAGCCACGGCTAACTACGTGCCAGCAGCCGCGGTA

ATACGTAGGTGGCAAGCGTTGTCCGGAATTATTGGGCGTAAAGCGCGCGCAGGCGGTCCT

TTAAGTCTGATGTGAAAGCCCACGGCTCAACCGTGGAGGGTCATTGGAAACTGGGGGACT

TGAGTGCAGAAGAGAAGAGTGGAATTCCACGTGTAGCGGTGAAATGCGTAGAGATGTGGA

GGAACACCAGTGGCGAAGGCGACTCTTTGGTCTGTAACTGACGCTGAGGCGCGAAAGCGT

GGGGAGCAAACAGGATTAGATACCCTGGTAGTCCACGCCGTAAACGATGAGTGCTAAGTG

TTAGAGGGTTTCCGCCCTTTAGTGCTGCAGCAAACGCATTAAGCACTCCGCCTGGGGAGT

ACGGCCGCAAGGCTGAAACTCAAAGGAATTGACGGGGGCCCGCACAAGCGGTGGAGCATG

TGGTTTAATTCGAAGCAACGCGAAGAACCTTACCAGGTCTTGACATCCTCTGACACTCCT

AGAGATAGGACGTTCCCCTTCGGGGGACAGAGTGACAGGTGGTGCATGGTTGTCGTCAGC

TCGTGTCGTGAGATGTTGGGTTAAGTCCCGCAACGAGCGCAACCCTTGATCTTAGTTGCC

AGCATTAAGTTGGGCACTCTAAGGTGACTGCCGGTGACAAACCGGAGGAAGGTGGGGATG

ACGTCAAATCATCATGCCCCTTATGACCTGGGCTACACACGTGCTACAATGGATGGTACA

AAGGGCAGCAAAGCGGTGACGCCTAGCAAATCCCATAAAACCATTCTCAGTTCGGATTGT

AGGCTGCAACTCGCCTACATGAAGCGGGAATCGCTAGTAATCGCGGATCAGCATGCCGCG

GTGAATACGTTCCCGGGCCTTGTACACACCGCCCGTCACACCACGAGAGTTTGTAACACC

CGAAGTCGGTGGGGTAACCTTTGGAGCCCAGCCGCCTA

>E4_Pseudomonas_sp.

GGTACCGTCCTCCCGAAGGTTAGACTAGCTACTTCTGGTGCAACCCACTCCCATGGTGTG

ACGGGCGGTGTGTACAAGGCCCGGGAACGTATTCACCGCGACATTCTGATTCGCGATTAC

TAGCGATTCCGACTTCACGCAGTCGAGTTGCAGACTGCGATCCGGACTACGATCGGTTTT

ATGGGATTAGCTCCACCTCGCGGCTTGGCAACCCTTTGTACCGACCATTGTAGCACGTGT

GTAGCCCAGGCCGTAAGGGCCATGATGACTTGACGTCATCCCCACCTTCCTCCGGTTTGT

CACCGGCAGTCTCCTTAGAGTGCCCACCATAACGTGCTGGTAACTAAGGACAAGGGTTGC

GCTCGTTACGGGACTTAACCCAACATCTCACGACACGAGCTGACGACAGCCATGCAGCAC

CTGTCTCAATGCTACCGAAGGCACCAATCCATCTCTGGAAAGTTCATTGGATGTCAAGGC

CTGGTAAGGTTCTTCGCGTTGCTTCGAATTAAACCACATGCTCCACCGCTTGTGCGGGCC

CCCGTCAATTCATCTGAGTCTTAACCTTGCGGCCGTACTCCCCAGGCGGTCAACTTAATG

CGTTAGCTGCGCCACTAAGAGCTCAAGGCTCCCAACGGCTAGTTGACATCGTGTACGGCG

TGGACTACCAGGGTATCTAATCCTGTTTGCTCCCCACGCTTTCGCACCTCAGTGTCAGTA

TCAGTCCAGGTGGTCGCCTTCGCCACTGGTGTTCCTTCCTATATCTACGCATTTCACCGC

TACACAGGAAATTCCACCCACCCTCTACCATACTCTAGCTTGCCAGTTTTGGATGCAGTT

CCCAGGTTGAGCCCGGGGATTTCACATCCAACTTAACAAACCACCTACGCGCGCTTTACG

CCCAGTAATTCCGATTAACGCTTGCACCCTCTGTATTACCGCGGCTGCTGGCACAGAGTT

AGCCGGTGCTTATTCTGTCGGTAACGTCAAAATTGCAGAGTATTAATCTACAACCCTTCC

TCCCAACTTAAAGTGCTTTACAATCCGAAGACCTTCTTCACACACGCGGCAGGGCTGGAT

CAGGCTTTCGCCCATTGTCCAATATTCCCCACTGCTGCCTCCCGTAGGAATCTGGACCGT

GTCTCAGTTCCAGTGTGACTGATCATCCTCTCAGACCAGTTACGGATCGTCGCCTTGGTG

AGCCATTACCTCACCAACTAGCTAATCCGACCTAGGCTCATCTGATAGCGCAAGGCCCGA

AGGTCCCCTGCTTTCTCCCGTAGGACGTATGCGGTATTAGCGTTCCTTTCGAAACGTTGT

CCCCCACTACCAGGCAGATTCCTAGGCATTACTCACCCGTCCGCCGCTGAATCCAGGAGC

AAGCTCCTTTCATCCGCTCGACTTGCAT

>E5_Pseudomonas_sp.

GCTACTTCTGGTGCAACCCACTCCCATGGTGTGACGGGCGGTGTGTACAAGGCCCGGGAA

CGTATTCACCGCGACATTCTGATTCGCGATTACTAGCGATTCCGACTTCACGCAGTCGAG

TTGCAGACTGCGATCCGGACTACGATCGGTTTTATGGGATTAGCTCCACCTCGCGGCTTG

GCAACCCTTTGTACCGACCATTGTAGCACGTGTGTAGCCCAGGCCGTAAGGGCCATGATG

ACTTGACGTCATCCCCACCTTCCTCCGGTTTGTCACCGGCAGTCTCCTTAGAGTGCCCAC

CATAACGTGCTGGTAACTAAGGACAAGGGTTGCGCTCGTTACGGGACTTAACCCAACATC

TCACGACACGAGCTGACGACAGCCCATGCCAGCACCTGTCTTCAATGCTTCCCGAAGGCA

CCCAATCCATCTTCTGGAAAGTTCATTGGATGTCAAGGCCTGGTAAGGTTTCTTCGCGTT

GCTTCGAATTAAACCACATGCTCCACCGCTTGTGCGGGCCCCCCGTCAATTTCATTTGAG

TTTTAACCTTGCGGCCGTACTCCCCCAGGCGGTCAACTTAATGCGTTAGCTGCGCCACTA

AGAGCTCAAGGCTCCCAACGGCTAGTTGACATCGTTTACGGCGTGGACTACCAGGGTATC

TAATCCTGTTTGCTCCCCACGCTTTCGCACCTCAGTGTCAGTATCAGTCCAGGTGGTCGC

CTTCGCCACTGGTGTTCCTTCCTATATCTACGCATTTCACCGCTACACAGGAAATTTCCA

CCCACCCTCTACCATACTCTAGCTTGCCAGTTTTGGATGCAGTTCCCAGGTTGAGCCCGG

GGATTTCACATCCAACTTAACAAACCACCTACGCGCGCTTTACGCCCAGTAATTCCGATT

AACGCTTGCACCCTCTGTATTACCGCGGCTGCTGGCACAGAGTTAGCCGGTGCTTATTCT

GTCGGTAACGTCAAAATTGCAGAGTATTAATCTACAACCCTTCCTCCCAACTTAAAGTGC

TTTACAATCCGAAGACCTTCTTCACACACGCGGCAGGGCTGGATCAGGCTTTCGCCCATT

GTCCAATATTCCCCACTGCTGCCTCCCGTAGGAATCTGAACCGTGTCTCAGTTCCAGTGT

GACTGATCATCCTCTCAGACCAGTTACGGATCGTCGCCTTGGTGAGCCATTACCTCACCA

ACTAGCTAATCCGACCTAGGCTCATCTGATAGCGCAAGGCCCGAAGGTCCCCTGCTTTCT

CCCGTAGGACGTATGCGGTATTAGCGTTCCTTTCGAAACGTTGTCCCCCACTACCAGGCA

GATTCCTAGGCATTACTCACCCGTCCGCCGCTGAATCCAGGAGCAAGCTCCTTTCATCCG

CTCGACTTGCA

>E6_Pseudomonas_poae

TGCAAGTCGAGCGGTAGAGAGAAGCTTGCTTCTCTTGAGAGCGGCGGACGGGTGAGTAAT

GCCTAGGAATCTGCCTGGTAGTGGGGGATAACGTTCGGAAACGAACGCTAATACCGCATA

CGTCCTACGGGAGAAAGCAGGGGACCTTCGGGCCTTGCGCTATCAGATGAGCCTAGGTCG

GATTAGCTAGTTGGTGAGGTAATGGCTCACCAAGGCGACGATCCGTAACTGGTCTGAGAG

GATGATCAGTCACACTGGAACTGAGACACGGTCCAGACTCCTACGGGAGGCAGCAGTGGG

GAATATTGGACAATGGGCGAAAGCCTGATCCAGCCATGCCGCGTGTGTGAAGAAGGTCTT

CGGATTGTAAAGCACTTTAAGTTGGGAGGAAGGGCAGTTACCTAATACGTGATTGTTTTG

ACGTTACCGACAGAATAAGCACCGGCTAACTCTGTGCCAGCAGCCGCGGTAATACAGAGG

GTGCAAGCGTTAATCGGAATTACTGGGCGTAAAGCGCGCGTAGGTGGTTTGTTAAGTTGG

ATGTGAAATCCCCGGGCTCAACCTGGGAACTGCATTCAAAACTGACTGACTAGAGTATGG

TAGAGGGTGGTGGAATTTCCTGTGTAGCGGTGAAATGCGTAGATATAGGAAGGAACACCA

GTGGCGAAGGCGACCACCTGGACTAATACTGACACTGAGGTGCGAAAGCGTGGGGAGCAA

ACAGGATTAGATACCCTGGTAGTCCACGCCGTAAACGATGTCAACTAGCCGTTGGAAGCC

TTGAGCTTTTAGTGGCGCAGCTAACGCATTAAGTTGACCGCCTGGGGAGTACGGCCGCAA

GGTTAAAACTCAAATGAATTGACGGGGGCCCGCACAAGCGGTGGAGCATGTGGTTTAATT

CGAAGCAACGCGAAGAACCTTACCAGGCCTTGACATCCAATGAACTTTCTAGAGATAGAT

TGGTGCCTTCGGGAACATTGAGACAGGTGCTGCATGGCTGTCGTCAGCTCGTGTCGTGAG

ATGTTGGGTTAAGTCCCGTAACGAGCGCAACCCTTGTCCTTAGTTACCAGCACGTAATGG

TGGGCACTCTAAGGAGACTGCCGGTGACAAACCGGAGGAAGGTGGGGATGACGTCAAGTC

ATCATGGCCCTTACGGCCTGGGCTACACACGTGCTACAATGGTCGGTACAGAGGGTTGCC

AAGCCGCGAGGTGGAGCTAATCCCATAAAACCGATCGTAGTCCGGATCGCAGTCTGCAAC

TCGACTGCGTGAAGTCGGAATCGCTAGTAATCGCGAATCAGAATGTCGCGGTGAATACGT

TCCCGGGCCTTGTACACACCGCCCGTCACACCATGGGAGTGGGTTGCACCAGAAGTAGCT

AGTCTAACCTTCGGGAGGACGGTA

>E7_Rhodococcus_qingshengii

AAGTCGAGCGGTAAGGCCTTTCGGGGTACACGAGCGCGAACGGGTGAGTAACACGTGGGT

GATCTGCCCTGCACTTCGGGATAAGCCTGGGTAAACTGGGTCTAATACCGGATATGACCT

CCTATCGCATGGTGGGTGGTGGAAAGATTTATCGGTGCAGGATGGGCCCGCGGCCTATCA

GCTTGTTGGTGGGGTAATGGCCTACCAAGGCGACGACGGGTAGCCGACCTGAGAGGGTGA

CCGGCCACACTGGGACTGAGACACGGCCCAGACTCCTACGGGAGGCAGCAGTGGGGAATA

TTGCACAATGGGCGAAAGCCTGATGCAGCGACGCCGCGTGAGGGATGACGGCCTTCGGGT

TGTAAACCTCTTTCAGCAGGGACGAAGCGCAAGTGACGGTACCTGCAGAAGAAGCACCGG

CTAACTACGTACCAGCAGCCGCGGTAATACGTAGGGTGCAAGCGTTGTCCGGAATTACTG

GGCGTAAAGAGTTCGTAGGCGGTTTGTCGCGTCGTTTGTGAAAACCAGCAGCTCAACTGC

TGGCTTGCAGGCGATACGGGCAGACTTGAGTACTGCAGGGGAGACTGGAATTCCTGGTGT

AGCGGTGAAATGCGCAGATATCAGGAGGAACACCGGTGGCGAAGGCGGGTCTCTGGGCAG

TAACTGACGCTGAGGAACGAAAGCGTGGGTAGCGAACAGGATTAGATACCCTGGTAGTCC

ACGCCGTAAACGGTGGGCGCTAGGTGTGGGTTCCTTCCACGGAATCCGTGCCGTAGCTAA

CGCATTAAGCGCCCCGCCTGGGGAGTACGGCCGCAAGGCTAAAACTCAAAGGAATTGACG

GGGGCCCGCACAAGCGGCGGAGCATGTGGATGAATTCGATGCAACGCGAAGAACCTTACC

TGGGTTTGACATATACCGGAAAGCTGCAGAGATGTGGCCCCCCTTGTGGTCGGTATACAG

GTGGTGCATGGCTGTCGTCAGCTCGTGTCGTGAGATGTTGGGTTAAGTCCCGCAACGAGC

GCAACCCCTATCTTATGTTGCCAGCACGTTATGCTGGGGACTCGTAAGAGACTGCCGGGG

TCAACTCGGAGGAAGGTGGGGACGAACTTCAAGTCATCATGCCCCTTATGTCCAGGGCTT

CCCCCATGCTACAATGGCCAGTACAAAGGGCTGCAAAACCGTGGGGGGGAGCGAATCCCT

TAAAGCTGGCTTCAGTTCGGATCGGGG

>E8_Stenotrophomonas_sp.

CAGCGCCCTCCCGAAGGTTAAGCTACCTGCTTCTGGTGCAACAAACTCCCATGGTGTGAC

GGGCGGTGTGTACAAGGCCCGGGAACGTATTCACCGCAGCAATGCTGATCTGCGATTACT

AGCGATTCCGACTTCATGGAGTCGAGTTGCAGACTCCAATCCGGACTGAGATAGGGTTTC

TGGGATTGGCTTGCCCTCGCGGGTTTGCAGCCCTCTGTCCCTACCATTGTAGTACGTGTG

TAGCCCTGGTCGTAAGGGCCATGATGACTTGACGTCATCCCCACCTTCCTCCGGTTTGTC

ACCGGCGGTCTCCTTAGAGTTCCCACCATTACGTGCTGGCAACTAAGGACAAGGGTTGCG

CTCGTTGCGGGACTTAACCCAACATCTCACGACACGAGCTGACGACAGCCATGCAGCACC

TGTGTTCGAGTTCCCGAAGGCACCAATCCATCTCTGGAAAGTTCTCGACATGTCAAGACC

AGGTAAGGTTCTTCGCGTTGCATCGAATTAAACCACATACTCCACCGCTTGTGCGGGCCC

CCGTCAATTCCTTTGAGTTTCAGTCTTGCGACCGTACTCCCCAGGCGGCGAACTTAACGC

GTTAGCTTCGATACTGCGTGCCAAATTGCACCCAACATCCAGTTCGCATCGTTTAGGGCG

TGGACTACCAGGGTATCTAATCCTGTTTGCTCCCCACGCTTTCGTGCCTCAGTGTCAGTG

TTGGTCCAGGTAGCTGCCTTCGCCATGGATGTTCCTCCCGATCTCTACGCATTTCACTGC

TACACCGGGAATTCCACTACCCTCTACCACACTCTAGTCGCCCAGTATCCACTGCAATTC

CCAGGTTGAGCCCAGGGCTTTCACAACAGACTTAAACAACCACCTACGCACGCTTTACGC

CCAGTAATTCCGAGTAACGCTTGCACCCTTCGTATTACCGCGGCTGCTGGCACGAAGTTA

GCCGGTGCTTATTCTTTGGGTACCGTCAGAACAACCGAGTATTAATCGACTGCTTTTCTT

TCCCAACAAAAGGGCTTTACAACCCGAAGGCCTTCTTCACCCACGCGGTAGGGCTGGATC

AGGCTTGCGCCCATTGTCCAATATTCCCCACTGCTGCCTCCCGTAGGAATCTGGACCGTG

TCTCAGTTCCAGTGTGGCTGATCATCCTCTCAGACCAGCTACGGATCGTCGCCTTGGTGG

GCCTTTACCCCGCCAACTAGCTAATCCGACATCGGCTCATCTATCCGCGCAAGGCCCGAA

GGTCCCCTGCTTTCACCCGAAGGTCGTATGCGGTATTAGCGTAAGTTTCCCTACGTTATC

CCCCACGAAAAGGTAGATTCCGATGTATTCCTCACCCGTCCGCCACTCGCCACCCATAAG

AGCAAGCTCTTACTGTGCTGCCGTTCGACTTGCATGTGTTAGC

>E9_Microbacterium_sp.

CCATTCGAGGCTCCCTCCAAAGGTTAGGCCACCGGCTTCAGGTGTTACCGACTTTCATGA

CTTGACGGGCGGTGTGTACAAGACCCGGGAACGTATTCACCGCAGCGTTGCTGATCTGCG

ATTACTAGCGACTCCGACTTCATGAGGTCGAGTTGCAGACCTCAATCCGAACTGGGACCG

GCTTTTTGGGATTCGCTCCACCTCACGGTATTGCAGCCCTTTGTACCGGCCATTGTAGCA

TGCGTGAAGCCCAAGACATAAGGGGCATGATGATTTGACGTCATCCCCACCTCCCTCCGA

GTTGACCCCGGCAGTATCCCATGAGTTCCCACCATTACGTGCTGGCAACATAGAACGAGG

GTTGCGCTCGTTGCGGGACTTAACCCAACATCTCACGACACGAGCTGACGACAACCATGC

ACCACCTGTTTACGAGTGTCCAAAGAGTTGACCATTTCTGGCCCGTTCTCGTATATGTCA

AGCCTTGGTAAGGTTCTTCGCGTTGCATCGAATTAATCCGCATGCTCCGCCGCTTGTGCG

GGTCCCCGTCAATTCCTTTGAGTTTTAGCCTTGCGGCCGTACTCCCCAGGCGGGGAACTT

AATGCGTTAGCTGCGTCACGGAATCCGTGGAATGGACCCCACAACTAGTTCCCAACGTTT

ACGGGGTGGACTACCAGGGTATCTAAGCCTGTTTGCTCCCCACCCTTTCGCTCCTCAGCG

TCAGTTACGGCCCAGAGATCTGCCTTCGCCATCGGTGTTCCTCCTGATATCTGCGCATTC

CACCGCTACACCAGGAATTCCAATCTCCCCTACCGCACTCTAGTCTGCCCGTACCCACTG

CAGGCCGGAGGTTGAGCCTCCGGATTTCACAGCAGACGCGACAAACCGCCTACGAGCTCT

TTACGCCCAATAATTCCGGATAACGCTTGCGCCCTACGTATTACCGCGGCTGCTGGCACG

TAGTTAGCCGGCGCTTTTTCTGCAGGTACCGTCACTTTCGCTTCTTCCCTGCTAAAAGAG

GTTTACAACCCGAAGGCCGTCATCCCTCACGCGGCGTTGCTGCATCAGGCTTGCGCCCAT

TGTGCAATATTCCCCACTGCTGCCTCCCGTAGGAGTCTGGGCCGTGTCTCAGTCCCAGTG

TGGCCGGTCACCCTCTCAGGCCGGCTACCCGTCGACGCCTTGGTGAGCCATTACCTCACC

AACAAGCTGATAGGCCGCGAGCCCATCCCCAACCGAAATTCTTTCCAGACGCAGACCATG

CGGTCACGTCACATATCCAGTATTAGACGCCGTTTCCAGCGCTTATCCCAGAGTCAGGGG

CAGGTTGCTCACGTGTTACTCACCCGTTCGCCACTGATCCACCCAGCAAGCTGGGCTTCA

CCGTTCGACTTGCAT

>E10_Microbacterium_phyllosphaerae

GTAGCCGGCCTGAGAGGGTGACCGGCCACACTGGGACTGAGACACGGCCCAGACTCCTAC

GGGAGGCAGCAGTGGGGAATATTGCACAATGGGCGCAAGCCTGATGCAGCAACGCCGCGT

GAGGGATGACGGCCTTCGGGTTGTAAACCTCTTTTAGCATGGAAGAAGCGAAAGTGACGG

TACCTGCAGAAAAAGCGCCGGCTAACTACGTGCCAGCAGCCGCGGTAATACGTATGGCGC

AAGCGTTATCCGGAATTATTGGGCGTAAAGAGCTCGTAGGCGGTTTGTCGCGTCTGCTGT

GAAATCCGGAGGCTCAACCTCCGGCCTGCAGTGGGTACGGTCAGACTAGAGTGCGGTAGG

GGAGATTGGAATTCCTGGTGTAGCGGTGGAATGCGCAGATATCAGGAGGAACACCGATGG

CGAAGGCAGATCTCTGGGCCGTAACTGACGCTGAGGAGCGAAAGGGTGGGGAGCAAACAG

GCTTAGATACCCTGGTAGTCCACCCCGTAAACGTTGGGAACTAGTTGTGGGGTCCATTCC

ACGGATTCCGTGACGCAGCTAACGCATTAAGTTCCTCGCCTGGGGAGTACGGCCGCAAGG

CTAA

>E11_Microbacterium_sp.

ATGCAAGTCGAACGGTGAAGCCCAGCTTGCTGGGTGGATCAGTGGCGAACGGGTGAGTAA

CACGTGAGCAACCTGCCCCTGACTCTGGGATAAGCGCTGGAAACGGCGTCTAATACTGGA

TATGTGACGTGACCGCATGGTCTGCGTCTGGAAAGAATTTCGGTTGGGGATGGGCTCGCG

GCCTATCAGCTTGTTGGTGAGGTAATGGCTCACCAAGGCGTCGACGGGTAGCCGGCCTGA

GAGGGTGACCGGCCACACTGGGACTGAGACACGGCCCAGACTCCTACGGGAGGCAGCAGT

GGGGAATATTGCACAATGGGCGCAAGCCTGATGCAGCAACGCCGCGTGAGGGATGACGGC

CTTCGGGTTGTAAACCTCTTTTAGCAGGGAAGAAGCGAAAGTGACGGTACCTGCAGAAAA

AGCGCCGGCTAACTACGTGCCAGCAGCCGCGGTAATACGTAGGGCGCAAGCGTTATCCGG

AATTATTGGGCGTAAAGAGCTCGTAGGCGGTTTGTCGCGTCTGCTGTGAAATCCGGAGGC

TCAACCTCCGGCCTGCAGTGGGTACGGGCAGACTAGAGTGCGGTAGGGGAGATTGGAATT

CCTGGTGTAGCGGTGGAATGCGCAGATATCAGGAGGAACACCGATGGCGAAGGCAGATCT

CTGGGCCGTAACTGACGCTGAGGAGCGAAAGGGTGGGGAGCAAACAGGCTTAGATACCCT

GGTAGTCCACCCCGTAAACGTTGGGAACTAGTTGTGGGGTCCATTCCACGGATTCCGTGA

CGCAGCTAACGCATTAAGTTCCCCGCCTGGGGAGTACGGCCGCAAGGCTAAAACTCAAAG

GAATTGACGGGGACCCGCACAAGCGGCGGAGCATGCGGATTAATTCGATGCAACGCGAAG

AACCTTACCAAGGCTTGACATATACGAGAACGGGCCAGAAATGGTCAACTCTTTGGACAC

TCGTAAACAGGTGGTGCATGGTTGTCGTCAGCTCGTGTCGTGAGATGTTGGGTTAAGTCC

CGCAACGAGCGCAACCCTCGTTCTATGTTGCCAGCACGTAATGGTGGGAACTCATGGGAT

ACTGCCGGGGTCAACTCGGAGGAAGGTGGGGATGACGTCAAATCATCATGCCCCTTATGT

CTTGGGCTTCACGCATGCTACAATGGCCGGTACAAAGGGCTGCAATACCGTGAGGTGGAA

CGAATCCCAAAAAGCCGGTCCCAGTTCGGATTGAGGTCTGCAACTCGACCTCATGAAGTC

GGAGTCGCTAGTAATCGCAGATCAGCAACGCTGCGGTGAATACGTTCCCGGGTCTTGTAC

ACACCGCCCGTCAAGTCATGAAAGTCGGTAAACCTGAAGCCGGTGGCCTAACCTTTGGAG

GGAGCCGTCGAAGG

>E12_Pseudomonas_sp.

GGTACCGTCCTCCCGAAGGTTAGACTAGCTACTTCTGGTGCAACCCACTCCCATGGTGTG

ACGGGCGGTGTGTACAAGGCCCGGGAACGTATTCACCGCGACATTCTGATTCGCGATTAC

TAGCGATTCCGACTTCACGCAGTCGAGTTGCAGACTGCGATCCGGACTACGATCGGTTTT

GTGGGATTAGCTCCACCTCGCGGCTTGGCAACCCTCTGTACCGACCATTGTAGCACGTGT

GTAGCCCAGGCCGTAAGGGCCATGATGACTTGACGTCATCCCCACCTTCCTCCGGTTTGT

CACCGGCAGTCTCCTTAGAGTGCCCACCATGACGTGCTGGTAACTAAGGACAAGGGTTGC

GCTCGTTACGGGACTTAACCCAACATCTCACGACACGAGCTGACGACAGCCATGCAGCAC

CTGTCTCAATGTTCCCGAAGGCACCAATCTATCTCTAGAAAGTTCATTGGATGTCAAGGC

CTGGTAAGGTTCTTCGCGTAGCTTCGAATTAAACCACATGCTCCACCGCTTGTGCGAGCC

CCCGTCAATTCATCTGAGTTCTAACCTTGCGGCCGTACTCACCAGGCGGTCAACTTAATG

CGTTAGCTGCGCCACTAAAAGCTCAAGGCTTCCAACGGCTAGTTGACATCGTTTACGGCG

TGGACTACCAGGGTATCTAATCCTGTATGCTCCCCACGCTTTCGCACCTCAGTGTCAGTA

TTAGTCCAGGTGGTCGCCTTCGCCACTGGTGTTCCTTCCTATATCTACGCATTTCACCGC

TACACAGGAAATTCCACCACCCTCTACCATACTCTAGTCAGTCAGTTTTGAATGCAGTTC

CCAGGTTGAGCCCGGGGATTTCACATCCAACTTAACAAACCACCTACGCGCGCTTTACGC

CCAGTAATTCCGATTAACGCTTGCACCCTCTGTATTACCGCGGCTGCTGGCACAGAGTTA

GCCGGTGCTTATTCTGTCGGTAACGTCAAAACAGCAAAGTATTAATTTACTGCCCTTCCT

CCCAACTTAAAGTGCTTTACAATCCGAAGACCTTCTTCACACACGCGGCATGGCTGGATC

AGGCTTTCGCCCATTGTCCAATATTCCCCAATGCTGCCTCCCGTAGGAGTCTGGACCGTG

TCTCAGTTCCAGTGTGACTGATCATCCTCTCAGACCAGTTACGGATCGTCGCCTTGGTGA

GCCATTACCTCACCAACTAGCTAATCCGACCTAGGCTCATCTGATAGCGCAAGGCCCGAA

GGTCCCCTGCTTTCTCCCGTAGGACGTATGCGGTATTAGCGTCCGTTTCCGAACGTTATC

CCCCACTACCAGGCAGATTCCTAGGCATTACTCCCCCGTCCGCCGCTCTCCAGAGAAGCA

AGCTTCTCTCTACCGCTCGACTTGCAC

>F1_Pseudomonas_sp.

TACCGTCCTCCCGAAGGTTAGACTAGCTACTTCTGGTGCAACCCACTCCCATGGTGTGAC

GGGCGGTGTGTACAAGGCCCGGGAACGTATTCACCGTGACATTCTGATTCACGATTACTA

GCGATTCCGACTTCACGCAGTCGAGTTGCAGACTGCGATCCGGACTACGATCGGTTTTAT

GGGATTAGCTCCACCTCGCGGCTTGGCAACCCTTTGTACCGACCATTGTAGCACGTGTGT

AGCCCAGGCCGTAAGGGCCATGATGACTTGACGTCATCCCCACCTTCCTCCGGTTTGTCA

CCGGCAGTCTCCTTAGAGTGCCCACCATAACGTGCTGGTAACTAAGGACAAGGGTTGCGC

TCGTTACGGGACTTAACCCAACATCTCACGACACGAGCTGACGACAGCCATGCAGCACCT

GTCTCAATGTTCCCGAAGGCACCAATCCATCTCTGGAAAGTTCATTGGATGTCAAGGCCT

GGTAAGGTTCTTCGCGTTGCTTCGAATTAAACCACATGCTCCACCGCTTGTGCGGGCCCC

CGTCAATTCATTTGAGTTTTAACCTTGCGGCCGTACTCCCCAGGCGGTCAACTTAATGCG

TTAGCTGCGCCACTAAGAGCTCAAGGCTCCCAACGGCTAGTTGACATCGTTTACGGCGTG

GACTACCAGGGTATCTAATCCTGTTTGCTCCCCACGCTTTCGCACCTCAGTGTCAGTATC

AGTCCAGGTGGTCGCCTTCGCCACTGGTGTTCCTTCCTATATCTACGCATTTCACCGCTA

CACAGGAAATTCCACCACCCTCTACCATACTCTAGCTCGACAGTTTTGAATGCAGTTCCC

AGGTTGAGCCCGGGGATTTCACATCCAACTTAACGAACCACCTACGCGCGCTTTACGCCC

AGTAATTCCGATTAACGCTTGCACCCTCTGTATTACCGCGGCTGCTGGCACAGAGTTAGC

CGGTGCTTATTCTGTCGGTAACGTCAAAACAGCAACGTATTAAGTTACTGCCCTTCCTCC

CAACTTAAAGTGCTTTACAATCCGAAGACCTTCTTCACACACGCGGCATGGCTGGATCAG

GCTTTCGCCCATTGTCCAATATTCCCCACTGCTGCCTCCCGTAGGAGTCTGGACCGTGTC

TCAGTTCCAGTGTGACTGATCATCCTCTCAGACCAGTTACGGATCGTCGCCTTGGTGAGC

CATTACCTCACCAACTAGCTAATCCGACCTAGGCTCATCTGATAGCGCAAGGCCCGAAGG

TCCCCTGCTTTCTCCCGTAGGACGTATGCGGTATTAGCGTTCCTTTCGAAACGTTGTCCC

CCACTACCAGGCAGATTCCTAGGCATTACTCACCCGTCCGCCGCTGAATCCAGGAGCAAG

CTCCTCTCATCCGCTCGACTTGCATGTGTTAGGC

>F2_Pseudomonas_sp.

TACCGTCCTCCCGAAGGTTAGACTAGCTACTTCTGGTGCAACCCACTCCCATGGTGTGAC

GGGCGGTGTGTACAAGGCCCGGGAACGTATTCACCGTGACATTCTGATTCACGATTACTA

GCGATTCCGACTTCACGCAGTCGAGTTGCAGACTGCGATCCGGACTACGATCGGTTTTAT

GGGATTAGCTCCACCTCGCGGCTTGGCAACCCTTTGTACCGACCATTGTAGCACGTGTGT

AGCCCAGGCCGTAAGGGCCATGATGACTTGACGTCATCCCCACCTTCCTCCGGTTTGTCA

CCGGCAGTCTCCTTAGAGTGCCCACCATAACGTGCTGGTAACTAAGGACAAGGGGTTGCG

CTTCGTTACGGGGACTTAAACCCAAACATCTCACGACACGAGCTGACGACAGCCCATGCA

AGCACCTGTCTCAATGTTCCCGAAGGCACCCAATCCATTCTCTGGAAAGTTCATTGGATG

TCAAGGCCTGGTAAGGTTTCTTCGCGTTGCTTCGAATTAAACCACCATGCTTCCACCGCT

TGTGCGGGCCCCCCGTCAATTTCATTTGAGTTTTAACCTTGCGGCCGTACTCCCCCAGGC

GGTCAACTTAATGCGTTAGCTGCGCCACTAAGAGCTCAAGGCTCCCAACGGCTAGTTGAC

ATCGTTTACGGCGTGGACTACCAGGGTATCTAATCCTGTTTGCTCCCCACGCTTTCGCAC

CTCAGTGTCAGTATCAGTCCAGGTGGTCGCCTTCGCCACTGGTGTTCCTTCCTATATCTA

CGCATTTCACCGCTACACCAGGAAATTCCACCCACCCTCTACCATACTCTAGCTCGACAG

TTTTGAATGCAGTTCCCAGGTTGAGCCCGGGGATTTCACATCCAACTTAACGAACCACCT

ACGCGCGCTTTACGCCCAGTAATTCCGATTAACGCTTGCACCCTCTGTATTACCGCGGCT

GCTGGCACAGAGTTAGCCGGTGCTTATTCTGTCGGTAACGTCAAAACAGCAACGTATTAA

GTTACTGCCCTTCCTCCCAACTTAAAGTGCTTTACAATCCGAAGACCTTCTTCACACACG

CGGCATGGCTGGATCAGGCTTTCGCCCATTGTCCAATATTCCCCACTGCTGCCTCCCGTA

GGAGTCTGGACCGTGTCTCAGTTCCAGTGTGACTGATCATCCTCTCAGACCAGTTACGGA

TCGTCGCCTTGGTGAGCCATTACCTCACCAACTAGCTAATCCGACCTAGGCTCATCTGAT

AGCGCAAGGCCCGAAGGTCCCCTGCTTTCTCCCGTAGGACGTATGCGGTATTAGCGTTCC

TTTCGAAACGTTGTCCCCCACTACCAGGCAGATTCCTAGGCATTACTCACCCGTCCGCCG

CTGAATCCAGGAGCAAGCTCCTCTCATCCGCTCGACTTGCAT

>F3_Streptomyces_sp._forward

KKNTTNNMMATGCAAGTCGAACGATGAAGCCCTTCGGGGTGGATTAGTGGCGAACGGGTGAGTAACACGTGGGCAATCTG

CCCTTCACTCTGGGACAAGCCCTGGAAACGGGGTCTAATACCGGATAACACTCTGTCCCTCATGGGGCGGGGTTAAAAGC

TCCGGCGGTGAAGGATGAGCCCGCGGCCTATCAGCTTGTTGGTGGGGTAATGGCCTACCAAGGCGACGACGGGTAGCCGG

CCTGAGAGGGCGACCGGCCACACTGGGACTGAGACACGGCCCAGACTCCTACGGGAGGCAGCAGTGGGGAATATTGCACA

ATGGGCGAAAGCCTGATGCAGCGACGCCGCGTGAGGGATGACGGCCTTCGGGTTGTAAACCTCTTTCAGCAGGGAAGAAG

CGCAAGTGACGGTACCTGCAGAAGAAGCACCGGCTAACTACGTGCCAGCAGCCGCGGTAATACGTAGGGTGCGAGCGTTG

TCCGGAATTATTGGGCGTAAAGAGCTCGTAGGCGGCTTGTCACGTCGGATGTGAAAGCTCGGGGCTTAACCCCGAGTCTG

CATTCGATACGGGCTAGCTAGAGTGTGGTAGGGGAGATCGGAATTCCTGGTGTAGCGGTGAAATGCGCAGATATCAGGAG

GAACACCGGTGGCGAAGGCGGATCTCTGGGCCATTACTGACGCTGAGGAGCGAAAGCGTGGGGAGCGAACAGGATTAGAT

ACCCTGGTAGTCCACGCCGTAAACGTTGGGAACTAGGTGTTGGCGACATTCCACGTCGTCGGTGCCGCAGCTAACGCATT

AAGTTCCCCGCCTGGGGAGTACGGCCGCAAGGCTAAAACTCAAAGGAATTGACGGGGGCCCGCACAAGCAGCGGAGCATG

TGGCTTAATTCGACGCAACGCGAAGAACCTTACCAAGGCTTGACATATACCGGAAAGCATCAGAGATGGTGCCCCCCTTG

TGGTCGGTATACAGGTGGTGCATGGCTGTCGTCAGCTCGTGTCGTGAGATGTTGGGTTAAGTCCCGCAACGAGCGCAACC

CTTGTNCTGTGTTGCCAGCATGCCCTTCGGGNTGATGGGNACTNMNAGGN

>F4_Pseudomonas_brenneri

ATGCAAGTCGAGCGGTAGAGAGGAGCTTGCTTCTCTTGAGAGCGGCGGACGGGTGAGTAA

TGCCTAGGAATCTGCCTGGTAGTGGGGGATAACGTTCGGAAACGGACGCTAATACCGCAT

ACGTCCTACGGGAGAAAGCAGGGGACCTTCGGGCCTTGCGCTATCAGATGAGCCTAGGTC

GGATTAGCTAGTTGGTGAGGTAATGGCTCACCAAGGCGACGATCCGTAACTGGTCTGAGA

GGATGATCAGTCACACTGGAACTGAGACACGGTCCAGACTCCTACGGGAGGCAGCAGTGG

GGAATATTGGACAATGGGCGAAAGCCTGATCCAGCCATGCCGCGTGTGTGAAGAAGGTCT

TCGGATTGTAAAGCACTTTAAGTTGGGAGGAAGGGCAGTAAATTAATACTTTGCTGTTTT

GACGTTACCGACAGAATAAGCACCGGCTAACTCTGTGCCAGCAGCCGCGGTAATACAGAG

GGTGCAAGCGTTAATCGGAATTACTGGGCGTAAAGCGCGCGTAGGTGGTTTGTTAAGTTG

GATGTGAAATCCCCGGGCTCAACCTGGGAACTGCATTCAAAACTGACTGACTAGAGTATG

GTAGAGGGTGGGTGGAATTTCCTGTGTAGCGGTGAAATGCGTAGATATAGGAAGGAACAC

CAGTGGCGAAGGCGACCACCTGGACTAATACTGACACTGAGGTGCGAAAGCGTGGGGAGC

AAACAGGATTAGATACCCTGGTAGTCCACGCCGTAAACGATGTCAACTAGCCGTTGGAAG

CCTTGAGCTTTTAGTGGCGCAGCTAACGCATTAAGTTGACCGCCTGGGGAGTACGGCCGC

AAGGTTAAAACTCAAATGAATTGACGGGGGCCCGCACAAGCGGTGGAGCATGTGGTTTAA

TTCGAAGCAACGCGAAGAACCTTACCAGGCCTTGACATCCAATGAACTTTCTAGAGATAG

ATTGGTGCCTTCGGGAACATTGAGACAGGTGCTGCATGGCTGTCGTCAGCTCGTGTCGTG

AGATGTTGGGTTAAGTCCCGTAACGAGCGCAACCCTTGTCCTTAGTTACCAGCACGTAAT

GGTGGGCACTCTAAGGAGACTGCCGGTGACAAACCGGAGGAAGGTGGGGATGACGTCAAG

TCATCATGGCCCTTACGGCCTGGGCTACACACGTGCTACAATGGTCGGTACAGAGGGTTG

CCAAGCCGCGAGGTGGAGCTAATCCCACAAAACCGATCGTAGTCCGGATCGCAGTCTGCA

ACTCGACTGCGTGAAGTCGGAATCGCTAGTAATCGCGAATCAGAATGTCGCGGTGAATAC

GTTCCCGGGCCTTGTACACACCGCCCGTCACACCATGGGAGTGGGTTGCACCAGAAGTAG

CTAGTCTAACCTTCGGGAGGACGGTAC

>F5_Stenotrophomonas_rhizophila

ATGCAAGTCGAACGGCAGCACAGTAAGAGCTTGCTCTTATGGGTGGCGAGTGGCGGACGG

GTGAGGAATACATCGGAATCTACCTTTTCGTGGGGGATAACGTAGGGAAACTTACGCTAA

TACCGCATACGACCTTCGGGTGAAAGCAGGGGACCTTCGGGCCTTGCGCGGATAGATGAG

CCGATGTCGGATTAGCTAGTTGGCGGGGTAAAGGCCCACCAAGGCGACGATCCGTAGCTG

GTCTGAGAGGATGATCAGCCACACTGGAACTGAGACACGGTCCAGACTCCTACGGGAGGC

AGCAGTGGGGAATATTGGACAATGGGCGCAAGCCTGATCCAGCCATACCGCGTGGGTGAA

GAAGGCCTTCGGGTTGTAAAGCCCTTTTGTTGGGAAAGAAAAGCAGTCGATTAATACTCG

GTTGTTCTGACGGTACCCAAAGAATAAGCACCGGCTAACTTCGTGCCAGCAGCCGCGGTA

ATACGAAGGGTGCAAGCGTTACTCGGAATTACTGGGCGTAAAGCGTGCGTAGGTGGTTGT

TTAAGTCTGTTGTGAAAGCCCTGGGCTCAACCTGGGAATTGCAGTGGATACTGGGCGACT

AGAGTGTGGTAGAGGGTAGTGGAATTCCCGGTGTAGCAGTGAAATGCGTAGAGATCGGGA

GGAACATCCATGGCGAAGGCAGCTACCTGGACCAACACTGACACTGAGGCACGAAAGCGT

GGGGAGCAAACAGGATTAGATACCCTGGTAGTCCACGCCCTAAACGATGCGAACTGGATG

TTGGGTGCAATTTGGCACGCAGTATCGAAGCTAACGCGTTAAGTTCGCCGCCTGGGGAGT

ACGGTCGCAAGACTGAAACTCAAAGGAATTGACGGGGGCCCGCACAAGCGGTGGAGTATG

TGGTTTAATTCGATGCAACGCGAAGAACCTTACCTGGTCTTGACATGTCGAGAACTTTCC

AGAGATGGATTGGTGCCTTCGGGAACTCGAACACAGGTGCTGCATGGCTGTCGTCAGCTC

GTGTCGTGAGATGTTGGGTTAAGTCCCGCAACGAGCGCAACCCTTGTCCTTAGTTGCCAG

CACGTAATGGTGGGAACTCTAAGGAGACCGCCGGTGACAAACCGGAGAAAGGTGGGGATG

ACGTCAAGTCATCATGGCCCTTACGACCAGGGCTACACACGTACTACAATGGTAGGGACA

GAGGGCTGCAAACCCGCGAGGGCAAGCCAATCCCAGAAACCCTATCTCAGTCCGGATTGG

AGTCTGCAACTCGACTCCATGAAGTCGGAATCGCTAGTAATCGCAGATCAGCATTGCTGC

GGTGAATACGTTCCCGGGCCTTGTACACACCGCCCGTCACACCATGGGAGTTTGTTGCAC

CAGAAGCAGGTAGCTTAACCTTCGGGAGGGCGCTGCCACCG

>F6_Agrobacterium_tumefaciens

CTACGTGGGTTAGCTGCCTCTTGCGGTTAGCGCACTACCTTCGGGTAAACCAACTCCCAT

GGTGTGACGGGCGGTGTGTACAAGGCCCGGGAACGTATTCACCGCAGCATGCTGATCTGC

GATTACTAGCGATTCCAACTTCATGCACTCGAGTTGCAGAGTGCAATCCGAACTGAGATG

GCTTTTGGAGATTAGCTCGACATCGCTGTCTCGCTGCCCACTGTCACCACCATTGTAGCA

CGTGTGTAGCCCAGCCCGTAAGGGCCATGAGGACTTGACGTCATCCCCACCTTCCTCTCG

GCTTATCACCGGCAGTCCCCTTAGAGTGCCCAACTAAATGCTGGCAACTAAGGGCGAGGG

TTGCGCTCGTTGCGGGACTTAACCCAACATCTCACGACACGAGCTGACGACAGCCATGCA

GCACCTGTTCTGGGGCCAGCCTAACTGAAGGACAATGTCTCCACTGCCCAAACCCCGAAT

GTCAAGAGCTGGTAAGGTTCTGCGCGTTGCTTCGAATTAAACCACATGCTCCACCGCTTG

TGCGGGCCCCCGTCAATTCCTTTGAGTTTTAATCTTGCGACCGTACTCCCCAGGCGGAAT

GTTTAATGCGTTAGCTGCGCCACCGAACAGTATACTGCCCGACGGCTAACATTCATCGTT

TACGGCGTGGACTACCAGGGTATCTAATCCTGTTTGCTCCCCACGCTTTCGCACCTCAGC

GTCAGTAATGGACCAGTAAGCCGCCTTCGCCACTGGTGTTCCTCCGAATATCTACGAATT

TCACCTCTACACTCGGAATTCCACTTACCTCTTCCATACTCAAGATACCCAGTATCAAAG

GCAGTTCCAGAGTTGAGCTCTGGGATTTCACCCCTGACTTAAATATCCGCCTACGTGCGC

TTTACGCCCAGTAATTCCGAACAACGCTAGCCCCCTTCGTATTACCGCGGCTGCTGGCAC

GAAGTTAGCCGGGGCTTCTTCTCCGGATACCGTCATTATCTTCTCCGGTGAAAGAGCTTT

ACAACCCTAAGGCCTTCATCACTCACGCGGCAGGGCTGGATCAGGCTTGCGCCCATTGTC

CAATATTCCCCACTGCTGCCTCCCGTAGGAATTTGGGCCGTGTCTCAGTCCCAATGTGGC

TGATCATCCTCTCAGACCAGCTATGGATCGTCGCCTTGGTAGGCCTTTACCCCACCAACT

AGCTAATCCAACGCGGGCTCATCATACCCCGATAAATCTTTCCCCCGTAGGGCGTATGCG

GTATTAATTCCAGTTTCCCGGAGCTATTCCGCAGGGCACGGTAGATTCCCACGCGTTACT

CACCCGTCTGCCACTCCCCATGCGGGCGTTCGACTTGCA

>F7_Stenotrophomonas_sp.

CGTGGGCAAGCGCCCTCCCGAAGGTTAAGCTACCTGCTTCTGGTGCAACAAACTCCCATG

GTGTGACGGGCGGTGTGTACAAGGCCCGGGAACGTATTCACCGCAGCAATGCTGATCTGC

GATTACTAGCGATTCCGACTTCATGGAGTCGAGTTGCAGACTCCAATCCGGACTGAGATA

GGGTTTCTGGGATTGGCTTGCCCTCGCGGGTTTGCAGCCCTCTGTCCCTACCATTGTAGT

ACGTGTGTAGCCCTGGTCGTAAGGGCCATGATGACTTGACGTCATCCCCACCTTCCTCCG

GTTTGTCACCGGCGGTCTCCTTAGAGTTCCCACCATTACGTGCTGGCAACTAAGGACAAG

GGTTGCGCTCGTTGCGGGACTTAACCCAACATCTCACGACACGAGCTGACGACAGCCATG

CAGCACCTGTGTTCGAGTTCCCGAAGGCACCAATCCATCTCTGGAAAGTTCTCGACATGT

CAAGACCAGGTAAGGTTCTTCGCGTTGCATCGAATTAAACCACATACTCCACCGCTTGTG

CGGGCCCCCGTCAATTCCTTTGAGTTTCAGTCTTGCGACCGTACTCCCCAGGCGGCGAAC

TTAACGCGTTAGCTTCGATACTGCGTGCCAAATTGCACCCAACATCCAGTTCGCATCGTT

TAGGGCGTGGACTACCAGGGTATCTAATCCTGTTTGCTCCCCACGCTTTCGTGCCTCAGT

GTCAGTGTTGGTCCAGGTAGCTGCCTTCGCCATGGATGTTCCTCCCGATCTCTACGCATT

TCACTGCTACACCGGGAATTCCACTACCCTCTACCACACTCTAGTCGCCCAGTATCCACT

GCAATTCCCAGGTTGAGCCCAGGGCTTTCACAACAGACTTAAACAACCACCTACGCACGC

TTTACGCCCAGTAATTCCAAGTAACGCTTGCACCCTTCGTATTACCGCGGCTGCTGGCAC

GAAATTAGCCGGTGCTTATTCTTTGGGTACCGTCAGAACAACCGGGTATTAACCGACGGC

TTTTCTTTCCCAACAAAAGGGCTTTACAACCCGAAGGCCTTCTTCACCCACGCGGTAAGG

CTGGATCAGGCTTGCGCCCATTGTCCAATATTCCCCACTGCTGCCTCCCGTAGGAGTCTG

GACCGTGTCTCAGTTCCAGTGTGGCTGATCATCCTCTCAGACCAGCTACGGATCGTCGCC

TTGGTGGGCCTTTACCCCGCCAACTAGCTAATCCGACATCGGCTCATCTATCCGCGCAAG

GCCCGAAGGTCCCCTGCTTTCACCCGAAGGTCGTATGCGGTATTAGCGTAAGTTTCCCTA

CGTTATCCCCCACGAAAAGGTAGATTCCGATGTATTCCTCACCCGTCCGCCACTCGCCAC

CCATAAGAGCAAGCTCTTACTGTGCTGCCGTTCGACTTGCA

>F8_Rhizobium_sp._forward

NNNNTGCANGTCGAACGCCCCGCAAGGGGAGTGGCAGACGGGTGAGTAACACGTGGGAACGTACCCTTTTCTACGGAATA

ACCCAGGGAAACTTGGACTAATACCGTATGTGCCCTTCGGGGGAAAGATTTATCGGAAAAGGATCGGCCCGCGTTGGATT

AGCTAGTTGGTGGGGTAAAGGCCTACCAAGGCGACGATCCATAGCTGGTCTGAGAGGATGATCAGCCACATTGGGACTGA

GACACGGCCCAAACTCCTACGGGAGGCAGCAGTGGGGAATATTGGACAATGGGCGCAAGCCTGATCCAGCCATGCCGCGT

GAGTGATGAAGGCCTTAGGGTTGTAAAGCTCTTTCACCGGAGAAGATAATGACGGTATCCGGAGAAGAAGCCCCGGCTAA

CTTCGTGCCAGCAGCCGCGGTAATACGAAGGGGGCTAGCGTTGTTCGGATTTACTGGGCGTAAAGCGCACGTAGGCGGAT

CGATCAGTCAGGGGTGAAATCCCAGAGCTCAACTCTGGAACTGCCTTTGATACTGTCGATCTGGAGTATGGAAGAGGTGA

GTGGAATTCCGAGTGTAGAGGTGAAATTCGTAGATATTCGGAGGAACACCAGTGGCGAAGGCGGCTCACTGGTCCATTAC

TGACGCTGAGGTGCGAAAGCGTGGGGAGCAAACAGGATTAGATACCCTGGTAGTCCACGCCGTAAACGATGAATGTTAGC

CGTCGGGCAGTATACTGTTCGGTGGCGCAGCTAACGCATTAAACATTCCGCCTGGGGAGTACGGTCGCAAGATTAAAACT

CAAAGGAATTGACGGGGGCCCGCACAAGCGGTGGAGCATGTGGTTTAATTCGAAGCAACGCGCAGAACCTTACCAGCCCT

TGACATGTCCGGCTAKNTACAGAGATGTAGTGTTCCCTTCGGGGACCGGAACACAGGTGCTGCATGGCTGTCGTCAGCTC

GTGTCGTGAGATGTTGGGTTAAGTCCCGCAACGAGCGCAACCCTCGCCCTTAGTTGCCAGCATTTAGTTGGGCACTCTAA

GGGGACTGCCGGTGANNAGCCGAGNGGAAGNNNGGGATGACGTCAAGTCCTCATGGCCCTTACGGNCTGGNCTAMMMNGT

GCTNNNTGG

>F9_Agrobacterium_sp.

GGCTTAACCATGCAAGTCGAACGCCCCGCAGGGGAGTGGCAGACGGGTGAGTAACGCGTG

GGAATCTACCGTGCCCTGCGGAATAGCTCCGGGAAACTGGAATTAATACCGCATACGCCC

TACGGGGGAAAGATTTATCGGGGTATGATGAGCCCGCGTTGGATTAGCTAGTTGGTGGGG

TAAAGGCCTACCAAGGCGACGATCCATAGCTGGTCTGAGAGGATGATCAGCCACATTGGG

AATGAGACACGGCCCAAACTCCTACGGGAGGCAGCAGTGGGGAATATTGGACAATGGGCG

CAAGCCTGATCCAGCCATGCCGCCTGAGTGATGAAGGCCTTAGGGTTGTAAAGCTCTTTC

ACCGGAGAAGATAATGACGGTATCCGGAGAAGAAGCCCCGGCTAAATTCGTGCCAGCAGC

CGCGGTAATACGAAGGGGGCCAGCGTTGTTCGGAATTACTGGGCGTAAAGCGCACGTAGG

CGGATATTTAAGTCAGGGGTGAAATCCCAGAGCTCAACTCCGGAACTGCCTTTGATACTG

GGTATCTTGAGTATGGAAGAGGTAAGTGGAATTCCGAGTGTAGAGGTGAAATTCGTAGAT

ATTCGGAGGAACACCAGTGGCGAAGGCGGCTTACTGGTCCATTACTGACGCTGAGGTGCG

AAAGCGTGGGGAGCAAACAGGATTAGATACCCTGGTAGTCCACGCCGTAAACGATGAATG

TTAGCCGTCGGGCAGTATACTGTTCGGTGGCGCAGCTAACGCATTAAACATTCCGCCTGG

GGAGTACGGTCGCAAGATTAAAACTCAAAGGAATTGACGGGGGCCCGCACAAGCGGTGGA

GCATGTGGTTTAATTCGAAGCAACGCGCAGAACCTTACCAGCTCTTGACATTCGGGGTTT

GGGCAGTGGAGACATTGTCCTTCAGTTAGGCTGGCCCCAGAACAGGTGCTGCATGGCTGT

CGTCAGCTCGTGTCGTGAGATGTTGGGTTAAGTCCCGCAACGAGCGCAACCCTCGCCCTT

AGTTGCCAGCATTTAGTTGGGCACTCTAAGGGGACTGCCGGTGATAACCCAGAGAAAAAG

GTGGGAATGACGTCAAGTCCTCAGGGCCCTTACGGGCTGGGCTACACACGTGCTACAATG

GTGGTGACAGTGGGCAGCGAGACAGCGATGTCGAGCTAATCTCCAAAAGCCATCTCAGTT

CGGATTGCACTCTGCAACTCGAGTGCATGAAGTTGGAATCGCTAGTAATCGCAGATCAGC

ATGCTGCGGTGAATACGTTCCCGGGCCTTGTACACACCGCCCGTCACACCATGGGAGTTG

GTTTACCCGAAGGTAGTGCGCTAACCGCAAGGAGGCAGCTAACCCAC

>F10_Pseudomonas_sp.

GGTAACCGTCCTCCCGAAGGTTAGACTAGCTACTTCTGGTGCAACCCACTCCCATGGTGT

GACGGGCGGTGTGTACAAGGCCCGGGAACGTATTCACCGTGACATTCTGATTCACGATTA

CTAGCGATTCCGACTTCACGCAGTCGAGTTGCAGACTGCGATCCGGACTACGATCGGTTT

TATGGGATTAGCTCCACCTCGCGGCTTGGCAACCCTTTGTACCGACCATTGTAGCACGTG

TGTAGCCCAGGCCGTAAGGGCCATGATGACTTGACGTCATCCCCACCTTCCTCCGGTTTG

TCACCGGCAGTCTCCTTAGAGTGCCCACCATAACGTGCTGGTAACTAAGGACAAGGGTTG

CGCTCGTTACGGGACTTAACCCAACATCTCACGACACGAGCTGACGACGGCCATGCAGCA

CCTGTCTCAATGTTCCCGAAGGCACCAATCCATCTCTGGAAAGTTCATTGGATGTCAAGG

CCTGGTAAGGTTCTTCGCGTTGCTTCGAATTAAACCACATGCTCCACCGCTTGTGCGGGC

CCCCGTCAATTCATTTGAGTTCTAACCTTGCGGCCGTACTCCCCCAGGCGGTCAACTTAA

TGCGTTAGCTGCGCCACTAAGAGCTCAAGGCTCCCAAAGGGCTAGTTGACATCGTGTACG

GCGTGGACTACCAGGGTATCTAATCCTGTTTGCTCCCCACGCTTTCGCACCTCAGTGTCA

GTATCAGTCCAGGTGGTCGCCTTCGCCACTGGTGTTCCTTCCTATATCTACGCATTTCAC

CGCTACACAGGAAATTTCCACCCACCCTCTACCATACTCTAGCTCGACAGTTTTGAATGC

AGTTCCCAGGTTGAGCCCGGGGATTTCACATCCAACTTAACGAACCACCTACGCGCGCTT

TACGCCCAGTAATTCCAATAAACGCTTGCACCCTCTGTATTACCGCGGCTGCTGGCACAG

AGTTAGCCGGTGCTTATTCTGTCGGTAACGTCAAAACAGCAACGTAATAAGTTACTGCCC

TTCCTCCCAACTTAAAGTGCTTTACAATCCGAAGACCTTCTTCACACACGCGGCAGGGCT

GGATCAGGCTTTCGCCCATTGTCCAATATTCCCCACTGCTGCCTCCCGTAGGAATCTGGA

CCGTGTCTCAGTTCCAGTGTGACTGATCATCCTCTCAGACCAGTTACGGATCGTCGCCTT

GGTGAGCCATTACCTCACCAACTAGCTAATCCGACCTAGGCTCATCTGATAGCGCAAGGC

CCGAAGGTCCCCTGCTTTCTCCCGTAGGACGTATGCGGTATTAGCGTTCCTTTCGAAACG

TTGTCCCCCACTACCAGGCAGATTCCTAGGCATTACTCACCCGTCCGCCGCTGAATCCAG

GAGCAAGCTCCTCTC

>F11_Pseudomonas_sp.

GGTAACCGTCCTCCCGAAGGTTAGACTAGCTACTTCTGGTGCAACCCACTCCCATGGTGT

GACGGGCGGTGTGTACAAGGCCCGGGAACGTATTCACCGTGACATTCTGATTCACGATTA

CTAGCGATTCCGACTTCACGCAGTCGAGTTGCAGACTGCGATCCGGACTACGATCGGTTT

TATGGGATTAGCTCCACCTCGCGGCTTGGCAACCCTTTGTACCGACCATTGTAGCACGTG

TGTAGCCCAGGCCGTAAGGGCCATGATGACTTGACGTCATCCCCACCTTCCTCCGGTTTG

TCACCGGCAGTCTCCTTAGAGTGCCCACCATAACGTGCTGGTAACTAAGGACAAGGGTTG

CGCTCGTTACGGGACTTAACCCAACATCTCACGACACGAGCTGACGACAGCCATGCAGCA

CCTGTCTCAATGTTCCCGAAGGCACCAATCCATCTCTGGAAAGTTCATTGGATGTCAAGG

CCTGGTAAGGTTCTTCGCGTTGCTTCGAATTAAACCACATGCTCCACCGCTTGTGCGGGC

CCCCGTCAATTCATTTGAGTTTTAACCTTGCGGCCGTACTCCCCAGGCGGTCAACTTAAT

GCGTTAGCTGCGCCACTAAGAGCTCAAGGCTCCCAACGGCTAGTTGACATCGTTTACGGC

GTGGACTACCAGGGTATCTAATCCTGTTTGCTCCCCACGCTTTCGCACCTCAGTGTCAGT

ATCAGTCCAGGTGGTCGCCTTCGCCACTGGTGTTCCTTCCTATATCTACGCATTTCACCG

CTACACAGGAAATTCCACCACCCTCTACCATACTCTAGCTCGACAGTTTTGAATGCAGTT

CCCAGGTTGAGCCCGGGGATTTCACATCCAACTTAACGAACCACCTACGCGCGCTTTACG

CCCAGTAATTCCGATTAACGCTTGCACCCTCTGTATTACCGCGGCTGCTGGCACAGAGTT

AGCCGGTGCTTATTCTGTCGGTAACGTCAAAACAGCAACGTATTAAGTTACTGCCCTTCC

TCCCAACTTAAAGTGCTTTACAATCCGAAGACCTTCTTCACACACGCGGCATGGCTGGAT

CAGGCTTTCGCCCATTGTCCAATATTCCCCACTGCTGCCTCCCGTAGGAGTCTGGACCGT

GTCTCAGTTCCAGTGTGACTGATCATCCTCTCAGACCAGTTACGGATCGTCGCCTTGGTG

AGCCATTACCTCACCAACTAGCTAATCCGACCTAGGCTCATCTGATAGCGCAAGGCCCGA

AGGTCCCCTGCTTTCTCCCGTAGGACGTATGCGGTATTAGCGTTCCTTTCGAAACGTTGT

CCCCCACTACCAGGCAGATTCCTAGGCATTACTCACCCGTCCGCCGCTGAATCCAGGAGC

AAGCTCCTCTCACCGCTCGACTTGCA

>F12_Stenotrophomonas_sp.

GGCAAGCGCCCTCCCGAAGGTTAAGCTACCTGCTTCTGGTGCAACAAACTCCCATGGTGT

GACGGGCGGTGTGTACAAGGCCCGGGAACGTATTCACCGCAGCAATGCTGATCTGCGATT

ACTAGCGATTCCGACTTCATGGAGTCGAGTTGCAGACTCCAATCCGGACTGAGATAGGGT

TTCTGGGATTGGCTTGCCCTCGCGGGTTTGCAGCCCTCTGTCCCTACCATTGTAGTACGT

GTGTAGCCCTGGTCGTAAGGGCCATGATGACTTGACGTCATCCCCACCTTCCTCCGGTTT

GTCACCGGCGGTCTCCTTAGAGTTCCCACCATTACGTGCTGGCAACTAAGGACAAGGGTT

GCGCTCGTTGCGGGACTTAACCCAACATCTCACGACACGAGCTGACGACAGCCATGCAGC

ACCTGTGTTCGAGTTCCCGAAGGCACCAATCCATCTCTGGAAAGTTCTCGACATGTCAAG

ACCAGGTAAGGTTCTTCGCGTTGCATCGAATTAAACCACATACTCCACCGCTTGTGCGGG

CCCCCGTCAATTCCTTTGAGTTTCAGTCTTGCGACCGTACTCCCCAGGCGGCGAACTTAA

CGCGTTAGCTTCGATACTGCGTGCCAAATTGCACCCAACATCCAGTTCGCATCGTTTAGG

GCGTGGACTACCAGGGTATCTAATCCTGTTTGCTCCCCACGCTTTCGTGCCTCAGTGTCA

GTGTTGGTCCAGGTAGCTGCCTTCGCCATGGATGTTCCTCCCGATCTCTACGCATTTCAC

TGCTACACCGGGAATTCCACTACCCTCTACCACACTCTAGTCGCCCAGTATCCACTGCAA

TTCCCAGGTTGAGCCCAGGGCTTTCACAACAGACTTAAACAACCACCTACGCACGCTTTA

CGCCCAGTAATTCCGAGTAACGCTTGCACCCTTCGTATTACCGCGGCTGCTGGCACGAAG

TTAGCCGGTGCTTATTCTTTGGGTACCGTCAGAACAACCGAGTATTAATCGACTGCTTTT

CTTTCCCAACAAAAGGGCTTTACAACCCGAAGGCCTTCTTCACCCACGCGGTATGGCTGG

ATCAGGCTTGCGCCCATTGTCCAATATTCCCCACTGCTGCCTCCCGTAGGAATCTGGACC

GTGTCTCAGTTCCAGTGTGGCTGATCATCCTCTCAGACCAGCTACGGATCGTCGCCTTGG

TGGGCCTTTACCCCGCCAACTAGCTAATCCGACATCGGCTCATCTATCCGCGCAAGGCCC

GAAGGTCCCCTGCTTTCACCCGAAGGTCGTATGCGGTATTAGCGTAAGTTTCCCTACGTT

ATCCCCCACGAAAAGGTAGATTCCGATGTATTCCTCACCCGTCCGCCACTCGCCACCCAT

AAGAGCAAGCTCTTACTGTGCTGCCGTTCGACTTGCA

>G1_Agrobacterium_sp.

GGTTAGCTGCCTCTTGCGGTTAGCGCACTACCTTCGGGTAAACCAACTCCCATGGTGTGA

CGGGCGGTGTGTACAAGGCCCGGGAACGTATTCACCGCAGCATGCTGATCTGCGATTACT

AGCGATTCCAACTTCATGCACTCGAGTTGCAGAGTGCAATCCGAACTGAGATGGCTTTTG

GAGATTAGCTCGACATCGCTGTCTCGCTGCCCACTGTCACCACCATTGTAGCACGTGTGT

AGCCCAGCCCGTAAGGGCCATGAGGACTTGACGTCATCCCCACCTTCCTCTCGGCTTATC

ACCGGCAGTCCCCTTAGAGTGCCCAACTAAATGCTGGCAACTAAGGGCGAGGGTTGCGCT

CGTTGCGGGACTTAACCCAACATCTCACGACACGAGCTGACGACAGCCATGCAGCACCTG

TTCTGGGGCCAGCCTAACTGAAGGACAATGTCTCCACTGCCCAAACCCCGAATGTCAAGA

GCTGGTAAGGTTCTGCGCGTTGCTTCGAATTAAACCACATGCTCCACCGCTTGTGCGGGC

CCCCGTCAATTCCTTTGAGTTTTAATCTTGCGACCGTACTCCCCAGGCGGAATGTTTAAT

GCGTTAGCTGCGCCACCGAACAGTATACTGCCCGACGGCTAACATTCATCGTTTACGGCG

TGGACTACCAGGGTATCTAATCCTGTTTGCTCCCCACGCTTTCGCACCTCAGCGTCAGTA

ATGGACCAGTAAGCCGCCTTCGCCACTGGTGTTCCTCCGAATATCTACGAATTTCACCTC

TACACTCGGAATTCCACTTACCTCTTCCATACTCAAGATACCCAGTATCAAAGGCAGTTC

CAGAGTTGAGCTCTGGGATTTCACCCCTGACTTAAATATCCGCCTACGTGCGCTTTACGC

CCAGTAATTCCGAACAACGCTAGCCCCCTTCGTATTACCGCGGCTGCTGGCACGAAGTTA

GCCGGGGCTTCTTCTCCGGATACCGTCATTATCTTCTCCGGTGAAAGAGCTTTACAACCC

TAAGGCCTTCATCACTCACGCGGCATGGCTGGATCAGGCTTGCGCCCATTGTCCAATATT

CCCCACTGCTGCCTCCCGTAGGAATTTGGGCCGTGTCTCAGTCCCAATGTGGCTGATCAT

CCTCTCAGAACAGCTATGGATCGTCGCCCTGGTAGGCCTTTACCCCACCAACTAGCTAAT

CCAACGCGGGCTCATCATACCCCGATAAATCTTTCCCCCGTAGGGCGTATGCGGTATTAA

TTCCAGTTTCCCGGAGCTATTCCGCAGGGCACGGTAGATTCCCACGCGTTACTCACCCGT

CTGCCACTCCCCATGCGGGCGTTCGACTTGCATGGTTAAGC

>G2_Stenotrophomonas_rhizophila

GCTACCATGCAAGTCGAACGGCAGCACAGTAAGAGCTTGCTCTTATGGGTGGCGAGTGGC

GGACGGGTGAGGAATACATCGGAATCTACCTTTTCGTGGGGGATAACGTAGGGAAACTTA

CGCTAATACCGCATACGACCTTCGGGTGAAAGCAGGGGACCTTCGGGCCTTGCGCGGATA

GATGAGCCGATGTCGGATTAGCTAGTTGGCGGGGTAAAGGCCCACCAAGGCGACGATCCG

TAGCTGGTCTGAGAGGATGATCAGCCACACTGGAACTGAGACACGGTCCAGACTCCTACG

GGAGGCAGCAGTGGGGAATATTGGACAATGGGCGCAAGCCTGATCCAGCCATACCGCGTG

GGTGAAGAAGGCCTTCGGGTTGTAAAGCCCTTTTGTTGGGAAAGAAAAGCAGTCGATTAA

TACTCGGTTGTTCTGACGGTACCCAAAGAATAAGCACCGGCTAAATTCGTGCCAGCAGCC

GCGGTAATACGAAGGGTGCAAGCGTTACTCGGAATTACTGGGCGTAAAGCGTGCGTAGGT

GGTTGTTTAAGTCTGTTGTGAAAGCCCTGGGCTCAACCTGGGAATTGCAGTGGATACTGG

GCGACTAGAGTGTGGTAGAGGGTAGTGGAATTCCCGGTGTAGCAGTGAAATGCGTAGAGA

TCGGGAGGAACATCCATGGCGAAGGCAGCTACCTGGACCAACACTGACACTGAGGCACGA

AAGCGTGGGGAGCAAACAGGATTAGATACCCTGGTAGTCCACGCCCTAAACGATGCGAAC

TGGATGTTGGGTGCAATTTGGCACGCAGTATCGAAGCTAACGCGTTAAGTTCGCCGCCTG

GGGAGTACGGTCGCAAGACTGAAACTCAAAGGAATTGACGGGGGCCCGCACAAGCGGTGG

AGTATGTGGTTTAATTCGATGCAACGCGAAGAACCTTACCTGGTCTTGACATGTCGAGAA

CTTTCCAGAGATGGATTGGTGCCTTCGGGAACTCGAACACAGGTGCTGCATGGCTGTCGT

CAGCTCGTGTCGTGAGATGTTGGGTTAAGTCCCGCAACGAGCGCAACCCTTGTCCTTAGT

TGCCAGCACGTAATGGTGGGAACTCTAAGGAGACCGCCGGTGACAAACCGGAGGAAGGTG

GGGATGACGTCAAGTCATCATGGCCCTTACGACCAGGGCTACACACGTACTACAATGGTA

GGGACAGAGGGCTGCAAACCCGCGAGGGCAAGCCAATCCCAGAAACCCTATCTCAGTCCG

GATTGGAGTCTGCAACTCGACTCCATGAAGTCGGAATCGCTAGTAATCGCAGATCAGCAT

TGCTGCGGTGAATACGTTCCCGGGCCTTGTACACACCGCCCGTCACACCATGGGAGTTTG

TCACCAGAAGCAGGTAGCTTAACCTTCGGGAGGGCGCTTGCC

>G3_Stenotrophomonas_sp.

TGGCAAGTCGAACGGCAGCACAGTAAGAGCTTGCTCTTATGGGTGGCGAGTGGCGGACGG

GTGAGGAATACATCGGAATCTACCTTTTCGTGGGGGATAACGTAGGGAAACTTACGCTAA

TACCGCATACGACCTTCGGGTGAAAGCAGGGGACCTTCGGGCCTTGCGCGGATAGATGAG

CCGATGTCGGATTAGCTAGTTGGCGGGGTAAAGGCCCACCAAGGCGACGATCCGTAGCTG

GTCTGAGAGGATGATCAGCCACACTGGAACTGAGACACGGTCCAGACTCCTACGGGAGGC

AGCAGTGGGGAATATTGGACAATGGGCGCAAGCCTGATCCAGCCATACCGCGTGGGTGAA

GAAGGCCTTCGGGTTGTAAAGCCCTTTTGTTGGGAAAGAAAAGCAGTCGATTAATACTCG

GTTGTTCTGACGGTACCCAAAGAATAAGCACCGGCTAAATTCGTGCCAGCAGCCGCGGTA

ATACGAAGGGTGCAAGCGTTACTCGGAATTACTGGGCGTAAAGCGTGCGTAGGTGGTTGT

TTAAGTCTGTTGTGAAAGCCCTGGGCTCAACCTGGGAATTGCAGTGGAAACTGGACGACT

AGAGTGTGGTAGAGGGTAGTGGAATTCCCGGTGTAGCAGTGAAATGCGTAGAGATCGGGA

GGAACATCCATGGCGAAGGCAGCTACCTGGACCAACACTGACACTGAGGCACGAAAGCGT

GGGGAGCAAACAGGATTAGATACCCTGGTAGTCCACGCCCTAAACGATGCGAACTGGATG

TTGGGTGCAATTTGGCACGCAGTATCGAAGCTAACGCGTTAAGTTCGCCGCCCGGGGAGT

ACGGTCGCAAGACTGAAACTCAAAGGAATTGACGGGGGCCCGCACAAGCGGTGGAGTATG

TGGTTTAATTCGATGCAACGCGAAGAACCTTACCTGGTCTTGACATGTCGAGAACTTTCC

AGAGATGGATTGGTGCCTTCGGGAACTCGAACACAGGTGCTGCAGGGCTGTCGTCAGCTC

GTGTCGTGAGATGTTGGGTTAAGTCCCGCAACGAGCGCAACCCTTGTCCTTAGTTGCCAG

CACGTAATGGTGGGAACTCTAAGGAGACCGCCGGTGACAAACCGGAGGAAGGTGGGGATG

ACGTCAAGTCATCATGGCCCTTACGACCAGGGCTACACACGTACTACAATGGTAGGGACA

GAGGGCTGCAAACCCGCGAGGGCAAGCCAATCCCAGAAACCCTATCTCAGTCCGGATTGG

AGTCTGCAACTCGACTCCATGAAGTCGGAATCGCTAGTAATCGCAGATCAGCATTGCTGC

GGTGAATACGTTCCCGGGCCTTGTACACACCGCCCGTCACACCATGGGAGTTTGTTGCAC

CAGAAGCAGGTAGCTTAACCTTCGGGAGGGCGCTTGCC

>G4_Agrobacterium_sp.

CTACGTGGGTTAGCTGCCTCTTGCGGTTAGCGCACTACCTTCGGGTAAACCAACTCCCAT

GGTGTGACGGGCGGTGTGTACAAGGCCCGGGAACGTATTCACCGCAGCATGCTGATCTGC

GATTACTAGCGATTCCAACTTCATGCACTCGAGTTGCAGAGTGCAATCCGAACTGAGATG

GCTTTTGGAGATTAGCTCGACATCGCTGTCTCGCTGCCCACTGTCACCACCATTGTAGCA

CGTGTGTAGCCCAGCCCGTAAGGGCCATGAGGACTTGACGTCATCCCCACCCTTCCTCTC

GGCTTATCACCGGCAGTCCCCTTAGAGTGCCCAACTAAATGCTGGCAACTAAGGGCGAGG

GTTGCGCTCGTTGCGGGACTTAACCCAACATCTCACGACACGAGCTGACGACAGCCATGC

AGCACCTGTTCTGGGGCCAGCCTAACTGAAGGACAATGTCTCCACTGCCCAAACCCCGAA

TGTCAAGAGCTGGTAAGGTTCTGCGCGTTGCTTCGAATTAAACCACATGCTCCACCGCTT

GTGCGGGCCCCCGTCAATTCCTTTGAGTTTTAATCTTGCGACCGTACTCCCCAGGCGGAA

TGTTTAATGCGTTAGCTGCGCCACCGAACAGTATACTGCCCGACGGCTAACATTCATCGT

TTACGGCGTGGACTACCAGGGTATCTAATCCTGTTTGCTCCCCACGCTTTCGCACCTCAG

CGTCAGTAATGGACCAGTAAGCCGCCTTCGCCACTGGTGTTCCTCCGAATATCTACGAAT

TTCACCTCTACACTCGGAATTCCACTTACCTCTTCCATACTCAAGATACCCAGTATCAAA

GGCAGTTCCAGAGTTGAGCTCTGGGATTTCACCCCTGACTTAAATATCCGCCTACGTGCG

CTTTACGCCCAGTAATTCCGAACAACGCTAGCCCCCTTCGTATTACCGCGGCTGCTGGCA

CGAAATTAGCCGGGGCTTCTTCTCCGGATACCGTCATTATCTTCTCCGGTGAAAGAGCTT

TACAACCCTAAGGCCTTCATCACTCACGCGGCAGGGCTGGATCAGGCTTGCGCCCATTGT

CCAATATTCCCCACTGCTGCCTCCCGTAAGAATTTGGGCCGGGTCTCAATCCCAATGTGG

CTGAACATCCTCTCAGACCAGCTATGGATCGTCGCCCTGGTAGGCCTTTACCCCACCAAC

TAGCTAATCCAACGCGGGCTCATCATACCCCGATAAATCTTTCCCCCGTAGGGCGTATGC

GGTATTAATTCCAGTTTCCCGGAGCTATTCCGCAGGGCACGGTAGATTCCCACGCGTTAC

TCACCCGTCTGCCACTCCCCTGCGGGCGTTCGACTTGCAT

>G5_Pseudomonas_sp.

CATGCAAGTCGAGCGGTGAAAGGAGCTTGCTCCTGGATTCAGCGGCGGACGGGTGAGTAA

TGCCTAGGAATCTGCCTGGTAGTGGGGGACAACGTTTCGAAAGGAACGCTAATACCGCAT

ACGTCCTACGGGAGAAAGCAGGGGACCTTCGGGCCTTGCGCTATCAGATGAGCCTAGGTC

GGATTAGCTAGTTGGTGAGGTAATGGCTCACCAAGGCGACGATCCGTAACTGGTCTGAGA

GGATGATCAGTCACACTGGAAATGAGACACGGTCCAGACTCCTACGGGAGGCAGCAGTGG

GGAATATTGGACAATGGGCGAAAGCCTGATCCAGCCATGCCGCGTGTGTGAAGAAAGTCT

TCGGATTGTAAAGCACTTTAAGTTGGGAGGAAGGGTTGTAGATTAATACTCTGCAATTTT

GACGTTACCGACAGAATAAGCACCGGCTAACTCTGTGCCAGCAGCCGCGGTAATACAGAG

GGTGCAAGCGTTAATCGGAATTACTGGGCGTAAAGCGCGCGTAGGTGGTTTGTTAAGTTG

GATGTGAAATCCCCGGGCTCAACCTGGGAACTGCATCCAAAACCGGCAAGCTAGAGTATG

GTAGAGGGTGGTGGAATTTCCTGTGTAGCGGTGAAATGCGTAGATATAGGAAGGAACACC

AGTGGCGAACGCGACCACCTGGACTGATACTGACAATGAGGTGCGAAAGCGTGGGGAGCA

AACAGGATTAGATACCCTGGTAGTCCACGCCGTAAACGATGTCAACTAGCCGTTGAGAGC

CTTGAGCTCTTAGTGGCGCACCTAACGCATTAAGTTGACCGCCTGGGGAGTACGGCCGCA

AGGTTAAAACTCAAATGAATTGACGGGGGCCCGCACAAGCGGTGGAGCATGTGGTTTAAT

TCGAAGCAACGCGAAGAACCTTACCAGGCCTTGACATCCAATGAACTTTCCAGAGATGGA

TTGGTGCCTTCGGGAGCATTGAGACAGGTGCTGCATGGCCGTCGTCAGCTCGTGTCGTGA

GATGTTGGGTTAAGTCCCGTAACGAGCGCAACCCTTGTCCTTAGTTACCAGCACGTTATG

GTGGGCACTCTAAGGAGACTGCCGGTGACAAACCGGAGGAAGGTGGGGATGACGTCAAGT

CATCATGGCCCTTACGGCCTGGGCTACACACGTGCTACAATGGTCGGTACAAAGGGTTGC

CAAGCCGCGAGGTGGAGCTAATCCCATAAAACCGATCGTAGTCCGGATCGCAGTCTGCAA

CTCGACTGCGTGAAGTCGGAATCGCTAGTAATCGCGAATCAGAATGTCGCGGTGAATACG

TTCCCGGGCCTGTACACACCGCCCGTCACACCATGGGAGTGGGTTGCACCAGAAGTAGCT

AGTCTAACCTTCGGGAGGACGGTTACC

>G6_Stenotrophomonas_sp.

GGCAGCGCCCTCCCGAAGTAAGCTACCTGCTTCTGGTGCAACAAACTCCCATGGTGTGAC

GGGCGGTGTGTACAGGCCCGGGAACGTATTCACCGCAGCAATGCTGATCTGCGATTACTA

GCGATTCCGACTTCATGGAGTCGAGTTGCAGACTCCAATCCGGACTGAGATAGGGTTTCT

GGGATTGGCTTGCCCTCGCGGGTTTGCAGCCCTCTGTCCCTACCATTGTAGTACGTGTGT

AGCCCTGGTCGTAAGGGCCATGATGACTTGACGTCATCCCCACCTTCCTCCGGTTTGTCA

CCGGCGGTCTCCTTAGAGTTCCCACCATTACGTGCTGGCAACTAAGGACAAGGGTTGCGC

TCGTTGCGGGACTTAACCCAACATCTCACGACACGAGCTGACGACAGCCATGCAGCACCT

GTGTTCGAGTTCCCGAAGGCACCAATCCATCTCTGGAAAGTTCTCGACATGTCAAGACCA

GGTAAGGTTCTTCGCGTTGCATCGAATTAAACCACATACTCCACCGCTTGTGCGGGCCCC

CGTCAATTCCTTTGAGTTTCAGTCTTGCGACCGTACTCCCCAGGCGGCGAACTTAACGCG

TTAGCTTCGATACTGCGTGCCAAATTGCACCCAACATCCAGTTCGCATCGTTTAGGGCGT

GGACTACCAGGGTATCTAATCCTGTTTGCTCCCCACGCTTTCGTGCCTCAGTGTCAGTGT

TGGTCCAGGTAGCTGCCTTCGCCATGGATGTTCCTCCCGATCTCTACGCAATTCACTGCT

ACACCGGGAATTCCACTACCCTCTACCACACTCTAGTCGCCCAGTATCCACTGCAATTCC

CAGGTTGAGCCCAGGGCTTTCACAACAGACTTAAACAACCACCTACGCACGCTTTACGCC

CAGTAATTCCAAGTAACGCTTGCACCCTTCGTATTACCGCGGCTGCTGGCACGAAATTAG

CCGGTGCTTATTCTTTGGGTACCGTCAGAACAACCGGGTATTAACCGACGGCTTTTCTTT

CCCAACAAAAGGGCTTTACAACCCGAAGGCCTTCTTCACCCACCCGGTAGGGCTGGATCA

GGCTTGCGCCCATTGTCCAATATTCCCCACTGCTGCCTCCCGTAAGAATCTGGACCGTGT

CTCAGTTCCAGTGTGGCTGATCATCCTCTCAGACCAGCTACGGATCGTCGCCTTGGTGGG

CCTTTACCCCGCCAACTAGCTAATCCGACATCGGCTCATCTATCCGCGCAAGGCCCGAAG

GTCCCCTGCTTTCACCCGAAGGTCGTATGCGGTATTAGCGTAAGTTTCCCTACGTTATCC

CCCACGAAAAGGTAGATTCCGATGTATTCCTCACCCGTCCGCCACTCGCCACCCATAAGA

GCAAGCTCTTACTGTGCTGCCGTTCGACTTGCATGGTAGC

>G7_Pseudomonas_sp.

GGGTAACCGTCCTCCCGAAGGTTAGACTAGCTACTTCTGGTGCACCCACTCCCATGGTGT

GACGGGCGGTGTGTACAAGGCCCGGGAACGTATTCACCGCGACATTCTGATTCGCGATTA

CTAGCGATTCCGACTTCACGCAGTCGAGTTGCAGACTGCGATCCGGACTACGATCGGTTT

TATGGGATTAGCTCCACCTCGCGGCTTGGCAACCCTTTGTACCGACCATTGTAGCACGTG

TGTAGCCCAGGCCGTAAGGGCCATGATGACTTGACGTCATCCCCACCTTCCTCCGGTTTG

TCACCGGCAGTCTCCTTAGAGTGCCCACCATAACGTGCTGGTAACTAAGGACAAGGGTTG

CGCTCGTTACGGGACTTAACCCAACATCTCACGACACGAGCTGACGACAGCCATGCAGCA

CCTGTCTCAATGCTCCCGAAGGCACCAATCCATCTCCGGAAAGTTCATTGGATGTCAAGG

CCTGGTAAGGTTCTTCGCGTTGCTTCGAATTAAACCACATGCTCCACCGCTTGTGCGGGC

CCCCGTCAATTCATGTGAGTTTTAACCTTGCGGCCGTACTCCCCAGGCGGTCAACTTAAT

GCGTTAGCTGCGCCACTAAGAGCTCAAGGCTCCCAACGGCTAGTTGACATCGTTTACGGC

GTGGACTACCAGGGTATCTAATCCTGTTTGCTCCCCACGCTTTCGCACCTCAGTGTCAGT

ATCAGTCCAGGTGGTCGCCTTCGCCACTGGTGTTCCTTCCTATATCTACGCATTTCACCG

CTACACAGGAAATTCCACCACCCTCTACCATACTCTAGCTTGCCAGTTTTGGATGCAGTT

CCCAGGTTGAGCCCGGGGATTTCACATCCAACTTAACAAACCACCTACGCGCGCTTTACG

CCCAGTAATTCCAATTAACGCTTGCACCCTCTGTATTACCGCGGCTGCTGGCACAGAGTT

AGCCGGTGCTTATTCTGTCGGTAACGTCAAAATTGCAGAGTATTAATCTACAACCCTTCC

TCCCAACTTAAAGTGCTTTACAATCCGAAGACCTTCTTCACACACGCGGCATGGCTGGAT

CAGGCTTTCGCCCATTGTCCAATATTCCCCACTGCTGCCTCCCGTAAGAATCTGGACCGT

GTCTCAATTCCAGTGTGACTGATCATCCTCTCAGACCAGTTACGGATCGTCGCCTTGGTG

AGCCATTACCTCACCAACTAGCTAATCCGACCTAGGCTCATCTGATAGCGCAAGGCCCGA

AGGTCCCCTGCTTTCTCCCGTAGGACGTATGCGGTATTAGCGTTCCTTTCGAAACGTTGT

CCCCCACTACCAGGCAGATTCCTAGGCATTACTCACCCGTCCGCCGCTGAATCCAGGAGC

AAGCTCCTTTCACCGCTCGACTTGCAT

>G8_Achromobacter_sp.

TTAGGCTAACTACTTCTGGTAAACCCACTCCCATGGTGTGACGGGCGGTGTGTACAAGAC

CCGGGAACGTATTCACCGCGACATGCTGATCCGCGATTACTAGCGATTCCGACTTCACGC

AGTCGAGTTGCAGACTGCGATCCGGACTACGATCGGGTTTCTGGGATTGGCTCCCCCTCG

CGGGTTGGCGACCCTCTGTCCCGACCATTGTATGACGTGTGAAGCCCTACCCATAAGGGC

CATGAGGACTTGACGTCATCCCCACCTTCCTCCGGTTTGTCACCGGCAGTCTCATTAGAG

TGCCCTTTCGTAGCACCTAATGACAAGGGTTGCGCTCGTTGCGGGACTTAACCCAACATC

TCACGACACGAGCTGACGACAGCCATGCAGCACCTGTGTTCCGGTTCTCTTGCGAGCACT

TCCAAATCTCTTCGGAATTCCAGACATGTCAAGGGTAGGTAAGGTTTTTCGCGTTGCATC

GAATTAATCCACATCATCCACCGCTTGTGCGGGTCCCCGTCAATTCCTCTGAGTTTTAAT

CTTGCGACCGTACTCCCCAGGCGGTCAACTTCACGCGTAAGCTGCGCTACCAAGGCCCGA

AGGCCCCAACAGCTAGTTGACATCGTTTAGGGCGTGGACTACCAGGGTATCTAATCCTGT

TTGCTCCCCACGCTCTCGTGCATGAGAGTCAGTGTTATCCCAGGAGGCTGCCTTCGCCAT

CGGTGTTCCTCCGCATATCTACGCATTTCACTGCTACACGCGGAATTCCACCTCCCTCTG

ACACACTCTAGCTCGGTAGTTAAAAATGCAGTTCCAAAGTTAAGCTCTGGGATTTCACAT

CTATCTTTCCGAACCGCCGGCGCACGCTTTACGCCCAGTAATTCCAATTAACGCTGGCAC

CCTACGTATTACCGCGGCTGCTGGCACGTAATTAGCCGGTGCTTATTCTGCAGGTACCGT

CAGTTTCACGGGGTATTAGCCCATGACGTTTCTTTCCTGCCAAAAGTGCTTTACAACCCG

AAGGCCTTCATCGCACACGCGGAATGGCTGGATCAGGGTTTCCCCCATTGTCCAAAATTC

CCCACTGCTGCCCCCCGTAAGAATCTGGGCCGTGTCTCAATCCCAGTGTGGCTGGTCGTC

CTCTCAAACCAGCTACGGATCGTCGCCTTGGTGAGCCGTTACCCCACCAACTAGCTAATC

CGATATCGGCCGCTCTAATAGTGCAAGGTCTTGCGATCCCCTGCTTTCCCCCGTAGGGCG

TATGCGGTATAAGCTACGCTTTCGCGTAGTTATCCCCCGCTACTAGGCACGTTCCGATAC

ATTACTCACCCGTTCGCCACTCGCCACCAGACCGAAGTCCGTGCGCCGTTCGACTTGCA

>G9_Pseudomonas_brassicacearum

GGGTAACCGTCCTCCCGAGGTTAGACTAGCTACTTCTGGTGCAACCCACTCCCATGGTGT

GACGGGCGGTGTGTACAAGGCCCGGGAACGTATTCACCGCGACATTCTGATTCGCGATTA

CTAGCGATTCCGACTTCACGCAGTCGAGTTGCAGACTGCGATCCGGACTACGATCGGTTT

TGTGGGATTAGCTCCACCTCGCGGCTTGGCAACCCTCTGTACCGACCATTGTAGCACGTG

TGTAGCCCAGGCCGTAAGGGCCATGATGACTTGACGTCATCCCCACCTTCCTCCGGTTTG

TCACCGGCAGTCTCCTTAGAGTGCCCACCATAACGTGCTGGTAACTAAGGACAAGGGTTG

CGCTCGTTACGGGACTTAACCCAACATCTCACGACACGAGCTGACGACAGCCATGCAGCA

CCTGTCTCAATGTTCCCGAAGGCACCAATCCATCTCTGGAAAGTTCATTGGATGTCAAGG

CCTGGTAAGGTTTCTTCGCGTTGCTTCGAATTAAACCACATGCTCCACCGCTTGTGCGGG

CCCCCGTCAATTCATTTGAGTTTTAACCTTGCGGCCGTACTCCCCAGGCGGTCAACTTAA

TGCGTTAGCTGCGCCACTAAGAGCTCAAGGCTCCCAACGGCTAGTTGACATCGTTTACGG

CGTGGACTACCAGGGTATCTAATCCTGTTTGCTCCCCACGCTTTCGCACCTCAGTGTCAG

TATCAGTCCAGGTGGTCGCCTTCGCCACTGGTGTTCCTTCCTATATCTACGCATTTCACC

GCTACACAGGAAATTCCACCCACCCTCTACCATACTCTAGCTCGACAGTTTTGAATGCAG

TTCCCAGGTTGAGCCCGGGGATTTCACATCCAACTTAACGAACCACCTACGCGCGCTTTA

CGCCCAGTAATTCCGAATTAACGCTTGCACCCTCTGTATTACCGCGGCTGCTGGCACAGA

GTTAGCCGGTGCTTATTCTGTCGGTAACGTCAAAACACTAACGTAATAAGTTAATGCCCT

TCCTCCCAACTTAAAGTGCTTTACAATCCGAAGACCTTCTTCACACACGCGGCAGGGCTG

GATCAGGCTTTCGCCCATTGTCCAATATTCCCCACTGCTGCCTCCCGTAAGAATCTGGAC

CGTGTCTCAATTCCAGTGTGACTGATCATCCTCTCAAACCAGTTACGGATCGTCGCCTTG

GTGAGCCATTACCCCACCAACTAGCTAATCCGACCTAGGCTCATCTGATAGCGCAAGGCC

CGAAGGTCCCCTGCTTTCTCCCGTAGGACGTATGCGGTATTAGCGTCCGTTTCCGAGCGT

TATCCCCCACTACCAGGCAGATTCCTAGGCATTACTCACCCGTCCGCCGCTCTCAAGAGG

TGCAAGCACCTCTCTACCGCTCGACTTGCA

>G10_Pseudomonas_sp.

AGAGCGGCGGACGGGTGAGTAATGCCTAGGAATCTGCCTGGTAGTGGGGGATAACGCTCG

GAAACGGACGCTAATACCGCATACGTCCTACGGGAGAAAGCAGGGGACCTTCGGGCCTTG

CGCTATCAGATGAGCCTAGGTCGGATTAGCTAGTTGGTGAGGTAATGGCTCACCAAGGCG

ACGATCCGTAACTGGTCTGAGAGGATGATCAGTCACACTGGAACTGAGACACGGTCCAGA

CTCCTACGGGAGGCAGCAGTGGGGAATATTGGACAATGGGCGAAAGCCTGATCCAGCCAT

GCCGCGTGTGTGAAGAAAGTCTTCGGATTGTAAAGCACTTTAAGTTGGGAGGAAGGGCAT

TAACCTAATACGTTAGTGTTTTGACGTTACCGACAGAATAAGCACCGGCTAACTCTGTGC

CAGCAGCCGCGGTAATACAGAGGGTGCAAGCGTTAATCGGAATTACTGGGCGTAAAGCGC

GCGTAGGTGGTTCGTTAAGTTGGATGTGAAATCCCCGGGCTCAACCTGGGAACTGCATTC

AAAACCGTCGAGCTAGAGTATGGTAGAGGGTGGGTGGAATTTTCCTGTGGAGCGGTGAAA

TGCGTAGATATAGGAAGGAACACCAGTGGCGAAGGCGACCACCTGGACTGATACTGACAA

TGAGGTGCGAAAGCGTGGGGAGCAAACAGGATTAGATACCCTGGTAGTCCACGCCGTAAA

CGATGTCAACTAGCCCTTGGGAGCCTTGAGCTCTTAGTGGCGCAGCTAACGCATTAAGTT

GACCGCCCGGGGGAGTACGGCCGCAAGGTTAAAACTCAAATGAATTGACGGGGGGCCCGC

ACAAGCGGTGGAACATGTGGTTTAATTTCGAAGCAACGCGAAGAAACCTTACCAGGGCCT

TGACATCCAATGAACTTTCCAGAGATGGATTGGTGCCTTCGGGAACATTGAGACAGGTGC

TGCATGGCTGTCGTCAGCTCGTGTCGTGAGATGTTGGGTTAAGTCCCGTAACGAGCGCAA

CCCTTGTCCTTAGTTACCAGCACGTTATGGTGGGCACTCTAAGGAGACTGCCGGTGACAA

ACCGGAGGAAGGTGGGGATGACGTCAAGTCATCATGGCCCTTACGGCCTGGGCTACACAC

GTGCTACAATGGTCGGTACAGAGGGTTGCCAAGCCGCGAGGTGGAGCTAATCCCACAAAA

CCGATCGTAGTCCGGATCGCAGTCTGCAACTCGACTGCGTGAAGTCGGAATCGCTAGTAA

TCGCGAATCAGAATGTCGCGGTGAATACGTTCCCGGGCCTGTACACACCGCCCGTCACAC

CATGGGAGTGGGTTGCACCAGAAGTAGCTAGTCTAACCTTCGGGGGGACGGTTACC

>G11_Pseudomonas_sp.

TGCAAGGTCGAGCGGAGAGAGGTGCTTGCACCTCTTGAGAGCGGCGGAAGGGTGAGTAAT

GCCTAGGAATCTGCCTGGTAGTGGGGGATAACGCTCGGAAACGGACGCTAATACCGCATA

CGTCCTACGGGAGAAAGCAGGGGACCTTCGGGCCTTGCGCTATCAGATGAGCCTAGGTCG

GATTAGCTAGTTGGTGAGGTAATGGCTCACCAAGGCGACGATCCGTAACTGGTCTGAGAG

GATGATCAGTCACACTGGAACTGAGACACGGTCCAGACTCCTACGGGAGGCAGCAGTGGG

GAATATTGGACAATGGGCGAAAGCCTGATCCAGCCATGCCGCGTGTGTGAAGAAGGTCTT

CGGATTGTAAAGCACTTTAAGTTGGGAGGAAGGGCATTAACCTAATACGTTAGTGTTTTG

ACGTTACCGACAGAATAAGCACCGGCTAACTCTGTGCCAGCAGCCGCGGTAATACAGAGG

GTGCAAGCGTTAATCGGAATTACTGGGCGTAAAGCGCGCGTAGGTGGTTCGTTAAGTTGG

ATGTGAAATCCCCGGGCTCAACCTGGGAACTGCATTCAAAACTGTCGAGCTAGAGTATGG

TAGAGGGTGGGTGGAATTTCCTGTGTAGCGGTGAAATGCGTAGATATAGGAAGGAACACC

AGTGGCGAAGGCGACCACCTGGACTGATACTGACAATGAGGTGCGAAAGCGTGGGGAGCA

AACAGGATTAGATACCCTGGTAGTCCACGCCGTAAACGATGTCAACTAGCCCTTGGGAGC

CTTGAGCTCTTAGTGGCGCAGCTAACGCATTAAGTTGACCGCCTGGGGAGTACGGCCGCA

AGGTTAAAACTCAAATGAATTGACGGGGGCCCGCACAAGCGGTGGAGCATGTGGTTTAAT

TCGAAGCAACGCGAAAAACCTTACCAGGCCTTGACATCCAATGAACTTTCCAGAGATGGA

TTGGTGCCTTCGGGAACATTGAGACAGGTGCTGCATGGCTGTCGTCAGCTCGTGTCGTGA

GATGTTGGGTTAAGTCCCGTAACGAGCGCAACCCTTGTCCTTAGTTACCAGCACGTTATG

GTGGGCACTCTAAGGAGACTGCCGGTGACAAACCGGAGGAAGGTGGGGATGACGTCAAGT

CATCATGGCCCTTACGGCCTGGGCTACACACGTGCTACAATGGTCGGTACAGAGGGTTGC

CAAGCCGCGAGGTGGAGCTAATCCCACAAAACCGATCGTAGTCCGGATCGCAGTCTGCAA

CTCGACTGCGTGAAGTCGGAATCGCTAGTAATCGCGAATCAGAATGTCGCGGTGAATACG

TTCCCGGGCCTTGTACACACCGCCCGTCACACCATGGGAGTGGGTCACCAGAAGTAGCTA

GTCTAACCTTCGGGGGACGGTTACC

>G12_Stenotrophomonas_rhizophila

ATGCAAGTCGAACGGCAGCACAGTAAGAGCTTGCTCTTATGGGTGGCGAGTGGCGGACGG

GTGAGGAATACATCGGAATCTACCTTTTCGTGGGGGATAACGTAGGGAAACTTACGCTAA

TACCGCATACGACCTTCGGGTGAAAGCAGGGGACCTTCGGGCCTTGCGCGGATAGATGAG

CCGATGTCGGATTAGCTAGTTGGCGGGGTAAAGGCCCACCAAGGCGACGATCCGTAGCTG

GTCTGAGAGGATGATCAGCCACACTGGAACTGAGACACGGTCCAGACTCCTACGGGAGGC

AGCAGTGGGGAATATTGGACAATGGGCGCAAGCCTGATCCAGCCATACCGCGTGGGTGAA

GAAGGCCTTCGGGTTGTAAAGCCCTTTTGTTGGGAAAGAAAAGCAGTCGATTAATACTCG

GTTGTTCTGACGGTACCCAAAGAATAAGCACCGGCTAAATTCGTGCCAGCAGCCGCGGTA

ATACGAAGGGTGCAAGCGTTACTCGGAATTACTGGGCGTAAAGCGTGCGTAGGTGGTTGT

TTAAGTCTGTTGTGAAAGCCCTGGGCTCAACCTGGGAATTGCAGTGGATACTGGGCGACT

AGAGTGTGGTAGAGGGTAGTGGAATTCCCGGTGTAGCAGTGAAATGCGTAGAGATCGGGA

GGAACATCCATGGCGAAGGCAGCTACCTGGACCAACACTGACACTGAGGCACGAAAGCGT

GGGGAGCAAACAGGATTAGATACCCTGGTAGTCCACGCCCTAAACGATGCGAACTGGATG

TTGGGTGCAATTTGGCACGCAGTATCGAAGCTAACGCGTTAAGTTCGCCGCCTGGGGAGT

ACGGTCGCAAGACTGAAACTCAAAGGAATTGACGGGGGCCCGCACAAGCGGTGGAGTATG

TGGTTTAATTCGATGCAACGCGAAGAACCTTACCTGGTCTTGACATGTCGAGAACTTTCC

AGAGATGGATTGGTGCCTTCGGGAACTCGAACACAGGTGCTGCATGACTGTCGTCAGCTC

GTGTCCTGAGATGTTGGGTTAAGTCCCGCAACGAGCGCAACCCTTGTCCTTAGTTGCCAG

CACGTAATGGTGGGAACTCTAAGGAGACCGCCGGTGACAAACCGGAGGAAGGTGGGGATG

ACGTCAAGTCATCATGGCCCTTACGACCAGGGCTACACACGTACTACAATGGTAGGGACA

GAGGGCTGCAAACCCGCGAGGGCAAGCCAATCCCAGAAACCCTATCTCAGTCCGGATTGG

AGTCTGCAACTCGACTCCATGAAGTCGGAATCGCTAGTAATCGCAGATCAGCATTGCTGC

GGTGAATACGTTCCCGGGCCTTGTACACACCGCCCGTCACACCATGGGAGTTTGTCACCA

GAAGCAGGTAGCTTAACCTTCGGGAGGGCGCTTG
