# Supplementary material for: Successive cultivation under drought selects for specific microbiome members in the wheat rhizosphere
Source: FEMS Microbiol Ecol. 2026 Apr 14;102(5):fiag037. doi: 10.1093/femsec/fiag037 (PMC13114863; doi:10.1093/femsec/fiag037)
Supplement: fiag037_Supplemental_Files [file fiag037_supplemental_files.zip › Pioppi_Supplementary_v1.pdf]

Table S1. Composition of the bacterial strain library inoculated at the initiation of successive cultivation. The noted taxonomic assignment is based on sanger sequencing of the 16S rRNA gene with both forward and reverse primers. The primer sequences are included in Methods. “*” indicates that the assignment was based only on either the forward or reverse amplified fragment.

Strain no.	Taxonomic assignment	Strain no.	Taxonomic assignment
A1	*Stenotrophomonas maltophilia*	D8	*Stenotrophomonas rhizophila*
A2	*Streptomyces sp.*	D9	*Rahnella aquatilis*
A3	*Variovorax boronicumulans*	D10	*Stenotrophomonas rhizophila*
A4	*Streptomyces sp.*	D11	*Pseudomonas sp.*
A5	*Streptomyces sp.*	D12	*Pseudomonas sp.*
A6	*Agrobacterium tumefaciens*	E1	*Pseudomonas fluorescens*
A7	*Stenotrophomonas sp.*	E2	*Stenotrophomonas rhizophila*
A8	*Ensifer sp.*	E3	*Niallia circulans*
A9	*Agrobacterium tumefaciens*	E4	*Pseudomonas sp.*
A10	*Bacillus sp.*	E5	*Pseudomonas sp.*
A11	*Pseudomonas brassicacearum*	E6	*Pseudomonas poae*
A12	*Stenotrophomonas sp.*	E7	*Rhodococcus qingshengii*
B1	*Variovorax sp. **	E8	*Stenotrophomonas sp.*
B2	*Pseudomonas sp.*	E9	*Microbacterium sp.*
B3	*Pseudomonas sp.*	E10	*Microbacterium phyllosphaerae*
B4	*Brevundimonas sp.*	E11	*Microbacterium sp.*
B5	*Streptomyces sp.*	E12	*Pseudomonas sp.*
B6	*Agrobacterium tumefaciens*	F1	*Pseudomonas sp.*
B7	*Streptomyces sp.*	F2	*Pseudomonas sp.*
B8	*Variovorax boronicumulans*	F3	*Streptomyces sp. **
B9	*Microbacterium sp. **	F4	*Pseudomonas brenneri*
B10	*Pseudomonas fluorescens*	F5	*Stenotrophomonas rhizophila*
B11	*Variovorax boronicumulans*	F6	*Agrobacterium tumefaciens*
B12	*Bacillus sp.* or *Peribacillus sp.*	F7	*Stenotrophomonas sp.*
C1	*Pseudomonas sp.*	F8	*Rhizobium sp. **
C2	*Pseudomonas brassicacearum*	F9	*Agrobacterium sp.*
C3	*Stenotrophomonas sp.*	F10	*Pseudomonas sp.*
C4	*Variovorax paradoxus*	F11	*Pseudomonas sp.*
C5	*Variovorax sp.*	F12	*Stenotrophomonas sp.*
C6	*Microbacterium sp.*	G1	*Agrobacterium sp.*
C7	*Pseudomonas putida*	G2	*Stenotrophomonas rhizophila*
C8	*Stenotrophomonas sp.*	G3	*Stenotrophomonas sp.*
C9	*Stenotrophomonas rhizophila*	G4	*Agrobacterium sp.*
C10	*Pseudomonas sp.*	G5	*Pseudomonas sp.*
C11	*Plantibacter sp.*	G6	*Stenotrophomonas sp.*
C12	*Stenotrophomonas rhizophila*	G7	*Pseudomonas sp.*
D1	*Pseudomonas sp.*	G8	*Achromobacter sp.*
D2	*Stenotrophomonas rhizophila*	G9	*Pseudomonas brassicacearum*
D3	*Stenotrophomonas sp.*	G10	*Pseudomonas sp.*
D4	*Microbacterium sp. **	G11	*Pseudomonas sp.*
D5	*Streptomyces sp*	G12	*Stenotrophomonas rhizophila*
D6	*Bacillus sp. **	V2-1	*Bacillus subtilis*
D7	*Rhodococcus sp.*	V2-4	*Bacillus subtilis*

Table S2. Results for pairwise adonis comparing each combination of treatments. * indicates significant differences (p-value=0.001, p-adjusted = 0.015).

Comparison	Df	SumsOfSqs	F.Model	R2	p.value	p.adjusted	sig
uninoculated_resilient vs library_nostress	1	1.916057	12.05325	0.316747	0.001	0.015	*
uninoculated_resilient vs uninoculated_nostress	1	1.264123	7.37286	0.208433	0.001	0.015	*
uninoculated_resilient vs library_resilient	1	1.440572	7.816309	0.218233	0.001	0.015	*
uninoculated_resilient vs library_susceptible	1	1.586325	7.804712	0.21798	0.001	0.015	*
uninoculated_resilient vs uninoculated_susceptible	1	1.000765	4.433318	0.13669	0.001	0.015	*
library_nostress vs uninoculated_nostress	1	0.92671	8.011352	0.235549	0.001	0.015	*
library_nostress vs library_resilient	1	0.796253	6.148207	0.191246	0.001	0.015	*
library_nostress vs library_susceptible	1	1.375052	9.172123	0.260778	0.001	0.015	*
library_nostress vs uninoculated_susceptible	1	3.169749	18.20325	0.411808	0.001	0.015	*
uninoculated_nostress vs library_resilient	1	1.533839	10.64397	0.275437	0.001	0.015	*
uninoculated_nostress vs library_susceptible	1	1.933876	11.86042	0.297549	0.001	0.015	*
uninoculated_nostress vs uninoculated_susceptible	1	2.903914	15.65133	0.358553	0.001	0.015	*
library_resilient vs library_susceptible	1	0.421312	2.395179	0.078801	0.011	0.165	
library_resilient vs uninoculated_susceptible	1	1.928653	9.721768	0.257723	0.001	0.015	*
library_susceptible vs uninoculated_susceptible	1	1.887102	8.682963	0.236703	0.001	0.015	*

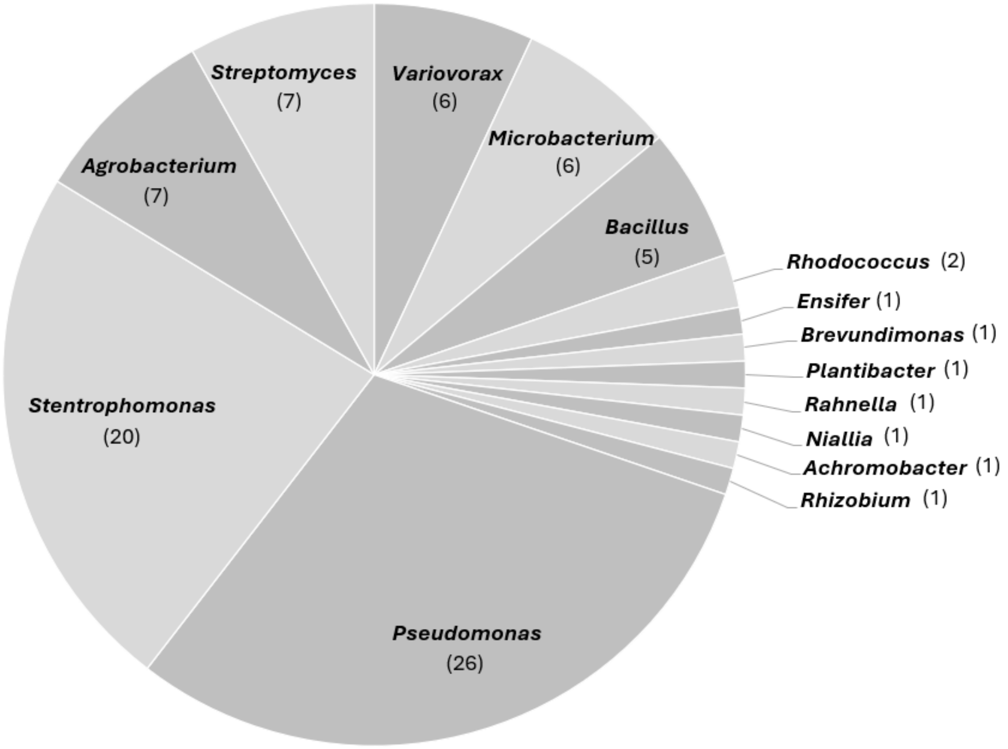

Figure S1. Distribution of bacterial genera included in the library, with the number of strains belonging to each genus noted in parentheses.

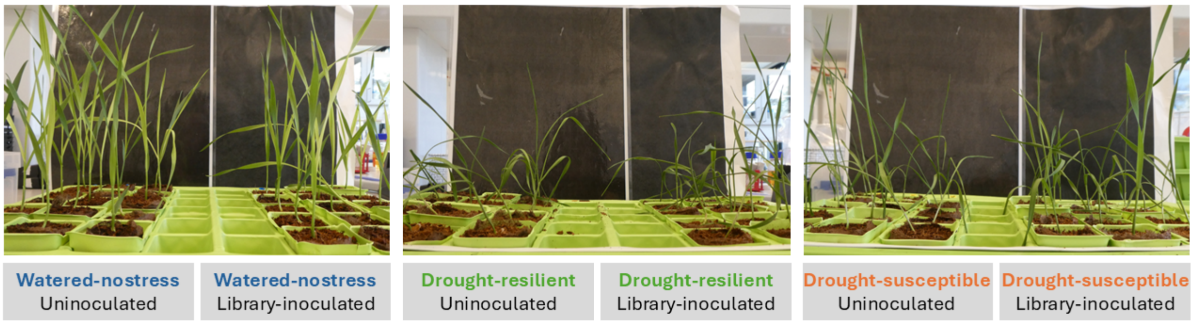

Figure S2. Photos of plants taken on the last day of the 4th cycle, before starting rhizosphere extraction. Selection types and treatments are noted below, distinguishing Uninoculated and Library-inoculated lineages of each selection type.

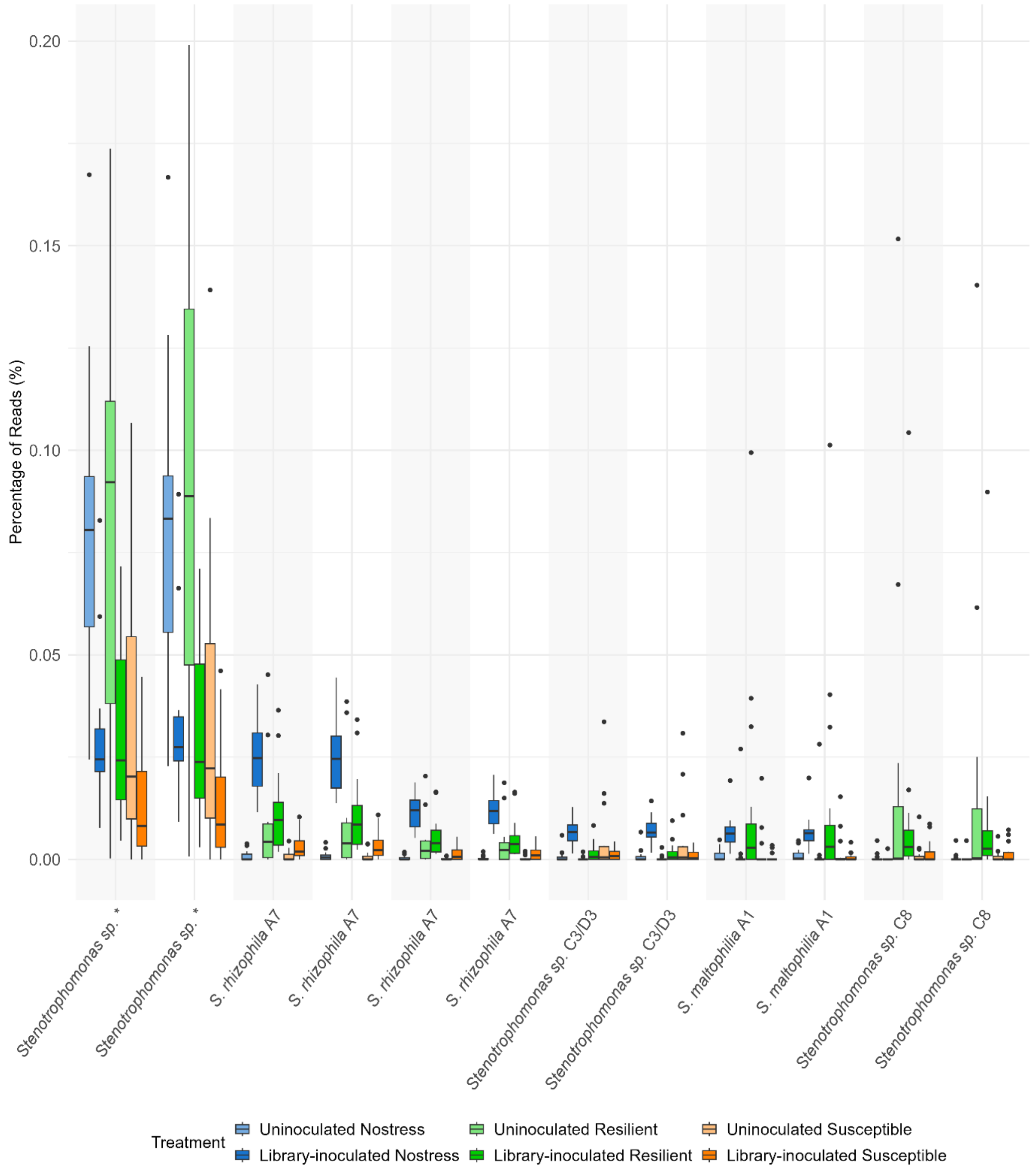

Figure S3. Relative abundance (as percentage of reads) of 12 ASVs assigned to *Stenotrophomonas* within each treatment. Labels indicate which *Stenotrophomonas* library strain each ASV could be matched to, with 100% coverage of the V3-V4 region of the 16S gene. The asterisk (*) indicates an exact match of two ASVs to multiple library strains, specifically C12, D2, D8, D10, E2, F5, F12. The ASVs and corresponding matched library strains are ordered according to similarity in their distribution across treatments.

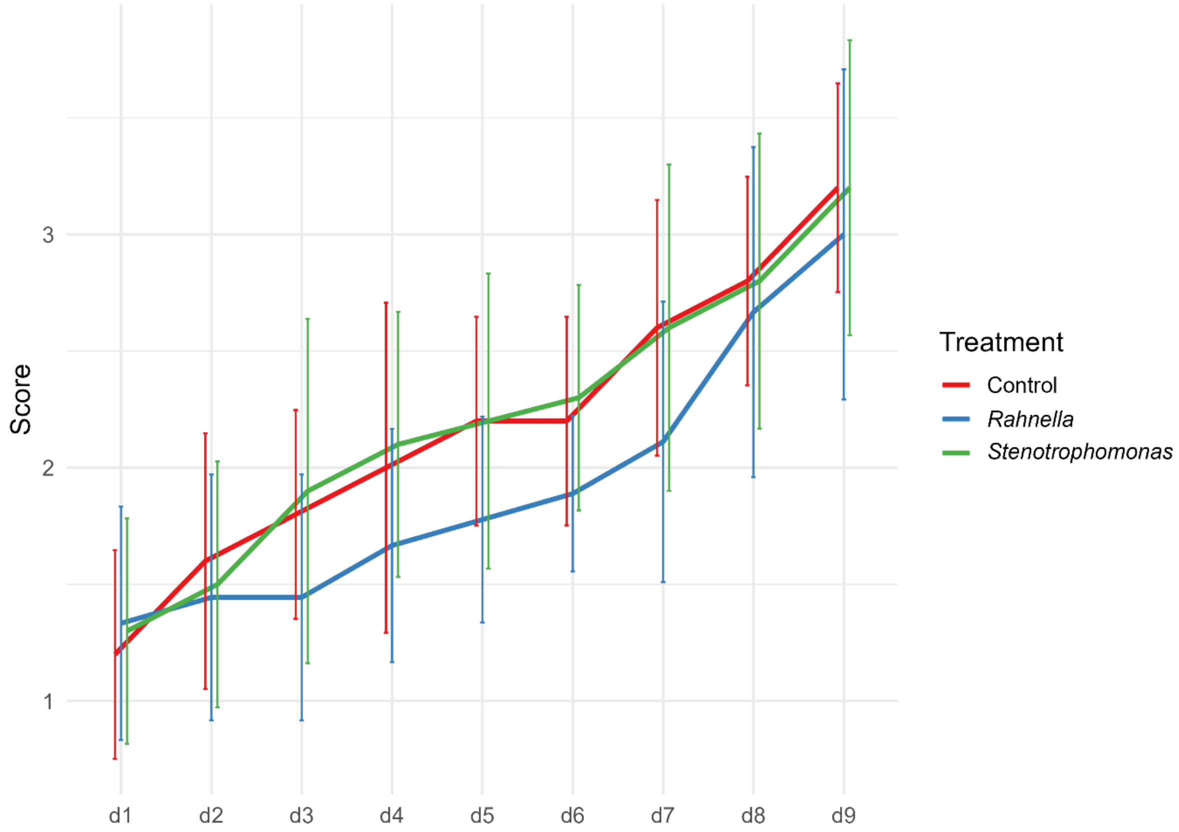

Figure S4. Performance scores of wheat plants under drought based on a daily visual assessment. The assessment started on the first day where any sign of drought stress were visible (d1). Days are indicated at the bottom of the plot. The main line corresponds to the mean score on each day, and error bars indicate standard deviation (SD). A slight horizontal shift is added to each treatment line to prevent overlapping of error bars.
